# Chronic exposure of humans to high level natural background radiation leads to robust expression of protective stress response proteins

**DOI:** 10.1038/s41598-020-80405-y

**Published:** 2021-01-19

**Authors:** S. Nishad, Pankaj Kumar Chauhan, R. Sowdhamini, Anu Ghosh

**Affiliations:** 1 Radiation Signaling Group, Bio-Science Group, Radiation Biology & Health Sciences Division, Bhabha Atomic Research Centre (BARC), Mumbai, 400 085 India; 2 Homi Bhabha National Institute (HBNI), Mumbai, 400 094 India; 3 Computational Approaches to Protein Science, National Centre for Biological Sciences (NCBS), Tata Institute for Fundamental Research (TIFR), Bangalore, 560 065 India

**Keywords:** Biochemistry, Cell biology, Natural hazards, Biomarkers

## Abstract

Understanding exposures to low doses of ionizing radiation are relevant since most environmental, diagnostic radiology and occupational exposures lie in this region. However, the molecular mechanisms that drive cellular responses at these doses, and the subsequent health outcomes, remain unclear. A local monazite-rich high level natural radiation area (HLNRA) in the state of Kerala on the south-west coast of Indian subcontinent show radiation doses extending from ≤ 1 to ≥ 45 mGy/y and thus, serve as a model resource to understand low dose mechanisms directly on healthy humans. We performed quantitative discovery proteomics based on multiplexed isobaric tags (iTRAQ) coupled with LC–MS/MS on human peripheral blood mononuclear cells from HLNRA individuals. Several proteins involved in diverse biological processes such as DNA repair, RNA processing, chromatin modifications and cytoskeletal organization showed distinct expression in HLNRA individuals, suggestive of both recovery and adaptation to low dose radiation. In protein–protein interaction (PPI) networks, YWHAZ (14-3-3ζ) emerged as the top-most hub protein that may direct phosphorylation driven pro-survival cellular processes against radiation stress. PPI networks also identified an integral role for the cytoskeletal protein ACTB, signaling protein PRKACA; and the molecular chaperone HSPA8. The data will allow better integration of radiation biology and epidemiology for risk assessment [Data are available via ProteomeXchange with identifier PXD022380].

## Introduction

The basic principles of low linear energy transfer (LET) ionizing radiation (IR) induced effects on mammalian systems have been broadly explored and there exists comprehensive knowledge on the health effects of high doses of IR delivered at high dose rates. But, the biological responses and health effects induced by low dose (≤ 100 mGy) and low dose rate (≤ 0.1 mGy/min) exposures of low LET IR^1^ have been difficult to characterize. The uncertainties associated with low dose radiation (LDR) significantly influences rational decision-making on medical practices, radiation protection policies, evacuation guidelines after large-scale nuclear events, long term clean-up of radiation contaminated sites and communication, in general, with members of public.

The risk estimations for adverse health outcomes (cancer and non-cancer) of IR have centered essentially on human epidemiology data. But due to the fact that the biological responses are generally subtle and sometimes obscured by inter-individual variation, these studies have low power of detection of risks for doses below 100 mSv. In view of this, extrapolations through linear no-threshold (LNT) model has been suggested for estimating risks of LDR exposures^2^. However, since the LNT model is based on assumptions that continue to be debated, the precise dose–response relationship for LDR remains uncertain. There is thus, a compelling need for research to understand mechanisms of action for low dose exposures in humans that may then facilitate better understanding of radiation risks, and of the factors that can influence risk. Several international bodies like UNSCEAR (United Nations Scientific Committee on the Effects of Atomic Radiation), MELODI (Multidisciplinary European Low Dose Initiative), NRC (U.S. Nuclear Regulatory Commission) and others strongly recommend and promote studies on biological mechanisms of low dose and low dose rate radiation exposures. An integration of epidemiology with molecular radiobiology seems to be the right way forward.

Background radiations from natural sources remain, by far, the largest contributor to the average dose received by the general public. According to UNSCEAR estimates, the global average annual dose from natural sources is 2.4 mSv, of which 0.48 mSv annual dose comes as external exposure from terrestrial sources^3^. However, there are some **h**igh-**l**evel **n**atural **r**adiation **a**reas (HLNRAs) of the world where the radiation exposures from terrestrial sources is high due to local geology and geochemistry, leading to chronic high background exposure situations^4^. In India, the 55-km (34.17 mi) long and 0.5-km (0.31 mi) wide belt on the coast of Arabian Sea in the southwest state of Kerala show natural background radiation levels that are up to 20 times higher than the global average effective dose^3,5^. This coastal belt which stretches between Neendakara (Kollam district) in south to Purakkadu (Alappuzha district) in north is one of the well-studied HLNRA of the world. The elevated level of terrestrial radiation in these coastal sands is due to placer deposits of monazite, a mineral which primarily contains thorium oxide (8–10% weight average), uranium oxide (0.3% weight average) and their decay products. Due to the non-uniform deposits of monazite, the background radiation doses in this area range from ≤ 1 to ≥ 45 mGy/y, providing an ideal opportunity for dose–response studies^5^. As compared to the other similar regions of the world, HLNRA of Kerala has high population density and has been inhabited for generations^6^. It thus, serves as a valuable resource to understand biological effects of chronic low dose/low dose rate radiation directly on healthy individuals. Since the normal background radiation in the adjoining coastal areas of Kerala range between < 1.0 to 1.5 mGy/y, the areas with radiation exposures below 1.5 mGy/y have been classified as **n**ormal **l**evel **n**atural **r**adiation **a**reas (NLNRA), while those above 1.5 mGy/y are considered as HLNRA^7^. Studies conducted over several years in this area using various endpoints have not shown any adverse health effects in individuals residing in HLNRA as compared to NLNRA^7–10^, although the underlying biological mechanisms remain unclear.

Proteins are considered functional molecules of the cell and thus, relate better to the phenotype^11^. During cellular stress, specific alterations of the proteome may enable cells to adapt and respond. Analysis of these alterations in protein abundance can hence, provide useful information on biological functions, and consequently on health effects, associated with LDR. To date, no proteomic study has been conducted to understand alterations in human proteome in response to chronic LDR, although a modest number of published reports investigated proteomic alterations with acute in vitro radiation exposures in human cells^12–14^. The present study thus, focused on discovery proteomic approach using four-plex iTRAQ-labelling (Isobaric Tags for Relative and Absolute Quantification) and nanoscale liquid chromatography coupled to mass spectrometry (nano-LC–MS/MS) to recognize potential proteomic signatures of chronic LDR in human peripheral blood mononuclear cells (PBMCs) derived from HLNRA individuals. The objective was to establish a benchmark dataset of proteins to evaluate true responses of chronic radiation directly in humans with no ‘a priori’ hypothesis.

## Results and discussion

Primary human PBMCs, which are in the G_0_ non-dividing stage, are recognized to be radiosensitive cells and represent the in vivo milieu effectively. Our previous work with two-dimensional gel proteomics has shown that human PBMCs respond to IR with subtle, but specific changes in the proteome^15,16^. In this work, to define changes in response to chronic LDR, we compared relative changes in protein expression reflecting either synthesis or degradation, on pooled human PBMCs collected from individuals residing in HLNRA (≥ 1.5 mGy/y), vis-à-vis NLNRA (≤ 1.5 mGy/y). Pooling of PBMCs from randomly selected healthy individuals allowed us to not only overcome resource constraints of analyzing large number of samples, it also reduced random biological variance^17^. For analysis, samples from NLNRA were classified as Group I (≤ 1.5 mGy/y), while samples collected from HLNRA were stratified into three random user-defined radiation dose groups depending on the annual dose received by the individuals: Group II [range 1.5–5.0 mGy/y]; Group III [range 5.01–14.0 mGy/y]; Group IV [≥ 14.01 mGy/y] (Supplementary Table S1). In each dose group, ten samples (biological replicates) were pooled and analyzed in duplicate (technical replicates). Tryptic peptides of PBMCs were labeled with iTRAQ 4-plex reagent, analyzed with LC–MS/MS, and relative protein ratios were calculated.

### Quantitative proteomics using iTRAQ based LC–MS/MS

**Figure 1:**
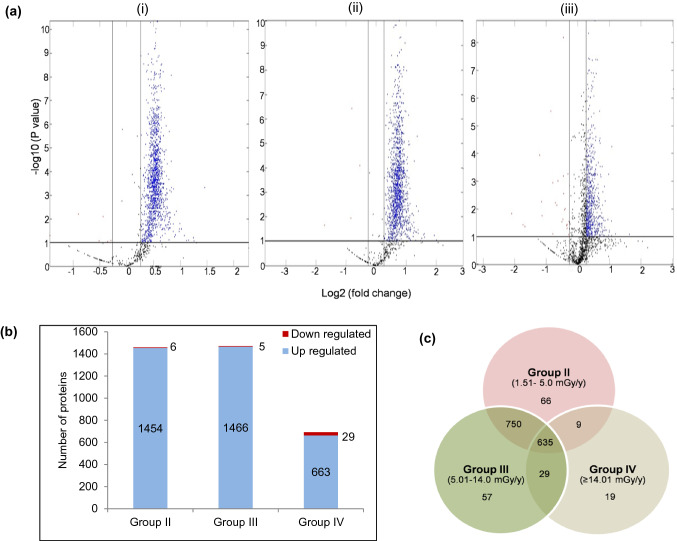
Quantitative iTRAQ based LC–MS/MS analysis of human PBMCs from individuals residing in HLNRA. (**a**). Volcano plots showing adjusted P values (− log10) *versus* protein ratio (log2) for (i) Group II (ii) Group III (iii) Group IV HLNRA dose groups. Blue dots represent significant up-regulated genes (iTRAQ ratio ≥ 1.2, adjusted P < 0.1) and red dots represent significant down-regulated genes (iTRAQ ratio ≤ 0.83, adjusted P < 0.1). Protein dots represented in black were not differentially altered. (**b**). Number of differentially regulated proteins in the three HLNRA dose groups. The numbers on the graph show up-(blue) and down-(red) regulated proteins. (**c**). Venn diagram of unique and overlapping differentially regulated proteins in the three HLNRA dose groups.

A total of 15,073 peptides were identified with the iTRAQ LC–MS/MS analysis which were matched to 4166 unique PBMC proteins. Of these, 1699 proteins contained sufficient iTRAQ signal for relative quantitation. In biological systems, even small changes in expression levels of proteins can regulate many cellular functions at the molecular level^18^. In order to avoid exclusion of proteins with subtle yet functionally relevant changes in abundance, differential proteins chosen by a ratio of ≤ 0.83 or ≥ 1.2 with an adjusted *P*-value of ≤ 0.1 [Benjamini-Hochberg (BH) correction] in at least one dose group, were selected for further analysis (Fig. 1a). Nevertheless, ~ 96% proteins in Group II, ~ 95% proteins in Group III and ~ 85% proteins in Group IV dose groups of HLNRA had adjusted *P*-values ≤ 0.05 (Supplementary Fig. S1a). More than 79% of differentially regulated proteins in each dose group showed coefficient of variation (% CV) value ≤ 20%, indicating good stability among the technical replicates (Supplementary Fig. S1b). The complete list of differentially regulated proteins with accession numbers and brief descriptions is given in Supplementary Table S2. Our dataset was comparable to an earlier PBMC map of 4129 proteins generated with a TMT (tandem mass tag) based LC–MS/MS gel-free proteomics approach^19^. Over 72% of differential proteins were represented by ≥ 2 peptide matches and ~ 61% of them showed more than 5% peptide sequence coverage (Supplementary Fig. S1c,d). Comparative analysis identified 1460 differential proteins for Group II *vs.* Group I (1454 up-regulated and 6 down-regulated), 1471 differential proteins for Group III *vs.* Group I (1466 up-regulated and 5 down-regulated) and 692 differential proteins for Group IV *vs.* Group I (663 up-regulated and 29 down-regulated) comparison (Fig. 1b). Of these, 66, 57 and 19 proteins that changed in abundance in response to LDR were unique to Group II, Group III and Group IV of HLNRA, respectively indicating small but distinct qualitative differences between the three dose groups (Fig. 1c). The number of altered proteins decreased sharply in Group IV individuals, indicating possible selective translational regulation during LDR stress^20^.

### Functional enrichment of differentially regulated proteins

**Figure 2:**
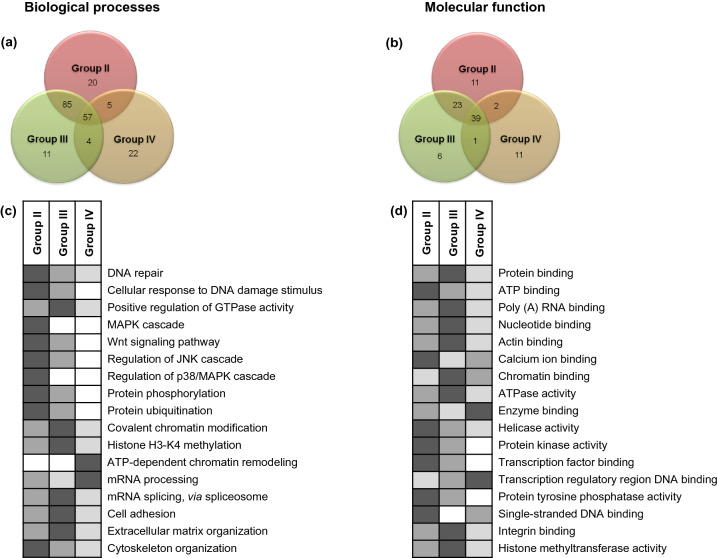
Functional classes of proteins altered with chronic low dose radiation in the three HLNRA dose groups relative to NLNRA dose group. Venn diagram of unique and overlapping. (**a**) Biological processes (GO analysis) and (**b**) Molecular function (GO analysis). Comparative analysis of representative enriched categories for the three HLNRA dose groups. (**c**) Biological processes GO terms and (**d**) Molecular function GO terms. The intensity of shading indicates significance (Fisher’s exact test P-value) of enriched GO categories with the darkest grey intensity showing the lowest P-value. All gray boxes have a Fisher’s exact test P-value of ≤ 0.05; white boxes indicate enriched GO categories with non-significant P-values (≥ 0.05).

The DAVID functional annotation bioinformatics resource was used for over-representation analysis where the proteins that changed in abundance were grouped on the basis of their function and potential role in molecular pathways. Fisher’s exact test (*P* ≤ 0.05) was used to assess the statistical significance of protein enrichment in annotation terms (Fig. 2a,b). Proteins with similar or identical functions were grouped together. The Gene Ontology (GO) functional annotation revealed that the number of over-represented biological processes decreased as the annual background dose received by the individuals increased (Group II: 167 processes; Group III: 157 processes; Group IV: 88 processes, relative to Group I). The enriched functional categories were then filtered for their relevance with radiation response through manual citation search in PubMed (https://www.ncbi.nlm.nih.gov/pubmed) in keeping with our research objective. Expression trends in the three HLNRA groups for some of these relevant biological processes as well as molecular function categories defined by confidence values (P-values) are shown in Fig. 2c,d. The analysis revealed that most pathways over-represented in HLNRA samples belonged to the stress response categories, such as DNA damage response, RNA processing, chromatin modifications, cytoskeletal organization, cell signaling and protein modifications. Many of these processes were found globally in all the three HLNRA groups; but the number of proteins in each category and the level of differential regulation (relative expression levels or fold-changes) were distinct for each group (Supplementary Table S2). Few biological processes showed a distinct dose-specific enrichment. In the molecular function ontology, more than 75% of the proteins belonged to protein, RNA, DNA and chromatin binding.

#### Enriched biological processes related to DNA damage response (DDR)

In this study, many proteins that changed in abundance in HLNRA samples were annotated as being involved in DDR. This included proteins associated with DNA damage repair and with DDR signaling. The repair proteins that showed differential modulation belonged to all major DNA repair pathways: BER or base excision repair (APEX2, MBD4, HUWE1); NER or nucleotide excision repair (XPF, RPA1, CUL4A); MMR or mismatch repair (MSH3, MLH1); HR or homologous recombination repair (BLM, BRCA2, FANCI, FANCA, FANCM, RA54B, RMI2, PARI); NHEJ or non-homologous end joining (XRCC6, PRKDC, UVRAG, SFPQ) and TLS or translesion synthesis (DPOLQ, DPOLN). Within these, proteins involved in HR repair showed highest relative fold changes. Some of the prominent proteins of DDR signaling that showed up-regulation in HLNRA individuals as compared to NLNRA were histone acetyltransferase cofactor TRRAP that associates with the MRN repair complex and modulates DNA double-strand break (DSB) repair^21^; ATAD5 and EEPD1 involved in rescue of stressed replication forks and maintenance of genome stability^22,23^; ATR, which is the DDR kinase^24^; ATMIN, which is a cofactor of DNA damage kinase ATM^25^; ATRX, involved in telomeric DSB repair and HR repair^26,27^; and EMSY, a BRCA-2 associated protein involved in chromatin regulation and HR repair^28^. On the other hand, there were many proteins that showed up-regulation in Group II and Group III individuals but not in Group IV individuals. Examples of some of these proteins were mTOR and SMG1, the serine/threonine kinases of the PIKK (phosphoinositide kinase-related kinase) family; INO80, a chromatin remodeling ATPase; and CDN1A, the cyclin-dependent kinase inhibitor.

#### Enriched biological processes related to RNA processing

All three HLNRA groups showed marked enrichment (*P* ≤ 0.05, Fisher’s exact test) of biological processes related to RNA processing and splicing. There was a differential expression of several heterogeneous nuclear riboproteins or hnRNPs (e.g. HNRH2, HNRL1, HNRPL, HNRPM, HNRPU, ROA1, ROA3, ROA0), splicing proteins (e.g. SF3B3, SF3B4, SFPQ, SFR19, SF3B3, SF3B4) and RNA helicases (e.g. DHX29, DHX33, DHX36) in HLNRA samples. As a large family of RNA-binding proteins, hnRNPs are known not only for their role in regulation of alternative splicing, mRNA stability, transcription and translation, but also in regulation of cell signaling, DNA repair and telomere biogenesis^29^. Several RNA processing proteins with central roles in DNA repair e.g. RBMX, FUS, SF3B4, p54nrb/NONO, SFPQ, EWS and DHX36 were also over-expressed in HLNRA groups. This is suggestive of an intricate functional link between DNA damage response and RNA metabolism during low dose exposures, in line with other recent findings^30^.

#### Enriched biological processes related to cytoskeleton organization, chromatin modifications and post-translational modifications (PTMs)

Biological processes related to cytoskeleton organization were over-represented in all three HLNRA groups. Examples of proteins from this group included ACTB, MAST2, MAST3, MAST4, MAP6, K22E, K22O, and K2C4. Accumulating evidence now suggests that cytoskeleton proteins drive and regulate specific events of signal transduction through the PI3 kinase-Akt and Rho-Rock pathways^31^, which also showed clear representation in HLNRA individuals.

In eukaryotic cells, the chromatin architecture influences both damage formation as well as repair of DNA. Our data revealed chromatin modifications as one of the major biological process affected with LDR in HLNRA individuals. A differential change in expression was seen for several members of chromatin remodeling complexes from SWI/SNF and chromodomain helicase sub-family (e.g. SMRC1, CHD4, CHD7, CHD8 and CHD9) as well as for various histone lysine n-methytransferases (e.g. KMT2A, KMT2B, KMT2C, KMT2D) and lysine demethylases (e.g. KDM2B, KDM6A). Many zinc finger proteins (ZFPs) such as ZFHX3, ZN292, ZN296, ZN462, and ZN703 also showed higher representation in HLNRA individuals. Zinc finger domain is one of the most abundant DNA-binding motifs found in eukaryotic transcriptional factors and several ZFPs have been implicated in telomere maintenance, signal transduction and DNA repair^32^. Chromatin is now considered a dynamic participant in all processes that use DNA as the template to facilitate, regulate or terminate cellular responses.

Biological processes associated with protein phosphorylation and protein ubiquitination of target proteins formed major enriched categories in Group II and Group III HLNRA individuals, but not in Group IV individuals. Phosphorylation is carried out by protein kinases enzymes which were detected as the largest family of proteins in our dataset. Processes of both peptidyl-serine and peptidyl-tyrosine were enriched. Protein phosphorylation-dephosphorylation acts as a crucial molecular switch to regulate cell metabolism, cell division and signal transduction^33^. Ubiquitination too, plays a central, role in several cellular functions in particular, signal transduction, cell-cycle progression, DNA repair, transcriptional regulation, and most importantly, acts as the signal for proteasome-mediated protein degradation^34^. Various ubiquitin ligase (E3) enzymes that determine substrate recognition and specificity were also detected as differentially regulated proteins (e.g. HERC2, HUWE1, RBBP6, RN169, RN19A, UBR5, ZNRF3). Our results thus, signify that these two reversible PTMs may be a versatile way to calibrate cellular responses to LDR.

#### Enriched biological processes related to cell signaling

Interestingly, cell signaling processes showed a dose-specific enrichment among the three groups of HLNRA. Biological processes like intracellular signal transduction cascade was uniquely enriched in Group II and Group III samples; differentially regulated proteins included ADCY1 which regulate cAMP signaling; Trio, a classical guanine nucleotide-exchange factor that links G protein coupled receptors (GPCRs) to the MAPK signaling pathway; and MRCKs (MRCKA and MKCKG) which are activated by Cdc42·GTP and regulate signaling related to cell cycle checkpoint and actin remodeling^35^. Two major mammalian intersectins (ISTN1 and ISTN2) which act as scaffold proteins and regulate protein kinases like MAPK also showed over-expression. ISTN1 also regulates Ras family GTPases such as CDC42 and Ras^36^. Many serine/threonine protein kinases (e.g. DCLK1, MAST2, MAST3 and MAST4) also showed increased abundance in Group II and Group III individuals. Recent reports have emphasized critical role of DCLK1 in DDR and its potential as a radiation mitigator in radiotherapy^37^. The protein DPTOR, a negative regulator of mTOR kinase activity, was one of the very few proteins that were down-regulated in Group II individuals.

Group II and Group III HLNRA individuals also showed unique enrichment of the canonical Wnt signaling pathway. Many studies have linked activation of Wnt/β-catenin signaling pathway to radioprotection, especially of salivary gland, oral mucosa, and gastrointestinal epithelium through inhibition of apoptosis and preservation of normal tissue functions^38^.

Among the stress activated protein kinase pathways, MAPK and p38-MAPK cascades were enriched in Group II, while the JNK cascade was enriched in Group III HLNRA individuals. The phosphatidylionositol mediated signaling pathway was enriched only in Group II samples.

### Pathway enrichment of differentially regulated proteins

**Figure 3:**
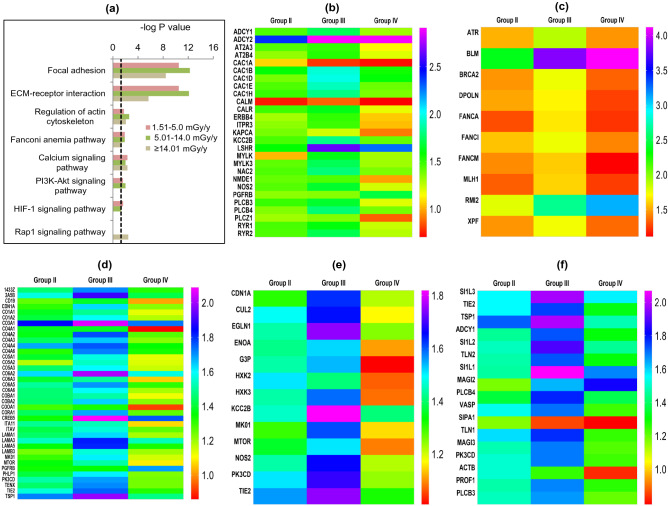
Pathway enrichment analysis of differentially regulated proteins in the three HLNRA dose groups relative to NLNRA dose group. (**a**) Enrichment of selected KEGG pathways (https://www.kegg.jp/kegg/). The dotted line represents significant enrichment degree cut-off [− log10 (0.05), Fisher’s exact test] of each GO term. Representative heat maps for expression level of individual proteins of selected KEGG pathways. (**b**) Calcium signaling pathway, (**c**) Fanconi anemia pathway, (**d**) PI3-Akt signaling pathway, (**e**) HIF signaling, (**f**) Rap1 signaling.

To further facilitate biological interpretation, molecular pathways associated with the differential proteins were identified using KEGG (Kyoto Encyclopedia of Genes and Genomes; https://www.kegg.jp/kegg/) analysis tool of DAVID and as before, the enriched pathways were perused for their linkages to radiation response through manual referencing in PubMed. KEGG enrichment identified several core pathways related to cell–matrix interaction, DNA damage repair and cell signaling (*P* ≤ 0.05, Fisher’s exact test) in all the three HLNRA dose groups (Fig. 3a). Again, the level of expression of individual proteins was discrete in each group, as depicted in the heat maps of proteins from selected pathways Fig. 3b-f. Interestingly, most proteins showed highest expression levels for Group III individuals (dose group 5.01–14.0 mGy/y).

The enriched cell–matrix interactions related pathways included proteins belonging to ECM-receptor interaction, focal adhesion and actin cytoskeleton regulation. Most of these proteins showed up-regulation and included collagens, laminin, protein kinases, filamins, talin, vinculin, integrin receptors and several cytoskeletal proteins, as described in the previous section. There are many reports that show concerted interactions between ECM, integrins, actin cytoskeleton and chromatin which favors DNA repair and genome stability for increased cell survival after radiation^39^.

The calcium signaling pathway showed enrichment in all the three HLNRA groups (Fig. 3b). Calcium is a versatile secondary messenger and the alteration of the intracellular calcium levels is known to affect several cellular functions such as secretion, enzyme activation, exocytosis, cell cycle regulation, unfolded protein response, apoptosis and gene transcription^40,41^. Among the proteins that showed differential regulation in HLNRA-NLNRA comparison were the signal transduction protein calmodulin; calcium binding chaperone calreticulin; calcium/calmodulin-dependent protein kinase; adenylate cyclase proteins; voltage-dependent calcium channel proteins and phosphoinositide-specific phospholipase C family proteins.

Another pathway annotated with KEGG analysis was the Fanconi-anemia (FA) pathway that showed enrichment and over-expression in all the three HLNRA dose groups (Fig. 3c). FA proteins essentially mediate repair of genotoxic stress induced DNA interstrand crosslinks through a coordinated mechanism that engages proteins from other major DNA repair pathways. In non-replicating cells, these proteins promote alternate end joining (A-EJ) repair over classical non-homologous end-joining (C-NHEJ)^42^. They are also involved in processing of transcription associated R-loops and stabilization of replication forks^43,44^. Additional cytoprotective roles of FA pathway proteins from cell death induced by ROS and pro-inflammatory cytokines is also reported^45^. Our data showed an over-expression of ten FA pathway proteins (ATR, BLM, BRCA2, DPOLN, FANCA, FANCI, FANCM, MLH1, RMI2, and XPF) in HLNRA individuals. Constitutive activation of several canonical and non-canonical FA proteins seen in the present work may thus, provide a rationale for lower incidence of basal level DNA damage in lymphocytes of HLNRA individuals from Kerala as seen with earlier studies^46,47^.

On the other hand, three primary pro-survival signaling pathways identified by KEGG namely, PI3K-Akt signaling pathway, HIF-1 signaling pathway and Rap1 signaling pathway showed a radiation dose-specific enrichment (*P* ≤ 0.05, Fisher’s exact test) (Fig. 3d–f). The PI3K-Akt signaling and HIF-1 signaling pathways showed enrichment only in Group II and Group III HLNRA individuals. The PI3K-Akt signaling actively promotes cell survival by activating many pro-survival proteins and inhibiting pro-apoptotic proteins. The IR activated PI3K-Akt signaling has been widely reported in different cell types^48,49^. Our data suggests that integrin mediated signaling via PI3K/Akt and MAP kinase may be an important regulator of cell survival in response to LDR in human PBMCs. The HIF signaling is modulated by cellular redox conditions and regulates many pro-survival or pro-death factors depending on the cellular stress conditions^50^. As opposed to HIF and PI3k-Akt, Rap1 signaling pathway was enriched only in Group IV individuals. Rap1 is a ubiquitous protein and is a member of Ras-superfamily of small molecular weight GTPases. Mammalian Rap1 isoforms are best recognized as regulators of integrin-based cell matrix adhesion but also regulate cytoskeleton remodeling, invasion, metastasis and MAP kinase activity^51^. However, the significance of Rap1 enrichment only in individuals in the radiation dose range > 14 mGy and not at lower doses need to be probed further.

### Interactome network analysis

**Figure 4:**
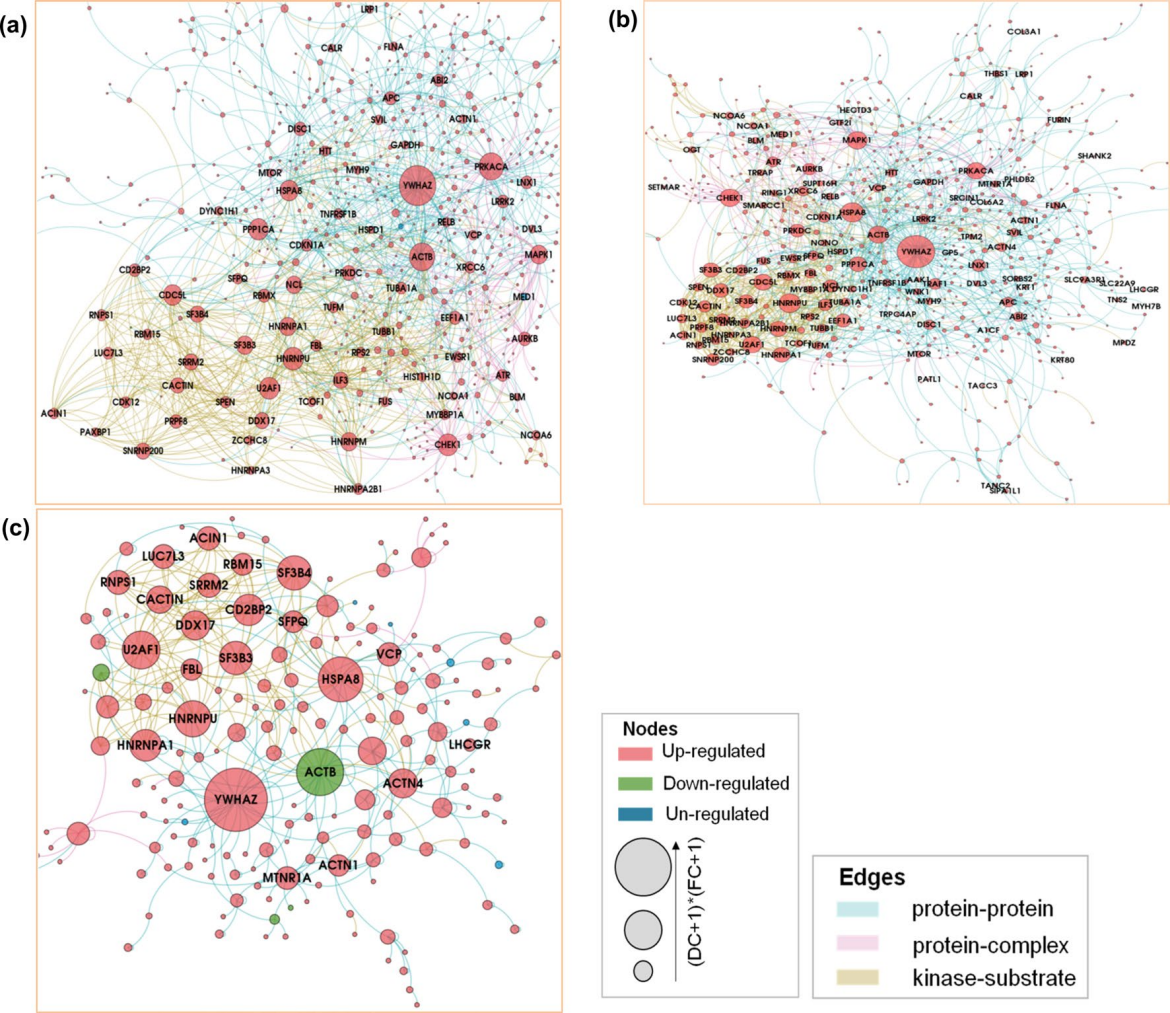
Protein–protein interaction network analysis of the differentially regulated proteins in the three HLNRA dose groups relative to NLNRA dose group. The Giant connected component (GCC) of protein–protein interaction network obtained from (**a**) Group II, (**b**) Group III, (**c**) Group IV HLNRA dose groups. The colours of the nodes indicate the modulation pattern (up-regulated, down-regulated, or un-regulated) of the proteins and the edges are coloured according to the source of the interaction. The network is laid out by multiplicative effect of fold change (FC) and Degree centrality (log2FC*Degree). High resolution portable network graphics (.png) versions of the interaction networks for Group II, Group III and Group IV HLNRA dose groups can be found as Supplementary Fig. S2a–c.

**Figure 5:**
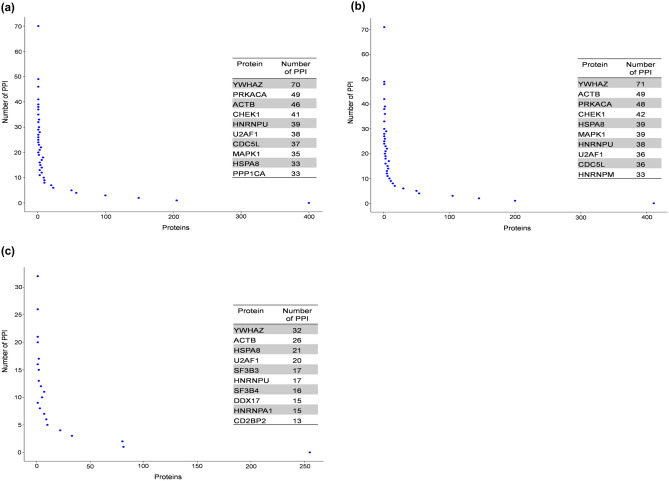
Distribution of protein–protein interactions (PPI) for the hub proteins of the network for (**a**) Group II, (**b**) Group III and (**c**) Group IV HLNRA individuals. The table on the graph indicates the number of PPIs for top 10 hub proteins of the network. The hub proteins were identified based on multiplicative effect (log2FC*Degree) of fold change (FC) and Degree centrality (DC scores).

Recent estimates suggest that potentially there may be ≈ 53,000–650,000 protein–protein interactions (PPIs) possible in humans cells^52,53^. Consequently, mapping of these PPIs can provide a global view of complex molecular interactions in living systems. We used differentially regulated proteins to construct PPI networks by searching against various interactome databases (Fig. 4). High resolution portable network graphics (.png) versions of the interaction networks for Group II, Group III and Group IV HLNRA dose groups can be found as Supplementary Fig. S2a–c. The PPI analysis was able to construct networks using 1106 proteins of Group II (out of 1460 differentially regulated proteins), 1126 proteins of Group III (out of 1471 differentially regulated proteins) and 528 proteins of Group IV (out of 692 differentially regulated proteins) of HLNRA. The nodes in the PPI networks represent proteins, while edges characterize the number of interactions between them. A total of 1106 nodes connected by 1703 edges were identified in Group II individuals, 1126 nodes connected by 1780 edges in Group III individuals and 528 nodes connected by 497 edges in Group IV HLNRA samples (Supplementary Table S3). The size significance of the largest cluster of the network, the giant connected component (GCC) showed significant scores with 587 nodes in Group II (Z-score: 5.5, P-value: 0.0001), 604 nodes in Group III (Z-score: 5.7, P-value: 0.0001) and 178 nodes in Group IV samples (Z-score: 3.6, P-value: 0.0001) (Fig. 4). The top ten ‘hub’ nodes based on multiplicative effect (log_2_FC*Degree) of fold change (FC) and Degree centrality (DC scores) that may represent prime proteins of the network were identified (Fig. 5, Supplementary Table S4).

The phosphoserine/threonine-binding adaptor protein YWHAZ (also known as 14-3-3ζ) that integrates and controls multiple signaling pathways, emerged as the top-most hub protein in all the three HLNRA groups. An early work using affinity chromatography and tryptic mass fingerprinting of phosphorylated HeLa proteins that bind to 14-3-3s identified more than 200 candidate targets with potential roles in cellular biosynthesis, antioxidative processes and actin dynamics, along with proteins that are deregulated in cancer, diabetes and neurological disorders^54^. Emerging evidence suggests that genotoxic stress induces diverse interactions among the 14-3-3 proteins as well as with several other DNA damage-responsive proteins. Most such DNA damage induced interactions influence cell cycle checkpoint activation or cell death pathways^55^. Other studies have defined additional roles of 14-3-3 proteins in cellular processes like protein trafficking, DNA replication and transcriptional regulation^56^. More specifically, a highly context-dependent role of 14-3-3ζ interactome under various stress conditions has been suggested. For instance, a LC–MS analysis of HEK-293T cells showed dynamic rearrangement of 14-3-3ζ interactome under hypoxic conditions^57^, while plasma proteomics identified 14-3-3ζ as a hypoxia stress-responsive protein in an ischemic-hypoxic rat model^58^. The present study indicates that 14-3-3ζ may be the key player as the stress adaptive hub protein in the DNA damage response to LDR.

The cAMP-dependent protein kinase, catalytic alpha, PRKACA was another important hub protein in Group II and Group III individuals of HLNRA. This active kinase regulates the cAMP-mediated second messenger signaling by phosphorylation of downstream target proteins^59,60^. Studies have also linked cAMP signaling with efficiency of recruitment of protein XPA (xeroderma pigmentosum complementation group protein A) to the UV and cisplatin damaged sites for nucleotide excision repair (NER)^61^. Functionally, XPA acts as an integral limiting factor in damage recognition for both the sub-pathways of NER namely, global genomic-NER (GG-NER) and transcription-coupled NER (TC-NER)^62^. Our study suggests that PRKACA-cAMP signaling axis may be important for NER repair of low dose IR-induced damage also. Significantly, PRKACA was not detected as a differentially regulated protein in Group IV individuals, suggesting that the cAMP mediated regulatory mechanism may be dose-dependent.

The cytoskeletal protein ACTB (Actin, cytoplasmic 1) and the molecular chaperone HSPA8 (Heat shock 70 kDa protein 8) appear as other top proteins in the network for all three HLNRA groups. Many recent studies have underlined regulatory functions of actin filaments in response to DNA damage, especially at the DNA repair sites^63^. However, in Group IV, ACTB was down-regulated, which again may indicate radiation dose-specific response. HSPA8 is involved in proper folding, trafficking, assembling and degradation of proteins under various stress conditions. It is thus, central to many processes and essential for cellular homeostasis^64^.

Proteins that act as leading regulators of cell cycle [CHEK1, checkpoint kinase; CDC5L, cell division cycle 5-like protein] and for the signal transduction and amplification of environmental stimuli [MAPK1, mitogen-activated protein kinases 1] were some of the proteins highlighted in PPIs of Group II and III, but not in Group IV. Interestingly, proteins that are involved in pre-mRNA splicing (SF3B3 and SF3B4) showed significant interactions and appear among top ten hub proteins only in the highest dose Group IV. It is now becoming increasingly apparent that genotoxic stress can regulate alternate splicing of pre-mRNAs for ‘gene-silencing’ or ‘non-productive’ splice variants^65^. This may either result in reduced gene expression or restricted protein expression, as was evident in Group IV samples.

We however, recognize that proteins uniquely identified in one HLNRA dose group may not mean a complete absence in other HLNRA groups; their absence may be only a reflection of abundance lower than the detection limits of our approach or the threshold filters applied for significance testing. Nonetheless, it still specifies differential changes in abundance.

### mRNA expression of selected proteins

**Figure 6:**
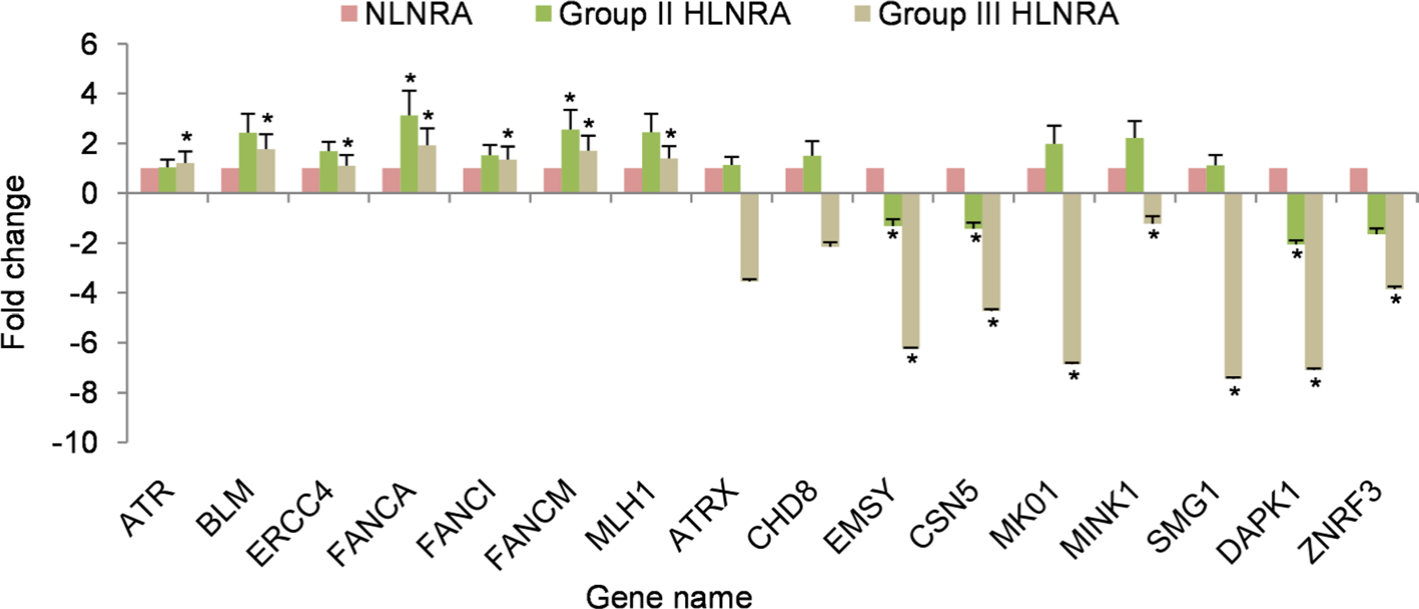
Real time qPCR analysis for 16 candidate proteins identified with iTRAQ. The bars correspond to relative fold change values represented as mean ± SEM (n = 3). Symbol (*) represents statistical significance calculated by Student’s t-test (*P* ≤ 0.05) in individuals from HLNRA dose groups (N = 5), relative to individuals from NLNRA dose group (N = 5).

The expression levels of 16 randomly selected proteins were analyzed at the mRNA level using RT-PCR on an additional set of samples collected from NLNRA (Group I) and HLNRA (Group II and Group III) (Supplementary Table S5). Samples from HLNRA Group IV could not be collected. The candidate genes belonged to biological processes such as DNA repair (ATR, BLM, ERCC4, FANCA, FANCI, FANCM, MLH1), chromatin modifications (ATRX, CHD8, EMSY), MAP kinase cascade (CSN5, MK01), post-translational modifications (MINK1, SMG1), apoptosis (DAPK1), and WNT signaling (ZNRF3). Real-Time quantitative Polymerase Chain Reaction (RT-qPCR) was performed on LC480 Real-time PCR machine (Roche Diagnostics, GmbH, Germany). Two endogenous reference genes β-actin and GAPDH were used to normalize the expression of target genes. As per the MIQE (Minimum Information for Publication of Quantitative Real-Time PCR Experiments) guidelines^66^, we first analysed the threshold cycle values (Ct) for both the reference genes to validate their suitability for the cell system and specific experimental conditions used in our study (Supplementary Fig. S3). Both β-actin and GAPDH showed high expression, good stability and minimal variation in the PBMCs system. Among the candidate genes, all the seven DNA repair genes showed up-regulated mRNA expression in both Group II and Group III HLNRA individuals relative to NLNRA individuals and the response was consistent with the iTRAQ data. In contrast, expression of genes like ATRX, CHD8, MK01, MINK1 and SMG1 correlated with iTRAQ data for Group II but not for Group III individuals. For other genes, mRNA expression varied from iTRAQ data (Fig. 6). In mammalian cells, global correlation between protein and mRNA concentration remain characteristically poor, sometimes as low as 40%^67^. Multiple processes such as stability of mRNA, half-life of protein, translation rates, post-transcriptional and post-translational mechanisms, miRNA regulation etc. may contribute to this lack of correlation^68^.

## Conclusions

This work presents, for the first time, an expansive list of human proteins altered in abundance with chronic LDR. We observed an increasing number of proteins and biological processes being affected in Group II (doses 1.5–5.0 mGy/y) and Group III (doses 5.01–14.0 mGy/y) and then decline in Group IV (doses ≥ 14.01 mGy/y). This might be suggestive of a threshold dose for chronic radiation. The small sample set however, limits our interpretation of individual variations in radiation sensitivity. Although the identified proteins need to be substantiated using functional assays, we were able to show a distinct basal expression of several ‘pro-survival’ proteins involved in DNA damage signaling, DNA repair, chromatin modifications, RNA processing, and proteostasis, amongst others. This suggests that apart from DNA damage response, a multitude of other responses with interconnected circuitry are equally essential for recovery and adaptation to chronic radiation. Eventually, candidate proteins will be validated with targeted proteomics to identify persistent dose-dependent signatures and radiation biomarkers for molecular epidemiology. Nonetheless, the large scale proteomic changes observed provide a molecular basis for the lack of genomic instability^69,70^ and indication of radio-adaptive response^16,71^ reported earlier in HLNRA individuals. A previous study from our group showed that HLNRA PBMCs when challenged with an ex-vivo dose of 2 Gy respond with induced expression of several critical proteins to maintain adaptive homeostasis^16^. Similar evidence of radiation-induced adaptive response has been shown in individuals residing in HLNRA of Ramsar, Iran with micronuclei frequency^72^. The present data gives a further perspective on some of the established radiobiological paradigms and echoes apprehensions on the validity of LNT model for LDR exposures. Integration of these mechanistic studies with epidemiological data will provide valuable insights into the low end of the dose–response curve for risk characterization and management.

## Materials and methods

### Materials

The sequencing grade modified trypsin for in-solution digestion was purchased from Promega, Madison, WI, USA. All iTRAQ reagents and buffers were procured from AB Sciex, MA, USA. Bio-Basic strong cation exchange (SCX) HPLC columns were purchased from Thermo Scientific, Waltham, MA, USA. The Zip-Tip C18 cartridges were purchased from Millipore Corp., MA, USA. All other chemicals and reagents were procured from Sigma-Aldrich Corp. MO, USA, unless indicated otherwise.

### Ethics statement

The study was approved by the institutional Medical Ethics Committee, Bhabha Atomic Research Centre (BARC), Mumbai, India (BHMEC/NP/32/12). Blood samples were collected from healthy volunteers by a trained hospital staff in accordance with the study protocol, methods and guidelines approved by the ethics committee. Written informed consent was obtained from each individual. Additional information on potential confounders like smoking, late onset diseases and exposures to other sources of radiation was also collected from each donor using a standard questionnaire outlined by BARC guidelines.

### Collection of blood and isolation of PBMCs for LC–MS/MS

Approximately 10–12 ml of venous blood was collected from random healthy male volunteers in sterile EDTA lined vacutainers (BD™ Vacutainers, NJ, USA) using standard venipuncture. Ten samples each were chosen from respective dose group. The age of the samples ranged between 26 and 49 years (Supplementary Table S1). The PBMCs were isolated from whole blood using density gradient centrifugation with Histopaque-1077 according to the protocol published earlier^15^.

### Dosimetry

The external gamma radiation levels (absorbed doses in air; μR/h) in each subject’s house was measured using halogen quenched Geiger Muller (GM) tube-based survey meter (Type ER-709, Nucleonix Systems, India), placed at a height of 1 m above the ground level, which were then converted into annual absorbed dose (mGy/y). The indoor radiation measurements were taken from the room with maximum occupancy and outdoor measurements were taken at a distance of 3 m from the main entrance of the subject’s dwelling. A mean of three readings was taken for each measurement. A conversion factor of 0.0767 (= 0.8763 × 24 h × 365 days × 10^−5^) was used to convert μR/h into mGy/y. The age- and sex-specific occupancy factor used for the calculation were according to an earlier study^10^. The internal radiation dose exposure from the ingested and inhaled radionuclides was not considered during the study.

### Preparation of protein extracts

Protein extracts were prepared from PBMCs by sonication according to a previously published method^15^ and subjected to in-solution tryptic digestion. For digestion, pooled proteins (100 μg) from each experimental group were treated with denaturant (2% SDS) for 1 h at room temperature. The disulfide bonds of proteins were reduced with 50 mM tris-(2-carboxyethyl) phosphine (TCEP) at 60 °C for 1 h. Alkylation was performed for 30 min in dark by replacing the TCEP solution with cysteine-blocking reagent (200 mM iodoacetamide). Proteins were then digested with trypsin with a substrate-to-enzyme ratio of 20:1 overnight at 37 °C. The digested peptides were concentrated using a vacuum centrifuge (Eppendorf, Hamburg, Germany).

### iTRAQ labeling and LC MS/MS analysis

The tryptic peptides were labelled with isobaric tags using iTRAQ Reagent Multiplex kit (AB Sciex, MA, USA). Label 114 was used for Group I pool samples (NLNRA), and labels 115, 116 and 117 for Group II, Group III and Group IV pools of HLNRA samples, respectively. The iTRAQ-labelled peptides were reconstituted in strong cation exchange (SCX) buffer A (5 mM KH_2_PO_4_ + 25% ACN, pH 3.0) and fractionated using BioBasic SCX (150 mm × 4.6 mm, 5µ particle size, 300 Å pore size) columns using a 75-min gradient at a flow rate of 1.0 ml/min. Retained peptides were eluted using the following gradient: 30-min with 100% Buffer A, followed by a linear increase to 25% Buffer B (5 mM KH_2_PO_4_ + 25% ACN + 350 mM KCl, pH 3), a second linear increase to 100% B for 10-min, and a final equilibration with 100% Buffer A for 15-min. A total of ten fractions were collected based on the retention time values and desalted with Zip-Tip C18 cartridges. Each SCX purified peptide fraction was re-dissolved in 0.1% formic acid and analyzed on a Q-TOF Synapt G2 High Definition mass spectrometer connected to a nano-ACQUITY UPLC system (Waters Corp., MA, USA). The peptide fractions were injected into LC system with nano-ACQUITY UPLC BEH C18 column (Internal diameter—75 µm, Length—150 mm, Particle size—1.7 µm, Column pore size—130 Å, pH range—2 to 10, Mode-reversed phase) for analysis. The eluted peptides were directed into Q-TOF instrument for mass spectrometric analysis for 200 min gradient run (flow rate-0.3 µl/min) using different combinations of buffer A (0.1% formic acid in LC–MS Grade water) and B (0.1% formic acid in ACN). The mass spectrometer was operated under Data Dependent Acquisition (DDA) mode.

### Data analysis

The raw data acquired from the Q-TOF Synapt G2 High Definition mass spectrometer was processed with MassLynx 4.1 (Waters Corp., MA, USA). The raw data files were converted to mzML format using the MS convert proteowizard tool. Spectra acquired from each of the technical replicates were submitted individually to MassLynx 4.1 for peak list generation. Protein identification and quantification were simultaneously performed using in-house Mascot 2.3.02 software (https://www.matrixscience.com/; Matrix Science, London, UK) by searching the individual peptide peak lists based on their m/z values against the UNIPROT database, specified for *Homo sapiens* taxonomy. The search parameters of maximum missed cleavage of one were allowed in the trypsin digests with a peptide mass tolerance of 2.85 Da and a fragment tolerance of 1.5 ppm. Carbamidomethyl (C), iTRAQ4plex (N-term), iTRAQ4plex (K) were chosen as fixed modifications and Oxidation (M) was set as variable modifications. Automatic decoy database search algorithm of in-house Mascot 2.3.02 software (https://www.matrixscience.com/) at a significance threshold P < 0.05 and default false discovery rate (FDR) settings at the peptide and protein level was used. Only peptide sequence matches (PSMs) with significant scores were used as evidence for proteins. The peptide and protein identification FDRs were controlled at ≤ 5% using the target decoy database. Minimum of one peptide was included for identification of each confident protein. Peak areas of the reporter ions from the isobaric tags were used to quantify relative abundance of proteins in the four experimental groups. The results were then exported into Microsoft Excel (Microsoft Corp., USA) for manual data interpretation.

### Statistical analysis

Relative fold changes for the three HLNRA groups (Label 115 for Group II, 116 for Group III and 117 for Group IV) were then calculated with respect to the NLNRA group (Label 114 for Group I). The statistical significance of the protein expression was calculated by Student's t-test (*P* ≤ 0.05) using the SciPy package of Python. For calculating p values, mean fold change from the two technical replicates was used as the missing third value. Normality assumption for the protein expression data set was checked using the Kolmogorov–Smirnov normality test. The calculated *P*-values were subsequently corrected for FDR in multiple testing experiments by Benjamini-Hochberg (BH) method^73^. Proteins with fold change ‘cut off’ iTRAQ ratio of ≤ 0.83 or ≥ 1.2 and with a BH adjusted *P* ≤ 0.1 were considered significantly modulated. Percentage coefficient of variation (% CV) was used as the method of tool to assess the experimental variation among technical replicates.

### Functional pathway analysis

The open source software DAVID (Database for Annotation, Visualization and Integrated Discovery) version 6.8 (http://david.abcc.ncifcrf.gov/) was used to identify enriched biological processes in HLNRA subjects compared to NLNRA individuals. The biological pathways modulated in HLNRA dose groups were predicted using KEGG module (Kyoto Encyclopedia of Genes and Genomes; https://www.kegg.jp/kegg/)^74^. The UniProt identification numbers of the altered proteins were used for the Gene Ontology Term (GOTERM) enrichment analysis by searching against the human proteome database as previously described^75,76^. This analysis identified over-representation of group of proteins common to biological processes, molecular function and biological pathways. The GOTERM process and pathways was considered significantly enriched when the enrichment *P*-value calculated by Fisher’s exact test was *P* ≤ 0.05.

### Construction of protein–protein interaction network

The interconnected PPI network was constructed from the differentially regulated proteins using the in-house Python-based pipeline (networkx v2.2 module) by searching against Human Protein Reference Database (HPRD), IntAct molecular interaction database (IntAct), Molecular INTeraction database (MINT), comprehensive resource of mammalian protein complexes (CORUM) and PhosphoSitePlus databases. The open source software Gephi ver. 0.9.2 was used to visualize the network and the giant connected components (GCCs) of the network were analyzed by various inbuilt Force Atlas2 force-field spatial layout algorithm. Significance of the GCC sizes was measured with an empirical z-score in each HLNRA dose group compared to random expectation. The network analysis provided the topological properties like Degree Centrality (DC), Betweenness Centrality (BC), Closeness Centrality (CC), and Clustering for each node. The nodes in the PPI networks represent proteins, while edges indicate the number of interactions between them. Nodes were colored based on the modulation pattern (up-regulated, down-regulated, or un-regulated) of the proteins. A cut-off of log2FC > 0.263 and log2FC < − 0.263 were used to identify the up-regulated and down-regulated hub proteins of the PPI network. Interactions were also colored based on physical interaction, protein complex and kinase-substrate relationship. The node size in the protein–protein interaction network was calculated using the equation, Node size = (DC + 1)*(abs|log_2_FC|+ 1), and it emphasizes differentially regulated proteins with high connectivity.

### RNA extraction and RT-qPCR analysis

For gene expression validation, blood samples were collected from an additional set of five individuals each from Group I (NLNRA), and Group II and III of HLNRA. Samples from Group IV of HLNRA could not be collected. All the samples were healthy males in the age group of 25–53 years (Supplementary Table S5). PBMCs were isolated, total RNA was extracted and was reverse transcribed to cDNA as detailed in Nishad and Ghosh^77^. Real-Time quantitative Polymerase Chain Reaction (RT-qPCR) was performed on LC480 Real-Time PCR machine (Roche Diagnostics, GmbH, Germany). The SYBR GREEN chemistry (Roche Diagnostics Pvt. Ltd., GmbH, Germany) based analysis with 12.5 µl reaction volume was used to study mRNA expression. The primer sets were designed using the Universal Probe Library Assay Design Center of Roche and are given in Supplementary Table S6. The cycling conditions consisted of a pre-incubation step at 95 °C for 5 min, followed by 40 cycles of denaturation at 95 °C (10 s), annealing at 60 °C (30 s) and extension at 72 °C (30 s). The C_t_ (cycle threshold) of the target gene was normalized to that of two reference genes (β-actin and GAPDH). As per MIQE guidelines^66^, stable expression of reference genes was first validated in our experimental conditions in three individuals randomly selected from the study samples. Melting curve analysis was performed for each primer set to confirm product-specific amplification. The relative change in gene expression was analyzed with the 2^−∆∆Ct^ method by Livak and Schmittgen^78^. Statistical analysis was performed using Student’s t-test. Data are represented as mean ± standard error of mean (SEM) from three replicates. P ≤ 0.05 was considered significant.

## Supplementary Information

Supplementary Information 1.

Supplementary Information 2a.

Supplementary Information 2b.

Supplementary Information 2c.

## Data Availability

The mass spectrometry proteomics data have been deposited to the ProteomeXchange Consortium via the PRIDE^79^ partner repository with the dataset identifier PXD022380.
