# Supplementary material for: Chronic exposure of humans to high level natural background radiation leads to robust expression of protective stress response proteins
Source: Sci Rep. 2021 Jan 19;11:1777. doi: 10.1038/s41598-020-80405-y (PMC7815775; doi:10.1038/s41598-020-80405-y)
Supplement: Supplementary file 1 — Supplementary Information 1. [file 41598_2020_80405_MOESM1_ESM.doc]

**Chronic exposure of humans to high level natural background radiation leads to robust expression of protective stress response proteins**

**S. Nishad^1,2^, Pankaj Kumar Chauhan^3^, R. Sowdhamini^3^ and Anu Ghosh^1, 2^,***

^1^Radiation Signaling Group, Radiation Biology & Health Sciences Division, Bio-Science Group, Bhabha Atomic Research Centre (BARC), Mumbai - 400 085, India

^2^Homi Bhabha National Institute (HBNI), Mumbai - 400 094, India

^3^Computational Approaches to Protein Science, National Centre for Biological Sciences (NCBS), Tata Institute for Fundamental Research (TIFR), Bangalore – 560 065, India.

Email: nishad@barc.gov.in (SN); pankajkc@ncbs.res.in (PKC); mini@ncbs.res.in (RS); anugh@barc.gov.in (AG)

**Corresponding author**

* Dr. Anu Ghosh

**Email:** [anugh@barc.gov.in](mailto:xxxxx@xxxx.xxx)

**(a)**

**Figure S1**

**Supplementary Figures**

**(b)**

**
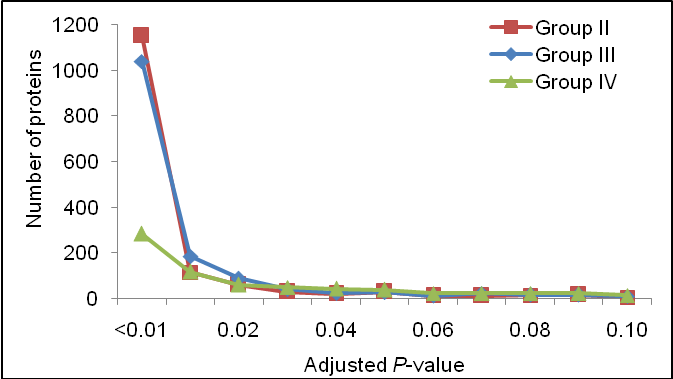

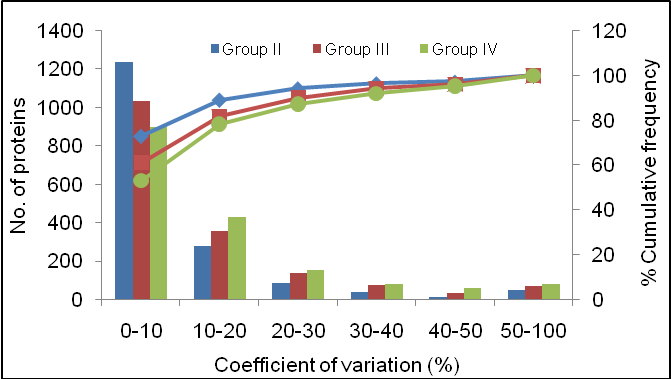
**

**(c)**

**(d)**

**
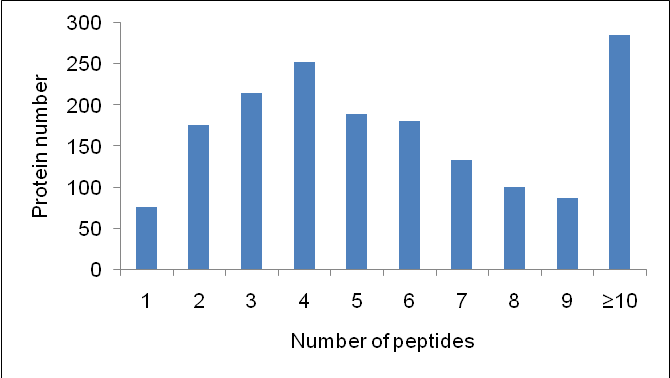

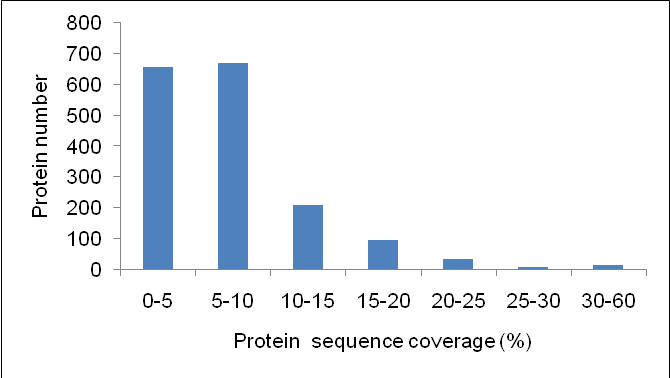
**

**Figure S1**. **Overview of iTRAQ analysis.** The distribution of (a) Adjusted *P*-value; (b) Coefficient of variation % (CV %); (c) Number of peptides, and (d) Protein sequence coverage (%) of all proteins modulated in the three HNLNA dose groups relative to NLNRA dose group.

**(a)**

**Figure S3**


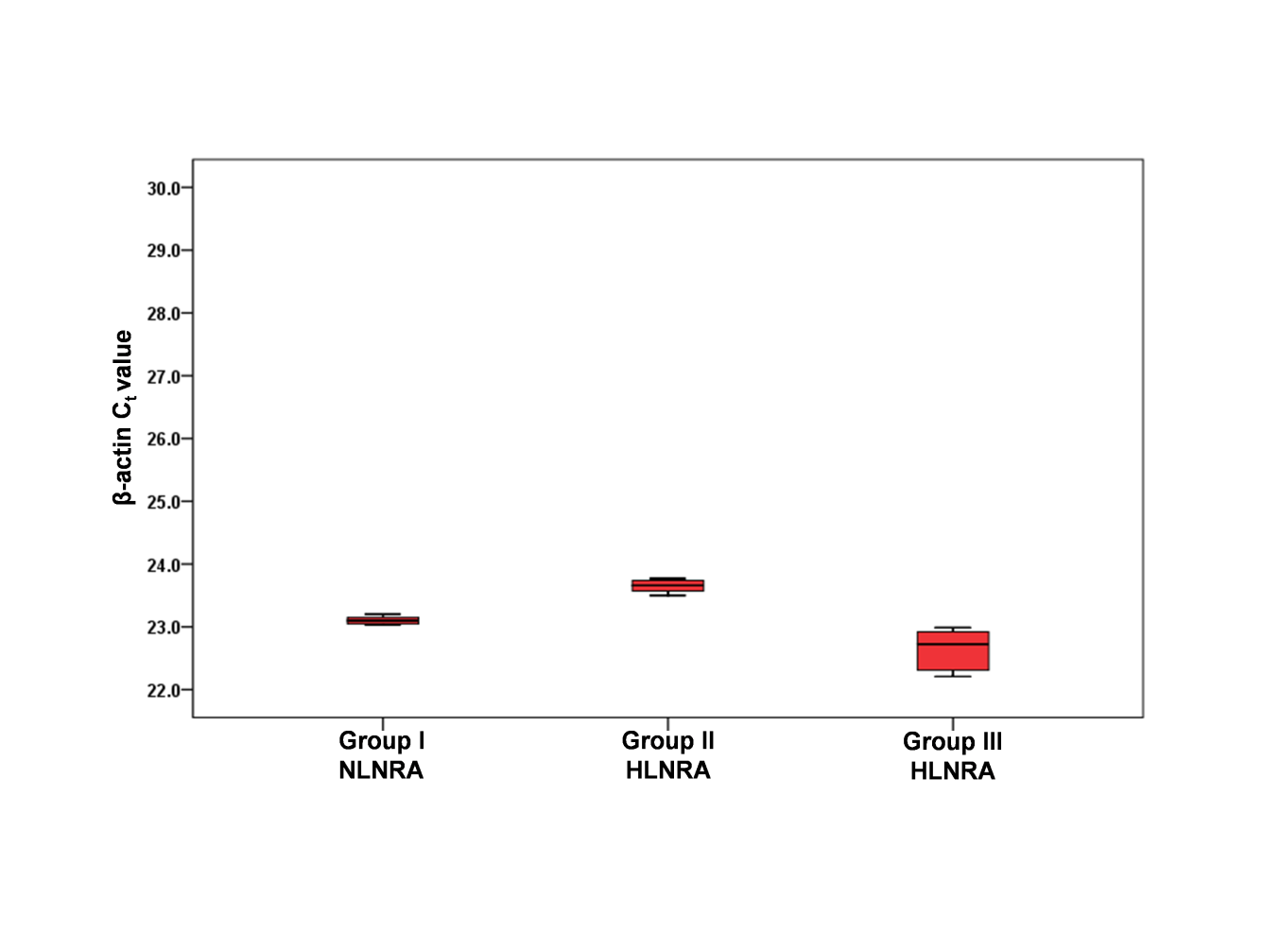


**(b)**


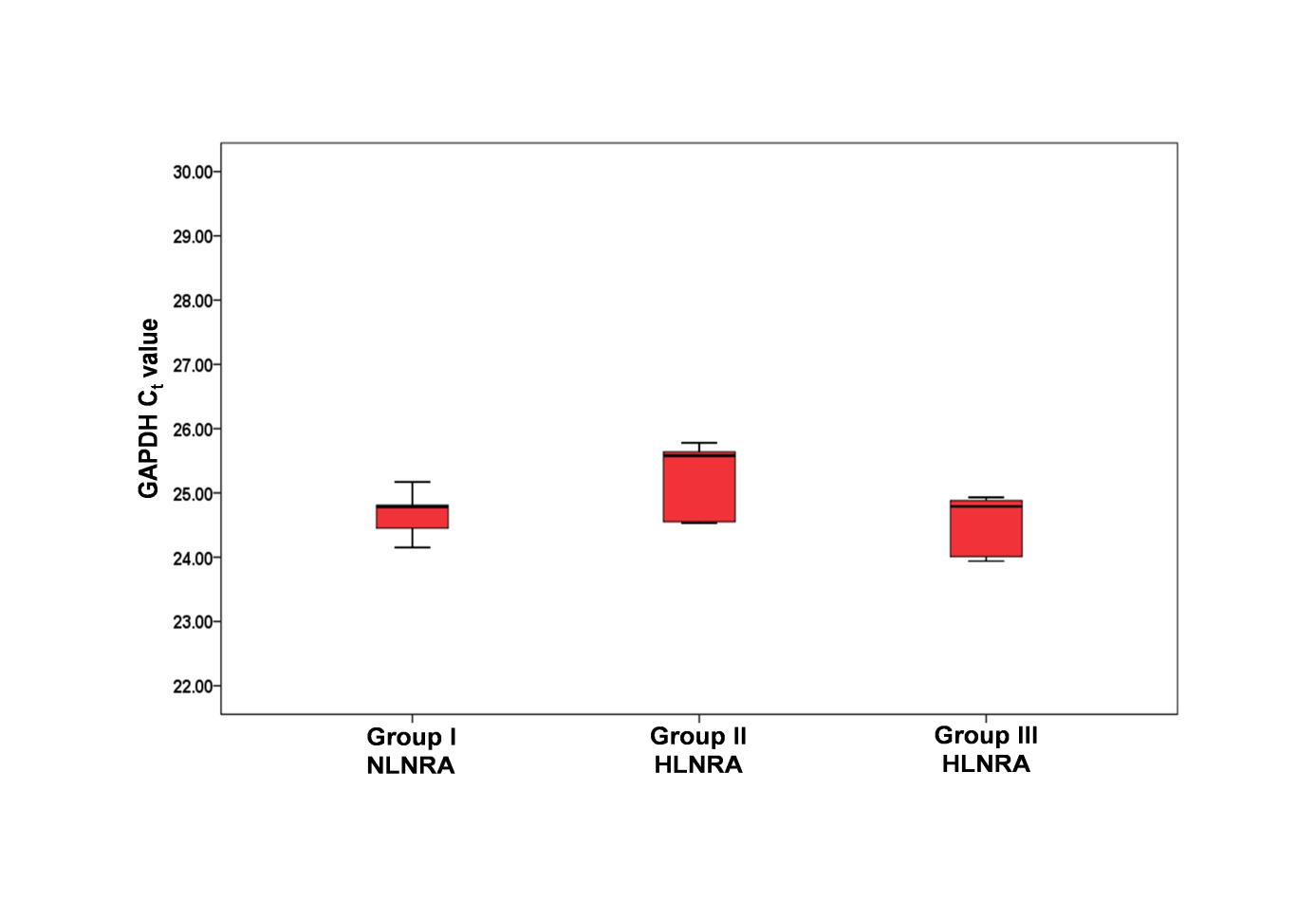


**Figure S3. Threshold cycle (C_t_) values for endogenous control genes in human PBMCs of NLNRA and HLNRA dose groups.** Each box represents C_t_ values of three individuals for (a) *β-actin* and, (b) *GAPDH.* The middle horizontal line is the median, the top and bottom of the boxes are the 25^th^ and 75^th^ percentiles, and the upper and lower horizontal lines (whiskers) indicate the ranges.

**Supplementary tables**

**Table S1: Characteristics of samples used for LC-MS/MS**

| **Area** | **Defined Dose Groups** | **No. of samples** | **Actual dose range**  **in mGy/y**  **(Mean dose ± SD)** | **Age**  **(mean ± SD) in years** |
| --- | --- | --- | --- | --- |
| **NLNRA**  (Control) | **Group I**  ≤1.50 mGy/y | 10 | 1.27 - 1.50  Mean dose: 1.38 ± 0.08 | 32.6 ± 2.55 |
| **HLNRA** | **Group II**  1.51- 5.0 mGy/y | 10 | 1.84 - 4.49  Mean dose: 3.07 ± 0.86 | 38.2 ± 7.77 |
|  | **Group III**  5.01- 14.0 mGy/y | 10 | 7.98 - 13.61  Mean dose: 10.50 ± 1.86 | 37.5 ± 6.88 |
|  | **Group IV**  ≥ 14.01 mGy/y | 10 | 14.69 - 20.25  Mean dose: 17.08 ± 1.75 | 42.6 ± 5.83 |

**Table S2: List of differentially regulated proteins in the three HLNRA dose groups relative to NLNRA dose group.** Mean changes in protein abundance for HLNRA individuals are represented as fold change (Mean FC). Differential proteins were chosen by an FC ratio of ≤0.83 or ≥1.2 with an adjusted *P*-value of ≤0.1 [Benjamini-Hochberg (BH) correction] in at least one dose group. UNIPROT accession number, protein description, peptide matches and protein sequence coverage (%) is listed for each protein.

| Accession No | Protein Description | Peptide Matches | Protein sequence coverage (%) | Group II | | Group III | | Group IV | |
| --- | --- | --- | --- | --- | --- | --- | --- | --- | --- |
|  |  |  |  | Mean FC | Adj. *P*- value | Mean FC | Adj. *P-* value | Mean FC | Adj. *P-* value |
| 1433Z_HUMAN | 14-3-3 protein zeta/delta | 12 | 20.8 | 1.46 | <0.001 | 1.71 | <0.001 | 1.32 | <0.001 |
| 2A5B_HUMAN | Serine/threonine-protein phosphatase 2A 56 kDa regulatory subunit beta isoform | 5 | 9.5 | 1.57 | <0.001 | 1.9 | <0.001 | 1.42 | <0.001 |
| 3BP1_HUMAN | SH3 domain-binding protein 1 | 12 | 21.7 | 1.46 | 0.005 | 1.73 | <0.001 | 1.29 | <0.001 |
| A1CF_HUMAN | APOBEC1 complementation factor | 7 | 15.8 | 1.78 | <0.001 | 2.58 | <0.001 | 1.97 | 0.001 |
| AAK1_HUMAN | AP2-associated protein kinase 1 | 4 | 5.3 | 1.76 | 0.009 | 2.13 | 0.003 | 1.8 | 0.046 |
| ACHA4_HUMAN | Neuronal acetylcholine receptor subunit alpha-4 | 3 | 6.1 | 1.4 | <0.001 | 1.59 | 0.003 | 1.26 | 0.002 |
| ACINU_HUMAN | Apoptotic chromatin condensation inducer in the nucleus | 8 | 3 | 1.43 | <0.001 | 1.74 | 0.001 | 1.33 | 0.008 |
| ACTB_HUMAN | Actin; cytoplasmic 1 | 36 | 35.2 | 1.51 | <0.001 | 1.25 | <0.001 | 0.8 | 0.001 |
| ACTN1_HUMAN | Alpha-actinin-1 | 11 | 14.3 | 1.48 | <0.001 | 1.7 | <0.001 | 1.21 | 0.012 |
| ADA22_HUMAN | Disintegrin and metalloproteinase domain-containing protein 22 | 9 | 6.7 | 1.42 | <0.001 | 1.69 | <0.001 | 1.22 | <0.001 |
| ADCK2_HUMAN | Uncharacterized aarF domain-containing protein kinase 2 | 2 | 5.1 | 1.58 | 0.051 | 2.37 | 0.046 | 2.15 | 0.213 |
| ADCY1_HUMAN | Adenylate cyclase type 1 | 6 | 8 | 1.45 | <0.001 | 1.73 | <0.001 | 1.31 | 0.001 |
| ADCY2_HUMAN | Adenylate cyclase type 2 | 3 | 5.6 | 2.39 | 0.086 | 2.91 | 0.109 | 2.9 | 0.225 |
| ADIPO_HUMAN | Adiponectin | 4 | 28.3 | 1.43 | <0.001 | 1.95 | 0.015 | 1.52 | 0.082 |
| AEBP2_HUMAN | Zinc finger protein AEBP2 | 3 | 14.1 | 1.55 | <0.001 | 1.64 | <0.001 | 1.12 | 0.023 |
| AGO3_HUMAN | Protein argonaute-3 | 6 | 6.7 | 1.42 | <0.001 | 1.59 | <0.001 | 1.18 | <0.001 |
| AGRB2_HUMAN | Adhesion G protein-coupled receptor B2 | 7 | 6.5 | 1.51 | 0.001 | 1.46 | 0.002 | 1.07 | 0.485 |
| AGRB3_HUMAN | Adhesion G protein-coupled receptor B3 | 4 | 4.4 | 1.25 | 0.272 | 1.58 | 0.006 | 1.27 | 0.140 |
| AHNK_HUMAN | Neuroblast differentiation-associated protein AHNAK | 17 | 3.1 | 1.35 | 0.002 | 1.5 | 0.006 | 1 | 0.977 |
| AKAP9_HUMAN | A-kinase anchor protein 9 | 13 | 4.6 | 1.52 | 0.001 | 1.78 | 0.001 | 1.31 | 0.043 |
| AKNA_HUMAN | AT-hook-containing transcription factor | 4 | 3.6 | 1.51 | 0.010 | 1.93 | <0.001 | 1.44 | 0.008 |
| ALDOA_HUMAN | Fructose-bisphosphate aldolase A | 5 | 12.9 | 1.74 | <0.001 | 2.12 | <0.001 | 1.59 | 0.006 |
| ALK_HUMAN | ALK tyrosine kinase receptor | 11 | 7.3 | 1.45 | <0.001 | 1.77 | <0.001 | 1.38 | 0.001 |
| ALPK3_HUMAN | Alpha-protein kinase 3 | 6 | 3.3 | 1.49 | <0.001 | 1.74 | <0.001 | 1.31 | <0.001 |
| AMOL2_HUMAN | Angiomotin-like protein 2 | 7 | 18 | 1.56 | <0.001 | 1.79 | <0.001 | 1.27 | 0.037 |
| ANK2_HUMAN | Ankyrin-2 | 14 | 5.1 | 1.49 | <0.001 | 1.75 | 0.001 | 1.38 | 0.015 |
| ANK3_HUMAN | Ankyrin-3 | 15 | 4.3 | 1.49 | <0.001 | 1.84 | <0.001 | 1.38 | 0.006 |
| ANR33_HUMAN | Ankyrin repeat domain-containing protein 33 | 5 | 26.5 | 1.48 | <0.001 | 1.58 | 0.057 | 1.11 | 0.622 |
| APBA1_HUMAN | Amyloid beta A4 precursor protein-binding family A member 1 | 4 | 7.9 | 1.34 | 0.028 | 1.4 | 0.033 | 1.13 | 0.001 |
| APC_HUMAN | Adenomatous polyposis coli protein | 19 | 9.7 | 1.4 | <0.001 | 1.44 | 0.022 | 1.04 | 0.808 |
| APC2_HUMAN | Adenomatous polyposis coli protein 2 | 4 | 6.9 | 1.46 | <0.001 | 1.63 | <0.001 | 1.22 | <0.001 |
| APEX2_HUMAN | DNA-(apurinic or apyrimidinic site) lyase 2 | 2 | 6 | 1.4 | 0.002 | 1.62 | <0.001 | 1.02 | 0.679 |
| APOE_HUMAN | Apolipoprotein E | 2 | 9.1 | 1.39 | 0.008 | 1.86 | <0.001 | 1.36 | 0.135 |
| AR6P4_HUMAN | ADP-ribosylation factor-like protein 6-interacting protein 4 | 7 | 10.9 | 1.56 | 0.001 | 1.24 | 0.451 | 1.05 | 0.706 |
| ARAP1_HUMAN | Arf-GAP with Rho-GAP domain; ANK repeat and PH domain-containing protein 1 | 5 | 3 | 1.5 | <0.001 | 1.7 | <0.001 | 1.26 | <0.001 |
| ARBK1_HUMAN | Beta-adrenergic receptor kinase 1 | 7 | 9.7 | 1.61 | <0.001 | 1.92 | <0.001 | 1.45 | <0.001 |
| ARHG2_HUMAN | Rho guanine nucleotide exchange factor 2 | 5 | 7.8 | 1.4 | 0.012 | 1.29 | 0.096 | 0.89 | 0.558 |
| ARHG5_HUMAN | Rho guanine nucleotide exchange factor 5 | 7 | 3.8 | 1.51 | 0.002 | 1.88 | <0.001 | 1.34 | <0.001 |
| ARHGI_HUMAN | Rho guanine nucleotide exchange factor 18 | 5 | 3.8 | 1.23 | 0.052 | 1.4 | 0.027 | 1.15 | <0.001 |
| ARI1A_HUMAN | AT-rich interactive domain-containing protein 1A | 26 | 17 | 1.23 | 0.189 | 1.49 | 0.001 | 1.08 | <0.001 |
| ARI1B_HUMAN | AT-rich interactive domain-containing protein 1B | 27 | 8.3 | 1.48 | <0.001 | 1.76 | <0.001 | 1.3 | 0.003 |
| ARI3A_HUMAN | AT-rich interactive domain-containing protein 3A | 6 | 16.9 | 1.56 | <0.001 | 1.74 | <0.001 | 1.25 | <0.001 |
| ARVC_HUMAN | Armadillo repeat protein deleted in velo-cardio-facial syndrome | 5 | 7.9 | 1.65 | 0.009 | 2.02 | <0.001 | 1.64 | 0.001 |
| ASB1_HUMAN | Ankyrin repeat and SOCS box protein 1 | 8 | 12.2 | 1.42 | 0.001 | 1.67 | 0.011 | 1.21 | 0.313 |
| ASH1L_HUMAN | Histone-lysine N-methyltransferase ASH1L | 8 | 2.8 | 1.27 | 0.157 | 1.61 | <0.001 | 1.15 | <0.001 |
| ASXL1_HUMAN | Putative Polycomb group protein ASXL1 | 9 | 9.5 | 1.44 | <0.001 | 1.6 | <0.001 | 1.17 | 0.163 |
| AT10A_HUMAN | Probable phospholipid-transporting ATPase VA | 8 | 5.9 | 1.42 | <0.001 | 1.56 | <0.001 | 1.12 | 0.008 |
| AT132_HUMAN | Probable cation-transporting ATPase 13A2 | 3 | 3.2 | 1.25 | 0.193 | 1.57 | <0.001 | 1.24 | 0.142 |
| ATAD5_HUMAN | ATPase family AAA domain-containing protein 5 | 11 | 5.4 | 1.46 | <0.001 | 1.66 | 0.001 | 1.22 | 0.071 |
| ATMIN_HUMAN | ATM interactor | 6 | 7.8 | 1.5 | <0.001 | 1.89 | <0.001 | 1.47 | 0.001 |
| ATP7A_HUMAN | Copper-transporting ATPase 1 | 6 | 4.1 | 1.5 | 0.001 | 1.81 | 0.007 | 1.34 | 0.103 |
| ATPB_HUMAN | ATP synthase subunit beta; mitochondrial | 5 | 8.5 | 1.45 | <0.001 | 1.56 | <0.001 | 1.18 | 0.004 |
| ATR_HUMAN | Serine/threonine-protein kinase ATR | 19 | 3.2 | 1.53 | <0.001 | 1.88 | <0.001 | 1.46 | <0.001 |
| ATRX_HUMAN | Transcriptional regulator ATRX | 7 | 3.9 | 1.53 | 0.003 | 1.73 | 0.009 | 1.29 | 0.099 |
| AURKB_HUMAN | Aurora kinase B | 4 | 7.8 | 1.45 | <0.001 | 1.77 | <0.001 | 1.39 | 0.002 |
| AXIN2_HUMAN | Axin-2 | 9 | 4.5 | 1.45 | <0.001 | 1.56 | <0.001 | 1.1 | 0.017 |
| BAG3_HUMAN | BAG family molecular chaperone regulator 3 | 9 | 14.1 | 1.39 | <0.001 | 1.8 | <0.001 | 1.44 | 0.049 |
| BAIP2_HUMAN | Brain-specific angiogenesis inhibitor 1-associated protein 2 | 5 | 13.6 | 1.3 | 0.106 | 1.69 | 0.001 | 1.55 | 0.076 |
| BC11A_HUMAN | B-cell lymphoma/leukemia 11A | 5 | 7.5 | 1.32 | <0.001 | 1.3 | 0.001 | 0.92 | 0.160 |
| BCAS3_HUMAN | Breast carcinoma-amplified sequence 3 | 4 | 8.3 | 1.68 | 0.006 | 2.18 | 0.017 | 1.59 | 0.034 |
| BCKD_HUMAN | [3-methyl-2-oxobutanoate dehydrogenase [lipoamide]] kinase; mitochondrial | 6 | 6.8 | 1.4 | <0.001 | 1.32 | 0.004 | 0.91 | 0.053 |
| BCL9_HUMAN | B-cell CLL/lymphoma 9 protein | 12 | 15.1 | 0.7 | 0.085 | 1.53 | 0.011 | 0.98 | 0.951 |
| BCL9L_HUMAN | B-cell CLL/lymphoma 9-like protein | 18 | 9.1 | 1.38 | 0.009 | 1.69 | <0.001 | 1.24 | <0.001 |
| BD1L1_HUMAN | Biorientation of chromosomes in cell division protein 1-like 1 | 8 | 3 | 1.39 | <0.001 | 1.67 | <0.001 | 1.24 | <0.001 |
| BIRC6_HUMAN | Baculoviral IAP repeat-containing protein 6 | 7 | 1.8 | 1.57 | <0.001 | 1.81 | 0.001 | 1.33 | 0.020 |
| BLM_HUMAN | Bloom syndrome protein | 7 | 3.7 | 2.37 | 0.063 | 3.83 | 0.058 | 4.12 | 0.148 |
| BORG5_HUMAN | Cdc42 effector protein 1 | 5 | 21.2 | 1.42 | <0.001 | 1.5 | <0.001 | 1.03 | 0.111 |
| BPTF_HUMAN | Nucleosome-remodeling factor subunit BPTF | 7 | 3.4 | 1.38 | <0.001 | 1.83 | <0.001 | 1.32 | 0.013 |
| BRSK1_HUMAN | Serine/threonine-protein kinase BRSK1 | 5 | 15 | 1.33 | 0.044 | 1.58 | 0.010 | 1.12 | 0.434 |
| BRSK2_HUMAN | Serine/threonine-protein kinase BRSK2 | 5 | 2.3 | 1.47 | 0.001 | 1.52 | 0.006 | 1.09 | 0.012 |
| BRWD1_HUMAN | Bromodomain and WD repeat-containing protein 1 | 11 | 4.4 | 1.46 | <0.001 | 1.74 | <0.001 | 1.3 | <0.001 |
| BRWD3_HUMAN | Bromodomain and WD repeat-containing protein 3 | 4 | 3.5 | 1.33 | 0.023 | 1.77 | <0.001 | 1.32 | <0.001 |
| BTBD6_HUMAN | BTB/POZ domain-containing protein 6 | 3 | 8.9 | 1.07 | 0.834 | 1.55 | 0.011 | 1.85 | 0.122 |
| BZW2_HUMAN | Basic leucine zipper and W2 domain-containing protein 2 | 1 | 4.5 | 1.5 | <0.001 | 1.83 | 0.024 | 1.37 | 0.231 |
| CAB39_HUMAN | Calcium-binding protein 39 | 5 | 13.5 | 1.43 | <0.001 | 1.72 | <0.001 | 1.23 | 0.001 |
| CAF1B_HUMAN | Chromatin assembly factor 1 subunit B | 5 | 7 | 1.37 | <0.001 | 1.53 | 0.001 | 1.12 | 0.005 |
| CALM_HUMAN | Calmodulin OS=Homo sapiens GN=CALM1 PE=1 SV=2 | 12 | 33.6 | 0.74 | 0.008 | 0.87 | 0.742 | 0.7 | 0.420 |
| CALR_HUMAN | Calreticulin | 4 | 12.9 | 1.48 | <0.001 | 1.62 | 0.002 | 1.18 | 0.181 |
| CAMP3_HUMAN | Calmodulin-regulated spectrin-associated protein 3 | 9 | 4.7 | 1.42 | <0.001 | 1.51 | 0.008 | 1.02 | 0.808 |
| CAN2_HUMAN | Calpain-2 catalytic subunit | 4 | 3.7 | 1.5 | <0.001 | 1.63 | <0.001 | 1.27 | 0.047 |
| CAN3_HUMAN | Calpain-3 | 2 | 3 | 1.5 | 0.002 | 1.75 | 0.001 | 1.24 | <0.001 |
| CASC3_HUMAN | Protein CASC3 | 7 | 10.5 | 1.17 | 0.296 | 1.58 | 0.015 | 1.21 | 0.063 |
| CATIN_HUMAN | Cactin | 3 | 5.3 | 1.61 | <0.001 | 1.82 | <0.001 | 1.42 | <0.001 |
| CBX6_HUMAN | Chromobox protein homolog 6 | 4 | 10 | 1.35 | 0.008 | 1.61 | 0.083 | 1.13 | 0.681 |
| CC14A_HUMAN | Dual specificity protein phosphatase CDC14A | 7 | 17.3 | 1.45 | <0.001 | 1.86 | <0.001 | 1.74 | 0.021 |
| CD19_HUMAN | B-lymphocyte antigen CD19 | 2 | 2.9 | 1.26 | 0.111 | 1.39 | 0.041 | 1.02 | 0.798 |
| CD2B2_HUMAN | CD2 antigen cytoplasmic tail-binding protein 2 | 2 | 2.3 | 1.34 | 0.005 | 1.8 | <0.001 | 1.55 | 0.051 |
| CD44_HUMAN | CD44 antigen | 1 | 1.3 | 0.83 | 0.515 | 1.21 | 0.072 | 0.98 | 0.903 |
| CDC5L_HUMAN | Cell division cycle 5-like protein | 5 | 6.7 | 1.36 | <0.001 | 1.58 | <0.001 | 1.17 | 0.002 |
| CDHR5_HUMAN | Cadherin-related family member 5 | 4 | 5.2 | 1.4 | <0.001 | 1.7 | <0.001 | 1.26 | 0.002 |
| CDK12_HUMAN | Cyclin-dependent kinase 12 | 4 | 3.2 | 1.41 | 0.010 | 1.56 | 0.009 | 1.15 | 0.118 |
| CDKL5_HUMAN | Cyclin-dependent kinase-like 5 | 4 | 5 | 1.16 | 0.366 | 1.62 | 0.004 | 0.89 | 0.356 |
| CDN1A_HUMAN | Cyclin-dependent kinase inhibitor 1 | 1 | 17.7 | 1.31 | 0.003 | 1.63 | <0.001 | 1.22 | <0.001 |
| CDSN_HUMAN | Corneodesmosin | 5 | 7.9 | 1.47 | 0.036 | 1.34 | 0.016 | 1.11 | 0.658 |
| CECR2_HUMAN | Cat eye syndrome critical region protein 2 | 11 | 6.9 | 1.42 | 0.001 | 1.71 | 0.001 | 1.28 | 0.012 |
| CELR1_HUMAN | Cadherin EGF LAG seven-pass G-type receptor 1 | 8 | 3.7 | 1.45 | <0.001 | 1.77 | 0.004 | 1.33 | 0.015 |
| CHD7_HUMAN | Chromodomain-helicase-DNA-binding protein 7 | 9 | 5 | 1.42 | 0.001 | 1.64 | 0.024 | 1.17 | 0.320 |
| CHD8_HUMAN | Chromodomain-helicase-DNA-binding protein 8 | 5 | 3.4 | 1.35 | 0.093 | 1.79 | 0.003 | 1.39 | 0.034 |
| CHK1_HUMAN | Serine/threonine-protein kinase Chk1 | 3 | 12.8 | 1.32 | 0.054 | 1.48 | 0.010 | 1.14 | 0.141 |
| CHP2_HUMAN | Calcineurin B homologous protein 2 | 2 | 17.9 | 1.74 | 0.005 | 2.31 | 0.007 | 1.88 | 0.058 |
| CHST4_HUMAN | Carbohydrate sulfotransferase 4 | 1 | 3.6 | 1.2 | 0.489 | 1.64 | 0.001 | 1.47 | 0.009 |
| CLAP1_HUMAN | CLIP-associating protein 1 | 15 | 8.1 | 1.44 | 0.010 | 1.79 | <0.001 | 1.34 | 0.012 |
| CLAP2_HUMAN | CLIP-associating protein 2 | 15 | 14.6 | 1.4 | <0.001 | 1.62 | <0.001 | 1.15 | 0.008 |
| CLK3_HUMAN | Dual specificity protein kinase CLK3 | 5 | 2.8 | 1.37 | 0.057 | 1.55 | 0.028 | 1.14 | 0.325 |
| CNOT1_HUMAN | CCR4-NOT transcription complex subunit 1 | 12 | 2 | 1.73 | <0.001 | 2.08 | 0.001 | 1.68 | 0.014 |
| CNT3B_HUMAN | Contactin-associated protein-like 3B | 3 | 3.3 | 0.95 | 0.780 | 1.09 | 0.308 | 0.58 | 0.080 |
| CNTN5_HUMAN | Contactin-5 | 3 | 4.6 | 1.75 | 0.024 | 1.75 | 0.018 | 1.29 | 0.260 |
| CNTN6_HUMAN | Contactin-6 | 3 | 3.8 | 1.38 | <0.001 | 1.52 | 0.001 | 1.11 | 0.395 |
| CNTP3_HUMAN | Contactin-associated protein-like 3 | 7 | 6 | 1.48 | <0.001 | 1.74 | 0.001 | 1.27 | 0.006 |
| CO1A1_HUMAN | Collagen alpha-1(I) chain | 15 | 18.8 | 1.38 | <0.001 | 1.49 | 0.001 | 1.13 | 0.050 |
| CO1A2_HUMAN | Collagen alpha-2(I) chain | 23 | 30.8 | 1.41 | <0.001 | 1.59 | <0.001 | 1.18 | 0.013 |
| CO3A1_HUMAN | Collagen alpha-1(III) chain | 13 | 21.7 | 1.84 | 0.001 | 2.06 | <0.001 | 1.73 | 0.002 |
| CO4A1_HUMAN | Collagen alpha-1(IV) chain | 6 | 6.5 | 1.38 | 0.001 | 1.38 | 0.021 | 0.86 | 0.413 |
| CO4A2_HUMAN | Collagen alpha-2(IV) chain | 21 | 15.7 | 1.37 | <0.001 | 1.77 | 0.002 | 1.27 | 0.008 |
| CO4A3_HUMAN | Collagen alpha-3(IV) chain | 18 | 15 | 1.44 | <0.001 | 1.65 | <0.001 | 1.22 | 0.014 |
| CO4A4_HUMAN | Collagen alpha-4(IV) chain | 21 | 21.6 | 1.68 | 0.001 | 1.76 | 0.002 | 1.35 | 0.126 |
| CO4A5_HUMAN | Collagen alpha-5(IV) chain | 16 | 13 | 1.28 | 0.039 | 1.2 | 0.280 | 0.87 | 0.085 |
| CO4A6_HUMAN | Collagen alpha-6(IV) chain | 16 | 14.8 | 1.32 | 0.089 | 1.72 | <0.001 | 1.33 | <0.001 |
| CO5A1_HUMAN | Collagen alpha-1(V) chain | 9 | 9.4 | 1.39 | <0.001 | 1.57 | 0.001 | 1.13 | <0.001 |
| CO5A2_HUMAN | Collagen alpha-2(V) chain | 18 | 18.3 | 1.18 | 0.028 | 1.54 | <0.001 | 1.14 | 0.088 |
| CO5A3_HUMAN | Collagen alpha-3(V) chain | 14 | 12.3 | 1.45 | <0.001 | 1.6 | <0.001 | 1.16 | 0.006 |
| CO6A2_HUMAN | Collagen alpha-2(VI) chain | 5 | 7.1 | 1.63 | <0.001 | 2.01 | 0.001 | 1.57 | 0.004 |
| CO6A3_HUMAN | Collagen alpha-3(VI) chain | 8 | 3.2 | 1.4 | <0.001 | 1.5 | <0.001 | 1.06 | 0.186 |
| CO6A5_HUMAN | Collagen alpha-5(VI) chain | 9 | 4.8 | 1.5 | <0.001 | 1.7 | <0.001 | 1.31 | <0.001 |
| CO6A6_HUMAN | Collagen alpha-6(VI) chain | 9 | 5.6 | 1.46 | <0.001 | 1.59 | <0.001 | 1.19 | 0.001 |
| CO7A1_HUMAN | Collagen alpha-1(VII) chain | 28 | 13.8 | 1.5 | <0.001 | 1.69 | <0.001 | 1.23 | 0.003 |
| CO8A2_HUMAN | Collagen alpha-2(VIII) chain | 5 | 9.2 | 1.05 | 0.832 | 1.8 | 0.023 | 1.55 | 0.009 |
| CO9A1_HUMAN | Collagen alpha-1(IX) chain | 12 | 24.1 | 1.42 | 0.001 | 1.7 | 0.004 | 1.18 | 0.245 |
| CO9A2_HUMAN | Collagen alpha-2(IX) chain | 7 | 25 | 1.43 | <0.001 | 1.64 | <0.001 | 1.26 | <0.001 |
| COBA1_HUMAN | Collagen alpha-1(XI) chain | 15 | 15.4 | 1.46 | 0.001 | 1.51 | <0.001 | 1.12 | <0.001 |
| COBL1_HUMAN | Cordon-bleu protein-like 1 | 9 | 6.9 | 1.19 | 0.341 | 1.64 | 0.015 | 1.26 | 0.110 |
| COCA1_HUMAN | Collagen alpha-1(XII) chain | 6 | 2.3 | 1.43 | <0.001 | 1.48 | <0.001 | 1.14 | 0.003 |
| COE4_HUMAN | Transcription factor COE4 | 8 | 7.5 | 1.59 | <0.001 | 1.71 | <0.001 | 1.3 | 0.006 |
| COEA1_HUMAN | Collagen alpha-1(XIV) chain | 6 | 3.3 | 1.33 | 0.026 | 1.35 | 0.094 | 0.95 | 0.764 |
| COFA1_HUMAN | Collagen alpha-1(XV) chain | 5 | 3.3 | 1.31 | 0.144 | 1.56 | 0.023 | 1.15 | 0.338 |
| COGA1_HUMAN | Collagen alpha-1(XVI) chain | 15 | 8.7 | 1.47 | <0.001 | 1.79 | <0.001 | 1.37 | <0.001 |
| COHA1_HUMAN | Collagen alpha-1(XVII) chain | 11 | 10.6 | 1.41 | <0.001 | 1.79 | 0.003 | 1.35 | 0.008 |
| COIA1_HUMAN | Collagen alpha-1(XVIII) chain | 12 | 10.5 | 1.45 | <0.001 | 1.66 | <0.001 | 1.18 | 0.002 |
| COJA1_HUMAN | Collagen alpha-1(XIX) chain | 6 | 7.7 | 1.32 | <0.001 | 1.61 | <0.001 | 1.21 | 0.003 |
| CORA1_HUMAN | Collagen alpha-1(XXVII) chain | 26 | 18 | 1.44 | <0.001 | 1.68 | <0.001 | 1.26 | <0.001 |
| COSA1_HUMAN | Collagen alpha-1(XXVIII) chain | 9 | 8.3 | 1.44 | <0.001 | 1.42 | 0.040 | 0.99 | 0.951 |
| CSN5_HUMAN | COP9 signalosome complex subunit 5 | 3 | 7.5 | 1.53 | <0.001 | 1.6 | <0.001 | 1.2 | 0.005 |
| CTND1_HUMAN | Catenin delta-1 | 6 | 12.7 | 1.41 | <0.001 | 1.62 | 0.001 | 1.16 | 0.038 |
| CTND2_HUMAN | Catenin delta-2 | 11 | 5.9 | 1.51 | 0.001 | 1.55 | <0.001 | 1.06 | 0.243 |
| CTTB2_HUMAN | Cortactin-binding protein 2 | 5 | 2.9 | 1.32 | 0.003 | 1.43 | 0.068 | 0.99 | 0.939 |
| CUL2_HUMAN | Cullin-2 | 4 | 7.7 | 1.49 | <0.001 | 1.65 | <0.001 | 1.17 | 0.072 |
| CUL4A_HUMAN | Cullin-4A | 7 | 5.7 | 1.49 | <0.001 | 1.73 | <0.001 | 1.25 | 0.004 |
| CUL9_HUMAN | Cullin-9 | 11 | 6.9 | 1.44 | <0.001 | 1.86 | 0.002 | 1.36 | 0.017 |
| CUX1_HUMAN | Homeobox protein cut-like 1 | 9 | 6 | 1.41 | 0.003 | 1.71 | <0.001 | 1.27 | 0.002 |
| CYC_HUMAN | Cytochrome c | 2 | 14.3 | 1.46 | <0.001 | 1.76 | <0.001 | 1.28 | 0.002 |
| CYTSA_HUMAN | Cytospin-A | 10 | 11.6 | 1.38 | <0.001 | 1.43 | 0.003 | 1.02 | 0.841 |
| DAB2P_HUMAN | Disabled homolog 2-interacting protein | 5 | 5.2 | 1.44 | 0.001 | 1.27 | 0.393 | 1.08 | 0.590 |
| DACT3_HUMAN | Dapper homolog 3 | 4 | 5.6 | 1.61 | 0.003 | 2 | 0.006 | 1.57 | 0.061 |
| DAPK1_HUMAN | Death-associated protein kinase 1 | 7 | 3.4 | 1.41 | <0.001 | 1.56 | 0.001 | 1.11 | 0.259 |
| DCLK1_HUMAN | Serine/threonine-protein kinase DCLK1 | 6 | 8.2 | 1.48 | <0.001 | 1.61 | 0.001 | 1.15 | 0.075 |
| DDX17_HUMAN | Probable ATP-dependent RNA helicase DDX17 | 10 | 2.3 | 1.36 | 0.002 | 1.71 | 0.001 | 1.24 | 0.016 |
| DDX47_HUMAN | Probable ATP-dependent RNA helicase DDX47 | 2 | 6.4 | 1.63 | <0.001 | 1.89 | <0.001 | 1.37 | 0.005 |
| DEN2A_HUMAN | DENN domain-containing protein 2A | 4 | 5.6 | 1.21 | 0.020 | 1.27 | 0.002 | 0.91 | 0.364 |
| DEN5A_HUMAN | DENN domain-containing protein 5A | 10 | 2.8 | 1.45 | <0.001 | 1.73 | <0.001 | 1.3 | <0.001 |
| DESP_HUMAN | Desmoplakin | 8 | 2.9 | 1.24 | 0.086 | 1.25 | 0.001 | 0.92 | 0.269 |
| DHX29_HUMAN | ATP-dependent RNA helicase DHX29 | 4 | 4.6 | 1.57 | 0.008 | 1.61 | 0.001 | 1.16 | 0.002 |
| DHX33_HUMAN | Putative ATP-dependent RNA helicase DHX33 | 7 | 5.5 | 1.17 | 0.006 | 1.5 | <0.001 | 1.18 | 0.098 |
| DHX36_HUMAN | ATP-dependent RNA helicase DHX36 | 10 | 5 | 1.45 | <0.001 | 1.91 | <0.001 | 1.53 | 0.005 |
| DHX57_HUMAN | Putative ATP-dependent RNA helicase DHX57 | 15 | 4.3 | 1.46 | <0.001 | 1.69 | 0.002 | 1.26 | 0.067 |
| DIAP2_HUMAN | Protein diaphanous homolog 2 | 13 | 5.1 | 1.47 | <0.001 | 1.69 | <0.001 | 1.26 | <0.001 |
| DISC1_HUMAN | Disrupted in schizophrenia 1 protein | 5 | 4.8 | 1.55 | <0.001 | 1.42 | 0.076 | 1.05 | 0.653 |
| DLG5_HUMAN | Disks large homolog 5 | 8 | 6.1 | 1.38 | 0.012 | 1.72 | <0.001 | 1.29 | 0.005 |
| DLX1_HUMAN | Homeobox protein DLX-1 | 8 | 17.6 | 1.42 | 0.001 | 1.71 | 0.003 | 1.22 | 0.174 |
| DMRT2_HUMAN | Doublesex- and mab-3-related transcription factor 2 | 4 | 7.8 | 1.4 | <0.001 | 1.7 | <0.001 | 1.3 | <0.001 |
| DOC11_HUMAN | Dedicator of cytokinesis protein 11 | 10 | 4.1 | 1.48 | <0.001 | 1.69 | <0.001 | 1.25 | <0.001 |
| DOCK3_HUMAN | Dedicator of cytokinesis protein 3 | 5 | 3 | 1.3 | <0.001 | 1.73 | 0.003 | 1.18 | 0.102 |
| DOCK6_HUMAN | Dedicator of cytokinesis protein 6 | 9 | 7.3 | 1.36 | <0.001 | 1.64 | 0.001 | 1.23 | 0.068 |
| DOCK7_HUMAN | Dedicator of cytokinesis protein 7 | 11 | 6.2 | 1.43 | <0.001 | 1.87 | 0.001 | 1.4 | 0.019 |
| DOCK9_HUMAN | Dedicator of cytokinesis protein 9 | 6 | 2.9 | 1.45 | <0.001 | 1.69 | 0.012 | 1.23 | 0.304 |
| DPOLQ_HUMAN | DNA polymerase theta | 6 | 3.1 | 1.35 | 0.006 | 1.56 | 0.002 | 1.16 | 0.096 |
| DPTOR_HUMAN | DEP domain-containing mTOR-interacting protein | 5 | 18.1 | 0.93 | 0.158 | 1.52 | 0.090 | 1.21 | 0.516 |
| DSCL1_HUMAN | Down syndrome cell adhesion molecule-like protein 1 | 9 | 4.1 | 1.53 | 0.001 | 1.64 | <0.001 | 1.21 | 0.005 |
| DSG2_HUMAN | Desmoglein-2 | 9 | 9.9 | 1.47 | <0.001 | 1.69 | <0.001 | 1.26 | <0.001 |
| DSRAD_HUMAN | Double-stranded RNA-specific adenosine deaminase | 7 | 5.5 | 1.64 | 0.012 | 1.88 | 0.023 | 1.42 | 0.126 |
| DUS4_HUMAN | Dual specificity protein phosphatase 4 | 3 | 6.3 | 1.47 | <0.001 | 1.57 | 0.027 | 1.15 | 0.500 |
| DUS7_HUMAN | Dual specificity protein phosphatase 7 | 7 | 6.9 | 1.56 | 0.001 | 1.66 | 0.001 | 1.51 | 0.061 |
| DVL2_HUMAN | Segment polarity protein dishevelled homolog DVL-2 | 3 | 6.9 | 1.5 | 0.006 | 1.89 | 0.011 | 1.47 | 0.084 |
| DVL3_HUMAN | Segment polarity protein dishevelled homolog DVL-3 | 4 | 6.8 | 1.44 | <0.001 | 1.55 | 0.009 | 1.13 | 0.279 |
| DYRK2_HUMAN | Dual specificity tyrosine-phosphorylation-regulated kinase 2 | 3 | 4.8 | 1.38 | 0.002 | 1.42 | 0.004 | 1.06 | 0.492 |
| DYST_HUMAN | Dystonin | 12 | 2.3 | 1.46 | <0.001 | 1.58 | <0.001 | 1.16 | 0.015 |
| E2AK3_HUMAN | Eukaryotic translation initiation factor 2-alpha kinase 3 | 5 | 4.8 | 1.42 | <0.001 | 1.48 | 0.005 | 0.95 | 0.185 |
| E2F1_HUMAN | Transcription factor E2F1 | 3 | 5.9 | 1.43 | <0.001 | 1.55 | 0.002 | 1.09 | 0.192 |
| E2F8_HUMAN | Transcription factor E2F8 | 3 | 3.9 | 1.5 | <0.001 | 1.78 | <0.001 | 1.34 | <0.001 |
| E41L1_HUMAN | Band 4.1-like protein 1 | 5 | 5.3 | 1.41 | 0.001 | 1.75 | <0.001 | 1.36 | <0.001 |
| EEPD1_HUMAN | Endonuclease/exonuclease/phosphatase family domain-containing protein 1 | 7 | 12.7 | 1.52 | <0.001 | 1.88 | <0.001 | 1.41 | 0.004 |
| EGLN1_HUMAN | Egl nine homolog 1 | 7 | 11.5 | 1.44 | <0.001 | 1.75 | <0.001 | 1.25 | 0.019 |
| EID2_HUMAN | EP300-interacting inhibitor of differentiation 2 | 7 | 30.5 | 1.4 | <0.001 | 1.68 | 0.001 | 1.26 | 0.050 |
| ELN_HUMAN | Elastin | 6 | 10.2 | 1.48 | <0.001 | 1.67 | <0.001 | 1.22 | <0.001 |
| EMAL3_HUMAN | Echinoderm microtubule-associated protein-like 3 | 6 | 5.8 | 1.55 | <0.001 | 1.88 | <0.001 | 1.21 | 0.170 |
| EMAL4_HUMAN | Echinoderm microtubule-associated protein-like 4 | 4 | 6.6 | 1.53 | <0.001 | 1.83 | <0.001 | 1.34 | 0.011 |
| EMIL1_HUMAN | EMILIN-1 | 5 | 9.8 | 1.27 | 0.124 | 1.69 | <0.001 | 1.22 | 0.001 |
| EMIL2_HUMAN | EMILIN-2 | 6 | 8.5 | 1.56 | <0.001 | 1.7 | <0.001 | 1.24 | 0.051 |
| EMSY_HUMAN | BRCA2-interacting transcriptional repressor EMSY | 4 | 3.6 | 1.38 | <0.001 | 1.74 | <0.001 | 1.27 | <0.001 |
| ENOA_HUMAN | Alpha-enolase | 8 | 13.1 | 1.42 | 0.001 | 1.52 | <0.001 | 1.11 | 0.081 |
| EP2A2_HUMAN | Laforin; isoform 9 | 5 | 22.7 | 1.42 | <0.001 | 1.61 | <0.001 | 1.07 | 0.486 |
| EPHA4_HUMAN | Ephrin type-A receptor 4 | 8 | 7.9 | 1.53 | <0.001 | 1.77 | <0.001 | 1.35 | 0.004 |
| EPHA8_HUMAN | Ephrin type-A receptor | 6 | 4.9 | 1.44 | <0.001 | 1.46 | 0.002 | 1.22 | 0.021 |
| EPIPL_HUMAN | Epiplakin | 8 | 1.3 | 1.48 | <0.001 | 1.63 | <0.001 | 1.19 | 0.017 |
| ERBB4_HUMAN | Receptor tyrosine-protein kinase erbB-4 | 5 | 7 | 1.3 | 0.053 | 1.5 | 0.002 | 1.05 | 0.131 |
| ESYT2_HUMAN | Extended synaptotagmin-2 | 3 | 3.7 | 1.36 | 0.001 | 1.57 | <0.001 | 1.24 | 0.201 |
| EXOC8_HUMAN | Exocyst complex component 8 | 2 | 3.6 | 1.43 | 0.001 | 1.67 | <0.001 | 1.36 | 0.008 |
| EXOSX_HUMAN | Exosome component 10 | 3 | 4.1 | 1.46 | <0.001 | 1.72 | <0.001 | 1.29 | 0.004 |
| FANCA_HUMAN | Fanconianemia group A protein | 2 | 3.2 | 1.29 | 0.006 | 1.66 | 0.010 | 1.23 | 0.136 |
| FANCI_HUMAN | Fanconianemia group I protein | 8 | 3.4 | 1.57 | 0.001 | 1.75 | 0.001 | 1.42 | 0.006 |
| FAT1_HUMAN | Protocadherin Fat 1 | 7 | 1.9 | 1.43 | <0.001 | 1.56 | <0.001 | 1.15 | 0.021 |
| FAT4_HUMAN | Protocadherin Fat 4 | 7 | 1.9 | 1.42 | <0.001 | 1.55 | <0.001 | 1.21 | 0.018 |
| FBSP1_HUMAN | F-box/SPRY domain-containing protein 1 | 10 | 13.3 | 1.57 | <0.001 | 1.66 | <0.001 | 1.19 | 0.009 |
| FBX10_HUMAN | F-box only protein 10 | 5 | 7 | 1.64 | 0.001 | 2.06 | <0.001 | 1.79 | 0.002 |
| FGD3_HUMAN | FYVE; RhoGEF and PH domain-containing protein | 3 | 5.1 | 1.48 | 0.011 | 1.73 | 0.002 | 1.38 | <0.001 |
| FGD6_HUMAN | FYVE; RhoGEF and PH domain-containing protein | 3 | 1.5 | 1.41 | <0.001 | 1.79 | <0.001 | 1.3 | <0.001 |
| FIBA_HUMAN | Fibrinogen alpha chain | 9 | 7.7 | 1.37 | <0.001 | 1.4 | 0.011 | 1.05 | 0.451 |
| FIBB_HUMAN | Fibrinogen beta chain | 4 | 7.3 | 1.32 | 0.001 | 1.32 | 0.002 | 0.89 | 0.021 |
| FIBG_HUMAN | Fibrinogen gamma chain | 3 | 2.9 | 1.53 | <0.001 | 1.78 | <0.001 | 1.42 | 0.002 |
| FIG4_HUMAN | Polyphosphoinositide phosphatase | 6 | 6 | 1.32 | <0.001 | 1.73 | 0.002 | 1.3 | 0.058 |
| FLNA_HUMAN | Filamin-A | 28 | 9.8 | 1.46 | <0.001 | 1.65 | <0.001 | 1.22 | 0.005 |
| FLNB_HUMAN | Filamin-B | 10 | 4 | 1.43 | 0.001 | 1.88 | <0.001 | 1.3 | 0.002 |
| FLOT2_HUMAN | Flotillin-2 | 4 | 7.2 | 1.55 | 0.004 | 1.73 | 0.012 | 1.27 | 0.117 |
| FMN1_HUMAN | Formin-1 | 13 | 10.3 | 1.47 | 0.003 | 1.71 | 0.003 | 1.29 | 0.016 |
| FMNL1_HUMAN | Formin-like protein 1 | 6 | 4.3 | 1.54 | <0.001 | 1.84 | <0.001 | 1.39 | <0.001 |
| FNIP1_HUMAN | Folliculin-interacting protein 1 | 5 | 5.7 | 1.4 | <0.001 | 1.68 | <0.001 | 1.19 | 0.071 |
| FOG1_HUMAN | Zinc finger protein ZFPM1 | 5 | 8.1 | 1.32 | 0.001 | 1.41 | 0.018 | 0.9 | 0.239 |
| FOG2_HUMAN | Zinc finger protein ZFPM2 | 3 | 3.3 | 1.38 | 0.008 | 1.42 | 0.029 | 1.12 | 0.433 |
| FREM2_HUMAN | FRAS1-related extracellular matrix protein 2 | 8 | 3.6 | 1.45 | <0.001 | 1.76 | 0.001 | 1.27 | 0.027 |
| FTCD_HUMAN | Formimidoyltransferase-cyclodeaminase | 14 | 9.4 | 1.4 | <0.001 | 1.68 | 0.001 | 1.26 | 0.038 |
| FUBP2_HUMAN | Far upstream element-binding protein 2 | 9 | 12.7 | 1.35 | 0.005 | 1.85 | <0.001 | 1.59 | 0.018 |
| FURIN_HUMAN | Furin | 4 | 7.3 | 1.58 | <0.001 | 2.21 | 0.027 | 1.82 | 0.127 |
| FUS_HUMAN | RNA-binding protein FUS | 20 | 16.2 | 1.46 | <0.001 | 1.67 | <0.001 | 1.25 | 0.001 |
| FYV1_HUMAN | 1-phosphatidylinositol 3-phosphate 5-kinase | 10 | 3.5 | 1.55 | <0.001 | 1.71 | <0.001 | 1.27 | <0.001 |
| FZR_HUMAN | Fizzy-related protein homolog | 3 | 11.9 | 1.32 | 0.047 | 1.45 | 0.075 | 1.19 | 0.102 |
| G3P_HUMAN | Glyceraldehyde-3-phosphate dehydrogenase | 10 | 10.7 | 1.44 | <0.001 | 1.54 | 0.006 | 1.01 | 0.940 |
| GAK_HUMAN | Cyclin-G-associated kinase | 4 | 3.2 | 1.34 | <0.001 | 1.53 | <0.001 | 1.03 | <0.001 |
| GATA6_HUMAN | Transcription factor GATA-6 | 14 | 9.6 | 1.14 | 0.199 | 1.34 | 0.001 | 1.11 | 0.230 |
| GCN1_HUMAN | eIF-2-alpha kinase activator GCN1 | 19 | 7 | 1.5 | <0.001 | 1.95 | <0.001 | 1.49 | <0.001 |
| GDIB_HUMAN | Rab GDP dissociation inhibitor beta | 4 | 2.5 | 1.21 | 0.005 | 1.15 | 0.012 | 0.86 | 0.119 |
| GDS1_HUMAN | Rap1 GTPase-GDP dissociation stimulator 1 | 2 | 4.9 | 1.27 | 0.012 | 1.45 | 0.005 | 1.08 | 0.424 |
| GFAP_HUMAN | Glial fibrillary acidic protein | 13 | 12 | 1.45 | <0.001 | 1.51 | 0.001 | 1.08 | 0.061 |
| GLCNE_HUMAN | Bifunctional UDP-N-acetylglucosamine 2-epimerase/N-acetylmannosamine kinase | 5 | 4.8 | 1.33 | 0.013 | 1.22 | 0.229 | 0.86 | 0.329 |
| GPV_HUMAN | Platelet glycoprotein V | 3 | 6.3 | 1.4 | 0.025 | 2.17 | 0.011 | 1.69 | 0.039 |
| GRHL3_HUMAN | Grainyhead-like protein 3 homolog | 3 | 4.3 | 1.47 | <0.001 | 1.89 | 0.001 | 1.52 | 0.014 |
| GRP78_HUMAN | 78 kDa glucose-regulated protein | 7 | 8.6 | 1.35 | <0.001 | 1.51 | <0.001 | 1.08 | 0.018 |
| GSK3A_HUMAN | Glycogen synthase kinase-3 alpha | 11 | 19 | 1.51 | <0.001 | 1.74 | <0.001 | 1.31 | 0.001 |
| GTF2I_HUMAN | General transcription factor II-I | 6 | 9.2 | 1.64 | 0.029 | 2.41 | 0.037 | 2.01 | 0.131 |
| GUC2D_HUMAN | Retinal guanylyl cyclase 1 | 8 | 6.1 | 1.36 | 0.040 | 1.76 | 0.027 | 1.25 | 0.358 |
| GUC2F_HUMAN | Retinal guanylyl cyclase 2 | 3 | 3.7 | 1.38 | <0.001 | 1.58 | 0.001 | 1.1 | 0.115 |
| H12_HUMAN | Histone H1.2 | 4 | 11.7 | 1.37 | 0.016 | 1.49 | 0.002 | 1.13 | 0.247 |
| H13_HUMAN | Histone H1.3 | 5 | 19.9 | 1.3 | <0.001 | 0.9 | 0.280 | 0.71 | 0.029 |
| HASP_HUMAN | Serine/threonine-protein kinase haspin | 2 | 2.1 | 1.39 | <0.001 | 1.42 | 0.002 | 1.04 | 0.050 |
| HDAC9_HUMAN | Histone deacetylase 9 | 2 | 1.1 | 1.46 | 0.002 | 1.56 | 0.005 | 1.12 | 0.129 |
| HECD1_HUMAN | E3 ubiquitin-protein ligase HECTD1 | 11 | 5.5 | 1.37 | <0.001 | 1.66 | <0.001 | 1.26 | 0.023 |
| HECD4_HUMAN | Probable E3 ubiquitin-protein ligase HECTD4 | 13 | 2.3 | 1.43 | 0.002 | 1.61 | 0.037 | 1.19 | 0.421 |
| HERC1_HUMAN | Probable E3 ubiquitin-protein ligase HERC1 | 11 | 2.4 | 1.43 | 0.010 | 1.61 | 0.006 | 1.19 | 0.254 |
| HERC2_HUMAN | E3 ubiquitin-protein ligase HERC2 | 7 | 2.1 | 1.4 | <0.001 | 1.41 | <0.001 | 0.89 | 0.090 |
| HERC3_HUMAN | Probable E3 ubiquitin-protein ligase HERC3 | 2 | 1 | 1.48 | 0.046 | 1.83 | 0.107 | 1.53 | 0.394 |
| HEY1_HUMAN | Hairy/enhancer-of-split related with YRPW motif protein 1 | 2 | 13.8 | 1.29 | <0.001 | 1.37 | <0.001 | 0.9 | <0.001 |
| HEYL_HUMAN | Hairy/enhancer-of-split related with YRPW motif-like protein | 1 | 6.4 | 1.32 | 0.054 | 1.55 | 0.215 | 1.16 | 0.772 |
| HIC1_HUMAN | Hypermethylated in cancer 1 protein | 4 | 14.7 | 1.38 | 0.006 | 1.51 | 0.001 | 1.19 | 0.073 |
| HIRA_HUMAN | Protein HIRA | 9 | 11 | 1.54 | <0.001 | 1.56 | 0.001 | 1.03 | 0.672 |
| HMCN2_HUMAN | Hemicentin-2 | 17 | 4.5 | 1.43 | <0.001 | 1.44 | <0.001 | 1.1 | 0.002 |
| HMDH_HUMAN | 3-hydroxy-3-methylglutaryl-coenzyme A reductase | 6 | 2.3 | 1.43 | <0.001 | 1.89 | 0.001 | 1.43 | 0.019 |
| HNRH2_HUMAN | Heterogeneous nuclear ribonucleoprotein H2 | 4 | 15.1 | 1.37 | 0.005 | 1.49 | 0.004 | 1.05 | 0.723 |
| HNRL1_HUMAN | Heterogeneous nuclear ribonucleoprotein U-like protein 1 | 3 | 5.6 | 1.37 | 0.017 | 1.47 | 0.033 | 0.97 | 0.840 |
| HNRPL_HUMAN | Heterogeneous nuclear ribonucleoprotein L | 20 | 18 | 1.51 | <0.001 | 1.71 | <0.001 | 1.24 | 0.001 |
| HNRPM_HUMAN | Heterogeneous nuclear ribonucleoprotein M | 41 | 31.9 | 1.4 | <0.001 | 1.62 | <0.001 | 1.19 | 0.007 |
| HNRPU_HUMAN | Heterogeneous nuclear ribonucleoprotein U | 11 | 8.7 | 1.44 | 0.001 | 1.83 | <0.001 | 1.37 | 0.001 |
| HOME2_HUMAN | Homer protein homolog 2 | 2 | 9.3 | 1.56 | <0.001 | 1.76 | 0.001 | 1.33 | 0.003 |
|  | Heat shock cognate 71 kDa protein | 12 | 17.3 | 1.51 | <0.001 | 1.83 | <0.001 | 1.41 | 0.001 |
| HUWE1_HUMAN | E3 ubiquitin-protein ligase HUWE1 | 11 | 3.3 | 1.42 | <0.001 | 1.61 | 0.006 | 1.2 | 0.145 |
| ID4_HUMAN | DNA-binding protein inhibitor ID-4 | 2 | 14.3 | 1.42 | 0.001 | 1.48 | <0.001 | 1.06 | 0.001 |
| IF4G1_HUMAN | Eukaryotic translation initiation factor 4 gamma 1 | 5 | 2.4 | 1.49 | 0.003 | 1.5 | 0.006 | 1 | 0.988 |
| IF4H_HUMAN | Eukaryotic translation initiation factor 4H | 4 | 7.3 | 1.23 | 0.006 | 1.41 | 0.050 | 1 | 0.986 |
| IL3RB_HUMAN | Cytokine receptor common subunit beta | 1 | 1.8 | 1.34 | <0.001 | 1.4 | 0.004 | 1.07 | <0.001 |
| ILF3_HUMAN | Interleukin enhancer-binding factor 3 | 5 | 5.3 | 1.36 | 0.090 | 1.37 | 0.049 | 0.89 | 0.401 |
| INO80_HUMAN | DNA helicase INO80 | 3 | 2.4 | 1.37 | <0.001 | 1.43 | 0.051 | 1.05 | 0.613 |
| IQEC2_HUMAN | IQ motif and SEC7 domain-containing protein 2 | 7 | 4.1 | 1.47 | <0.001 | 1.87 | <0.001 | 1.42 | 0.001 |
| IQEC3_HUMAN | IQ motif and SEC7 domain-containing protein 3 | 4 | 3.7 | 1.52 | 0.001 | 1.53 | <0.001 | 1.11 | 0.001 |
| IRS2_HUMAN | Insulin receptor substrate 2 | 9 | 11.8 | 1.41 | 0.002 | 1.55 | 0.019 | 1.1 | 0.604 |
| ITA11_HUMAN | Integrin alpha-11 | 4 | 3.6 | 1.49 | <0.001 | 1.53 | 0.046 | 1.07 | 0.742 |
| ITAV_HUMAN | Integrin alpha-V | 3 | 3.8 | 1.42 | 0.011 | 1.56 | 0.001 | 1.26 | 0.032 |
| ITPR3_HUMAN | Inositol 1;4;5-trisphosphate receptor type 3 | 9 | 2.4 | 1.34 | <0.001 | 1.48 | 0.002 | 1.03 | 0.658 |
| ITSN1_HUMAN | Intersectin-1 | 5 | 4.4 | 1.49 | <0.001 | 1.52 | 0.001 | 1.1 | 0.015 |
| ITSN2_HUMAN | Intersectin-2 | 6 | 5.2 | 1.33 | <0.001 | 1.69 | <0.001 | 1.25 | 0.014 |
| IWS1_HUMAN | Protein IWS1 homolog | 3 | 4.8 | 1.64 | 0.041 | 1.56 | 0.022 | 1.14 | 0.326 |
| JARD2_HUMAN | Protein Jumonji | 3 | 2.6 | 1.25 | <0.001 | 1.52 | 0.003 | 1.07 | 0.518 |
| JIP2_HUMAN | C-Jun-amino-terminal kinase-interacting protein 2 | 4 | 9.2 | 1.4 | <0.001 | 1.29 | <0.001 | 0.96 | 0.224 |
| K22E_HUMAN | Keratin; type II cytoskeletal 2 epidermal | 14 | 20 | 1.44 | <0.001 | 1.57 | <0.001 | 1.11 | 0.034 |
| K22O_HUMAN | Keratin; type II cytoskeletal 2 oral | 11 | 19.1 | 1.4 | 0.001 | 1.47 | 0.004 | 1.1 | 0.086 |
| K2C4_HUMAN | Keratin; type II cytoskeletal 4 | 3 | 12 | 1.4 | 0.002 | 1.59 | 0.005 | 1.16 | 0.291 |
| KANK1_HUMAN | KN motif and ankyrin repeat domain-containing protein 1 | 9 | 8.7 | 1.4 | 0.001 | 1.34 | 0.010 | 0.98 | 0.635 |
| KAPCA_HUMAN | cAMP-dependent protein kinase catalytic subunit alpha | 1 | 2.3 | 1.38 | 0.011 | 1.22 | <0.001 | 0.91 | 0.310 |
| KASH5_HUMAN | Protein KASH5 | 3 | 5.2 | 1.47 | <0.001 | 1.5 | <0.001 | 1.04 | 0.053 |
| KCC2B_HUMAN | Calcium/calmodulin-dependent protein kinase type II subunit beta | 5 | 6.8 | 1.46 | <0.001 | 1.82 | <0.001 | 1.41 | 0.001 |
| KCMA1_HUMAN | Calcium-activated potassium channel subunit alpha-1 | 22 | 5.2 | 1.45 | 0.023 | 1.55 | 0.067 | 1.12 | 0.541 |
| KDM2B_HUMAN | Lysine-specific demethylase 2B | 9 | 5.5 | 1.47 | <0.001 | 1.65 | 0.002 | 1.21 | 0.163 |
| KDM6A_HUMAN | Lysine-specific demethylase 6A | 6 | 6.9 | 1.39 | <0.001 | 1.64 | <0.001 | 1.2 | 0.025 |
| KIBRA_HUMAN | Protein KIBRA | 4 | 2.5 | 1.38 | <0.001 | 1.57 | 0.001 | 1.28 | 0.126 |
| KIF14_HUMAN | Kinesin-like protein KIF14 | 4 | 2.5 | 1.44 | <0.001 | 1.72 | <0.001 | 1.29 | 0.001 |
| KLH12_HUMAN | Kelch-like protein 12 | 5 | 7.9 | 1.4 | 0.004 | 1.73 | 0.003 | 1.28 | 0.141 |
| KLHL3_HUMAN | Kelch-like protein 3 | 4 | 8.3 | 1.36 | <0.001 | 1.49 | 0.022 | 1.08 | 0.737 |
| KMT2A_HUMAN | Histone-lysine N-methyltransferase 2A | 26 | 6 | 1.45 | <0.001 | 1.75 | 0.003 | 1.29 | 0.043 |
| KMT2B_HUMAN | Histone-lysine N-methyltransferase 2B | 18 | 6.6 | 1.47 | <0.001 | 1.63 | <0.001 | 1.18 | 0.002 |
| KMT2C_HUMAN | Histone-lysine N-methyltransferase 2C | 14 | 3.5 | 1.43 | 0.001 | 1.62 | 0.002 | 1.22 | 0.045 |
| KMT2D_HUMAN | Histone-lysine N-methyltransferase 2D | 11 | 2.1 | 1.47 | 0.001 | 1.87 | <0.001 | 1.36 | 0.005 |
| KPCD1_HUMAN | Serine/threonine-protein kinase D1 | 3 | 8.1 | 1.23 | 0.075 | 1.24 | 0.286 | 1 | 0.969 |
| KPTN_HUMAN | Kaptin | 2 | 8 | 1.7 | 0.008 | 2.22 | 0.032 | 1.96 | 0.115 |
| KSR2_HUMAN | Kinase suppressor of Ras 2 | 5 | 6 | 1.43 | <0.001 | 1.64 | <0.001 | 1.2 | 0.001 |
| LAMA1_HUMAN | Laminin subunit alpha-1 | 4 | 1.5 | 1.45 | 0.002 | 1.63 | 0.004 | 1.19 | 0.037 |
| LAMA3_HUMAN | Laminin subunit alpha-3 | 4 | 1.5 | 1.54 | 0.002 | 1.82 | 0.015 | 1.53 | 0.001 |
| LAMA5_HUMAN | Laminin subunit alpha-5 | 7 | 3.6 | 1.36 | 0.005 | 1.81 | 0.029 | 1.33 | 0.296 |
| LAMB3_HUMAN | Laminin subunit beta-3 | 9 | 10.4 | 1.24 | 0.267 | 1.56 | 0.015 | 1.2 | 0.020 |
| LC7L3_HUMAN | Luc7-like protein 3 | 2 | 3.7 | 1.55 | <0.001 | 1.76 | <0.001 | 1.25 | 0.001 |
| LEF1_HUMAN | Lymphoid enhancer-binding factor 1 | 4 | 16.3 | 1.52 | <0.001 | 1.89 | <0.001 | 1.51 | 0.002 |
| LIPA2_HUMAN | Liprin-alpha-2 | 9 | 8.7 | 1.44 | 0.016 | 1.75 | 0.012 | 1.35 | 0.144 |
| LIPB1_HUMAN | Liprin-beta-1 | 5 | 8.2 | 1.38 | 0.001 | 1.56 | 0.029 | 1.11 | 0.517 |
| LMBL1_HUMAN | Lethal(3)malignant brain tumor-like protein 1 | 4 | 9.6 | 1.27 | 0.136 | 1.45 | 0.018 | 1 | 0.996 |
| LMLN_HUMAN | Leishmanolysin-like peptidase | 4 | 3.1 | 1.51 | <0.001 | 1.43 | 0.022 | 1.33 | <0.001 |
| LMTK3_HUMAN | Serine/threonine-protein kinase LMTK3 | 4 | 5.1 | 1.48 | <0.001 | 1.72 | 0.001 | 1.17 | 0.100 |
| LRP1_HUMAN | Prolow-density lipoprotein receptor-related protein | 11 | 3.7 | 1.39 | <0.001 | 1.56 | <0.001 | 1.09 | 0.003 |
| LRP4_HUMAN | Low-density lipoprotein receptor-related protein 4 | 6 | 2.9 | 1.42 | <0.001 | 1.58 | <0.001 | 1.1 | 0.001 |
| LRP5_HUMAN | Low-density lipoprotein receptor-related protein 5 | 19 | 8.2 | 1.38 | 0.049 | 1.5 | 0.021 | 1.03 | 0.827 |
| LRRK2_HUMAN | Leucine-rich repeat serine/threonine-protein kinase 2 | 2 | 1 | 1.43 | <0.001 | 1.58 | 0.002 | 1.16 | 0.067 |
| LTK_HUMAN | Leukocyte tyrosine kinase receptor | 9 | 4.6 | 1.5 | <0.001 | 1.51 | 0.042 | 1.1 | 0.650 |
| M3K15_HUMAN | Mitogen-activated protein kinase kinasekinase 15 | 4 | 5.1 | 1.45 | 0.001 | 1.59 | 0.002 | 1.2 | 0.169 |
| MA7D3_HUMAN | MAP7 domain-containing protein 3 | 5 | 1.9 | 1.44 | 0.009 | 1.62 | 0.014 | 1.18 | 0.178 |
| MACF1_HUMAN | Microtubule-actin cross-linking factor 1; isoforms 1/2/3/5 | 15 | 2.9 | 1.43 | <0.001 | 1.67 | <0.001 | 1.25 | 0.001 |
| MAGI2_HUMAN | Membrane-associated guanylate kinase; WW and PDZ domain-containing protein 2 | 9 | 7.3 | 1.18 | 0.357 | 1.63 | 0.022 | 1.83 | 0.059 |
| MAGI3_HUMAN | Membrane-associated guanylate kinase; WW and PDZ domain-containing protein 3 | 3 | 3.3 | 1.48 | <0.001 | 1.69 | <0.001 | 1.22 | <0.001 |
| MAP1B_HUMAN | Microtubule-associated protein 1B | 7 | 4.3 | 1.43 | <0.001 | 1.55 | 0.003 | 1.14 | 0.296 |
| MAP6_HUMAN | Microtubule-associated protein 6 | 7 | 7.9 | 1.47 | <0.001 | 1.68 | 0.001 | 1.25 | 0.160 |
| MARK3_HUMAN | MAP/microtubule affinity-regulating kinase 3 | 7 | 8.9 | 1.35 | <0.001 | 1.53 | 0.023 | 1.02 | 0.893 |
| MAST2_HUMAN | Microtubule-associated serine/threonine-protein kinase 2 | 4 | 3.9 | 1.49 | 0.001 | 1.55 | 0.005 | 1.07 | 0.262 |
| MAST3_HUMAN | Microtubule-associated serine/threonine-protein kinase 3 | 18 | 8.5 | 1.41 | <0.001 | 1.72 | 0.001 | 1.32 | 0.006 |
| MAST4_HUMAN | Microtubule-associated serine/threonine-protein kinase 4 | 8 | 1.6 | 1.41 | <0.001 | 1.57 | <0.001 | 1.17 | 0.001 |
| MBD4_HUMAN | Methyl-CpG-binding domain protein 4 | 5 | 2.4 | 1.42 | <0.001 | 1.92 | 0.021 | 1.42 | 0.172 |
| MBNL3_HUMAN | Muscleblind-like protein 3 | 3 | 11.6 | 1.44 | <0.001 | 1.42 | <0.001 | 1.01 | 0.847 |
| MCM7_HUMAN | DNA replication licensing factor MCM7 | 5 | 11.3 | 1.45 | <0.001 | 1.61 | <0.001 | 1.16 | 0.051 |
| MED1_HUMAN | Mediator of RNA polymerase II transcription subunit 1 | 11 | 10.2 | 1.2 | <0.001 | 1.66 | 0.041 | 1.21 | 0.508 |
| MED14_HUMAN | Mediator of RNA polymerase II transcription subunit 14 | 4 | 2.7 | 1.54 | <0.001 | 1.94 | 0.003 | 1.43 | 0.028 |
| MIA3_HUMAN | Melanoma inhibitory activity protein 3 | 8 | 5.7 | 1.46 | <0.001 | 1.81 | <0.001 | 1.36 | 0.001 |
| MICA_HUMAN | MHC class I polypeptide-related sequence A | 1 | 2.6 | 1.37 | <0.001 | 1.6 | 0.004 | 1.23 | 0.171 |
| MICA2_HUMAN | Protein-methionine sulfoxide oxidase MICAL2 | 5 | 4.7 | 1.34 | 0.008 | 1.59 | 0.006 | 1.18 | 0.230 |
| MINK1_HUMAN | Misshapen-like kinase 1 | 11 | 9.8 | 1.36 | <0.001 | 1.6 | <0.001 | 1.15 | 0.042 |
| MINT_HUMAN | Msx2-interacting protein | 10 | 3.1 | 1.42 | <0.001 | 1.58 | <0.001 | 1.16 | <0.001 |
| MK01_HUMAN | Mitogen-activated protein kinase 1 | 8 | 6.7 | 1.3 | 0.013 | 1.61 | 0.037 | 1.15 | 0.531 |
| MK04_HUMAN | Mitogen-activated protein kinase 4 | 4 | 9.9 | 1.51 | <0.001 | 1.62 | <0.001 | 1.23 | 0.002 |
| MMRN1_HUMAN | Multimerin-1 | 8 | 10.5 | 1.38 | <0.001 | 1.81 | 0.056 | 1.36 | 0.372 |
| MOES_HUMAN | Moesin | 4 | 6.6 | 1.28 | 0.074 | 1.35 | 0.068 | 1.02 | 0.762 |
| MPDZ_HUMAN | Multiple PDZ domain protein | 6 | 2.2 | 1.37 | 0.001 | 2.07 | 0.067 | 1.54 | 0.308 |
| MPIP2_HUMAN | M-phase inducer phosphatase 2 | 3 | 2.6 | 1.43 | 0.046 | 1.33 | 0.305 | 0.94 | 0.828 |
| MPRIP_HUMAN | Myosin phosphatase Rho-interacting protein | 7 | 7.3 | 1.57 | 0.002 | 1.6 | 0.001 | 1.27 | <0.001 |
| MRCKA_HUMAN | Serine/threonine-protein kinase MRCK alpha | 5 | 2.5 | 1.58 | <0.001 | 1.71 | 0.004 | 1.3 | 0.111 |
| MRCKG_HUMAN | Serine/threonine-protein kinase MRCK gamma | 8 | 5.6 | 1.47 | <0.001 | 1.77 | <0.001 | 1.31 | 0.002 |
| MSH3_HUMAN | DNA mismatch repair protein Msh3 | 7 | 6.8 | 1.42 | 0.005 | 1.6 | <0.001 | 1.22 | 0.005 |
| MTAP2_HUMAN | Microtubule-associated protein 2 | 14 | 8.8 | 1.47 | <0.001 | 1.5 | 0.036 | 1.05 | 0.772 |
| MTMR1_HUMAN | Myotubularin-related protein 1 | 5 | 9.5 | 1.24 | 0.114 | 1.63 | 0.007 | 1.25 | 0.089 |
| MTMR5_HUMAN | Myotubularin-related protein 5 | 5 | 3.6 | 1.78 | <0.001 | 2.53 | 0.004 | 2.09 | 0.027 |
| MTOR_HUMAN | Serine/threonine-protein kinase mTOR | 14 | 6.3 | 1.36 | 0.038 | 1.51 | 0.049 | 1.09 | 0.663 |
| MTP_HUMAN | Microsomal triglyceride transfer protein large subunit | 4 | 6.3 | 1.4 | <0.001 | 1.68 | <0.001 | 1.29 | 0.004 |
| MUC16_HUMAN | Mucin-16 | 51 | 3.2 | 1.5 | <0.001 | 1.75 | 0.002 | 1.33 | 0.036 |
| MUC4_HUMAN | Mucin-4 | 5 | 3.4 | 1.39 | <0.001 | 1.43 | 0.001 | 1.02 | 0.650 |
| MUC5A_HUMAN | Mucin-5AC | 15 | 3.7 | 1.42 | 0.004 | 1.7 | <0.001 | 1.24 | 0.014 |
| MYH14_HUMAN | Myosin-14 | 15 | 6.5 | 1.48 | <0.001 | 1.55 | 0.010 | 1.1 | 0.445 |
| MYH3_HUMAN | Myosin-3 | 4 | 3.2 | 1.48 | 0.002 | 1.49 | 0.186 | 1.06 | 0.841 |
| MYH9_HUMAN | Myosin-9 | 18 | 4.2 | 1.51 | <0.001 | 1.73 | 0.002 | 1.26 | 0.064 |
| MYLK_HUMAN | Myosin light chain kinase; smooth muscle | 5 | 2.4 | 1.02 | 0.937 | 1.62 | <0.001 | 1.3 | 0.021 |
| MYLK3_HUMAN | Myosin light chain kinase 3 | 5 | 5.9 | 1.42 | <0.001 | 1.73 | 0.002 | 1.31 | 0.058 |
| MYO3B_HUMAN | Myosin-IIIb | 5 | 2.5 | 1.33 | <0.001 | 1.62 | 0.002 | 1.21 | 0.038 |
| MYO9B_HUMAN | Unconventional myosin-IXb | 6 | 3.8 | 1.46 | 0.003 | 1.8 | <0.001 | 1.41 | <0.001 |
| NACAM_HUMAN | Nascent polypeptide-associated complex subunit alpha; muscle-specific form | 7 | 4.3 | 1.42 | 0.002 | 1.55 | <0.001 | 1.25 | 0.050 |
| NCOA2_HUMAN | Nuclear receptor coactivator 2 | 6 | 5.1 | 1.4 | 0.001 | 1.53 | <0.001 | 1.08 | 0.105 |
| NCOA6_HUMAN | Nuclear receptor coactivator 6 | 13 | 6.1 | 1.54 | 0.001 | 1.75 | 0.021 | 1.26 | 0.250 |
| NCOR2_HUMAN | Nuclear receptor corepressor 2 | 6 | 4.6 | 1.46 | <0.001 | 1.63 | 0.001 | 1.18 | 0.007 |
| NDKB_HUMAN | Nucleoside diphosphate kinase B | 3 | 19.1 | 1.56 | <0.001 | 1.8 | <0.001 | 1.41 | 0.004 |
| NF1_HUMAN | Neurofibromin | 6 | 1.3 | 1.42 | 0.003 | 1.52 | 0.004 | 1.1 | 0.181 |
| NF2IP_HUMAN | NFATC2-interacting protein | 1 | 4.8 | 0.54 | 0.233 | 0.57 | 0.285 | 0.23 | 0.015 |
| NFH_HUMAN | Neurofilament heavy polypeptide | 4 | 5.3 | 1.16 | 0.375 | 1.76 | 0.013 | 1.02 | 0.030 |
| NFX1_HUMAN | Transcriptional repressor NF-X1 | 2 | 2.5 | 1.44 | <0.001 | 1.45 | <0.001 | 1.11 | 0.322 |
| NGN3_HUMAN | Neurogenin-3 | 2 | 7 | 1.67 | 0.001 | 1.59 | 0.125 | 1.54 | 0.065 |
| NHRF1_HUMAN | Na(+)/H(+) exchange regulatory cofactor NHE-RF1 | 4 | 17.3 | 1.55 | 0.006 | 1.84 | 0.005 | 1.42 | 0.099 |
| NKAP_HUMAN | NF-kappa-B-activating protein | 5 | 7.2 | 1.48 | <0.001 | 1.64 | <0.001 | 1.24 | <0.001 |
| NMDE1_HUMAN | Glutamate receptor ionotropic; NMDA 2A | 8 | 4.7 | 1.41 | 0.025 | 1.45 | 0.006 | 0.98 | 0.818 |
| NONO_HUMAN | Non-POU domain-containing octamer-binding protein | 8 | 10.6 | 1.48 | <0.001 | 1.64 | 0.001 | 1.35 | <0.001 |
| NOTC1_HUMAN | Neurogenic locus notch homolog protein 1 | 5 | 2.2 | 1.4 | <0.001 | 1.66 | <0.001 | 1.28 | 0.032 |
| NOX5_HUMAN | NADPH oxidase 5 | 3 | 5.2 | 1.4 | 0.054 | 1.47 | <0.001 | 1.07 | 0.001 |
| NPHP4_HUMAN | Nephrocystin-4 | 3 | 3 | 1.33 | 0.276 | 1.64 | 0.002 | 1.36 | <0.001 |
| NR2E3_HUMAN | Photoreceptor-specific nuclear receptor | 6 | 11.5 | 1.4 | 0.012 | 1.39 | 0.215 | 1.03 | 0.907 |
| NR2F6_HUMAN | Nuclear receptor subfamily 2 group F member 6 | 3 | 10.6 | 1.24 | 0.317 | 1.72 | 0.093 | 1.37 | 0.224 |
| NRG2_HUMAN | Pro-neuregulin-2; membrane-bound isoform | 6 | 8.2 | 1.51 | 0.001 | 1.56 | <0.001 | 1.7 | 0.154 |
| NRP2_HUMAN | Neuropilin-2 | 3 | 2.9 | 1.41 | <0.001 | 1.42 | <0.001 | 1 | 0.144 |
| NRX1B_HUMAN | Neurexin-1-beta | 6 | 5.9 | 1.2 | 0.340 | 1.48 | 0.068 | 1.17 | 0.308 |
| NUAK1_HUMAN | NUAK family SNF1-like kinase 1 | 4 | 9.4 | 1.34 | <0.001 | 1.68 | 0.011 | 1.17 | 0.228 |
| NUCL_HUMAN | Nucleolin | 5 | 4.9 | 1.51 | <0.001 | 1.65 | 0.004 | 1.22 | 0.132 |
| OBSCN_HUMAN | Obscurin | 30 | 5.1 | 1.4 | <0.001 | 1.44 | <0.001 | 1.04 | 0.484 |
| OBSL1_HUMAN | Obscurin-like protein 1 | 6 | 4.3 | 1.49 | <0.001 | 1.83 | 0.002 | 1.37 | 0.072 |
| OCRL_HUMAN | Inositol polyphosphate 5-phosphatase OCRL-1 | 4 | 7.3 | 1.52 | <0.001 | 1.7 | 0.001 | 1.41 | 0.001 |
| OGT1_HUMAN | UDP-N-acetylglucosamine--peptide N-acetylglucosaminyltransferase 110 kDa subunit | 2 | 4.1 | 1.31 | 0.174 | 1.61 | 0.087 | 1.22 | 0.566 |
| OTU7B_HUMAN | OTU domain-containing protein 7B | 6 | 10.4 | 1.38 | 0.002 | 1.57 | 0.001 | 1.09 | 0.249 |
| PABP2_HUMAN | Polyadenylate-binding protein 2 | 8 | 7.5 | 1.51 | 0.001 | 1.73 | 0.011 | 1.22 | 0.225 |
| PAI1_HUMAN | Plasminogen activator inhibitor 1 | 5 | 16.7 | 1.23 | 0.001 | 1.28 | 0.183 | 1.03 | 0.890 |
| PAK3_HUMAN | Serine/threonine-protein kinase PAK 3 | 4 | 7.7 | 1.44 | <0.001 | 1.51 | 0.002 | 1.11 | 0.020 |
| PAK4_HUMAN | Serine/threonine-protein kinase PAK 4 | 4 | 4.9 | 1.38 | 0.001 | 1.77 | 0.010 | 1.25 | 0.213 |
| PARI_HUMAN | PCNA-interacting partner | 2 | 6.2 | 1.54 | 0.001 | 1.96 | <0.001 | 1.61 | <0.001 |
| PBX3_HUMAN | Pre-B-cell leukemia transcription factor 3 | 2 | 16.6 | 0.97 | 0.868 | 1.43 | <0.001 | 1.22 | 0.004 |
| PCD16_HUMAN | Protocadherin-16 | 5 | 2.9 | 1.46 | <0.001 | 1.6 | 0.001 | 1.19 | 0.052 |
| PCDA8_HUMAN | Protocadherin alpha-8 | 4 | 5.9 | 1.34 | <0.001 | 1.41 | <0.001 | 1.07 | 0.171 |
| PCGF6_HUMAN | Polycomb group RING finger protein 6 | 3 | 4.6 | 1.41 | 0.001 | 1.53 | 0.002 | 1.09 | 0.016 |
| PCLO_HUMAN | Protein piccolo | 18 | 5.9 | 1.43 | <0.001 | 1.63 | <0.001 | 1.18 | <0.001 |
| PDE3A_HUMAN | cGMP-inhibited 3~;5~-cyclic phosphodiesterase A | 2 | 3.2 | 1.36 | <0.001 | 1.32 | 0.015 | 0.96 | 0.517 |
| PDE3B_HUMAN | cGMP-inhibited 3~;5~-cyclic phosphodiesterase B | 1 | 1.4 | 1.66 | 0.018 | 1.74 | 0.185 | 1.38 | 0.525 |
| PDZD2_HUMAN | PDZ domain-containing protein 2 | 21 | 10.4 | 1.35 | <0.001 | 1.55 | 0.010 | 1.1 | 0.502 |
| PEAK1_HUMAN | Pseudopodium-enriched atypical kinase 1 | 7 | 5 | 1.47 | <0.001 | 1.74 | <0.001 | 1.3 | 0.001 |
| PEG3_HUMAN | Paternally-expressed gene 3 protein | 6 | 2.6 | 1.49 | <0.001 | 1.62 | <0.001 | 1.2 | 0.013 |
| PEPL_HUMAN | Periplakin | 5 | 2.3 | 1.46 | <0.001 | 1.69 | 0.001 | 1.21 | 0.063 |
| PER1_HUMAN | Period circadian protein homolog 1 | 4 | 1.6 | 1.51 | <0.001 | 1.47 | 0.201 | 1.43 | 0.005 |
| PGBM_HUMAN | Basement membrane-specific heparansulfate proteoglycan core protein | 11 | 3.4 | 1.42 | <0.001 | 1.64 | 0.002 | 1.25 | 0.049 |
| PGFRB_HUMAN | Platelet-derived growth factor receptor beta | 6 | 4.8 | 1.34 | 0.024 | 1.35 | 0.071 | 1.69 | 0.167 |
| PGP_HUMAN | Glycerol-3-phosphate phosphatase | 4 | 16.5 | 1.51 | <0.001 | 1.67 | <0.001 | 1.19 | 0.016 |
| PHAR4_HUMAN | Phosphatase and actin regulator 4 | 9 | 9.7 | 1.51 | <0.001 | 1.57 | 0.002 | 1.06 | 0.598 |
| PHF14_HUMAN | PHD finger protein 14 | 5 | 6.1 | 1.42 | <0.001 | 1.65 | 0.003 | 1.22 | 0.081 |
| PHLB2_HUMAN | Pleckstrin homology-like domain family B member 2 | 5 | 5.6 | 1.59 | 0.007 | 2.09 | 0.019 | 1.66 | 0.067 |
| PHLP1_HUMAN | PH domain leucine-rich repeat-containing protein phosphatase 1 | 6 | 3.2 | 1.41 | 0.001 | 1.61 | 0.005 | 1.23 | 0.087 |
| PI42C_HUMAN | Phosphatidylinositol 5-phosphate 4-kinase type-2 gamma | 3 | 11.2 | 1.54 | 0.005 | 1.35 | <0.001 | 1.13 | 0.084 |
| PI4KA_HUMAN | Phosphatidylinositol 4-kinase alpha | 8 | 3.8 | 1.42 | <0.001 | 1.66 | <0.001 | 1.22 | <0.001 |
| PI5PA_HUMAN | Phosphatidylinositol 4;5-bisphosphate 5-phosphatase A | 6 | 4.4 | 1.57 | <0.001 | 1.74 | <0.001 | 1.23 | <0.001 |
| PK3CD_HUMAN | Phosphatidylinositol 4;5-bisphosphate 3-kinase catalytic subunit delta isoform | 8 | 4.6 | 1.51 | <0.001 | 1.69 | 0.004 | 1.24 | 0.095 |
| PKD1_HUMAN | Polycystin-1 | 19 | 4.3 | 1.37 | 0.005 | 1.64 | <0.001 | 1.21 | <0.001 |
| PKHG3_HUMAN | Pleckstrin homology domain-containing family G member 3 | 6 | 4.1 | 1.49 | <0.001 | 1.7 | <0.001 | 1.28 | 0.003 |
| PKP1_HUMAN | Plakophilin-1 | 5 | 7.8 | 1.33 | 0.020 | 1.53 | 0.002 | 1.04 | 0.466 |
| PLCB3_HUMAN | 1-phosphatidylinositol 4;5-bisphosphate phosphodiesterase beta-3 | 6 | 4.5 | 1.49 | <0.001 | 1.66 | <0.001 | 1.2 | 0.001 |
| PLCB4_HUMAN | 1-phosphatidylinositol 4;5-bisphosphate phosphodiesterase beta-4 | 4 | 4.2 | 1.36 | 0.031 | 1.78 | 0.003 | 1.38 | 0.052 |
| PLCL2_HUMAN | Inactive phospholipase C-like protein 2 | 5 | 2.6 | 1.37 | 0.001 | 1.71 | 0.017 | 1.19 | 0.470 |
| PLCZ1_HUMAN | 1-phosphatidylinositol 4;5-bisphosphate phosphodiesterase zeta-1 | 2 | 5.3 | 1.31 | 0.066 | 1.36 | 0.087 | 0.86 | 0.497 |
| PLEC_HUMAN | Plectin | 16 | 3.6 | 1.42 | 0.003 | 1.6 | 0.005 | 1.17 | 0.066 |
| PLSL_HUMAN | Plastin-2 | 12 | 13.7 | 1.32 | <0.001 | 1.08 | 0.059 | 0.74 | 0.011 |
| PLXA4_HUMAN | Plexin-A4 | 7 | 6.4 | 1.43 | <0.001 | 1.58 | <0.001 | 1.13 | 0.145 |
| PLXB3_HUMAN | Plexin-B3 | 2 | 1.9 | 1.48 | 0.022 | 1.7 | 0.154 | 1.35 | 0.434 |
| PLXD1_HUMAN | Plexin-D1 | 4 | 2.6 | 1.47 | <0.001 | 1.62 | <0.001 | 1.1 | 0.065 |
| PNPT1_HUMAN | Polyribonucleotide nucleotidyltransferase 1; mitochondrial | 6 | 5.4 | 1.46 | <0.001 | 1.59 | 0.023 | 1.16 | 0.342 |
| PO4F2_HUMAN | POU domain; class 4; transcription factor 2 | 13 | 19.8 | 1.4 | <0.001 | 1.85 | <0.001 | 1.41 | 0.006 |
| PP1A_HUMAN | Serine/threonine-protein phosphatase PP1-alpha catalytic subunit | 8 | 11.2 | 1.6 | 0.002 | 1.79 | 0.003 | 1.32 | 0.035 |
| PPM1B_HUMAN | Protein phosphatase 1B | 7 | 2.9 | 1.63 | <0.001 | 1.75 | <0.001 | 1.28 | <0.001 |
| PPM1H_HUMAN | Protein phosphatase 1H | 4 | 15.2 | 1.3 | 0.018 | 1.61 | <0.001 | 1.1 | 0.346 |
| PPM1L_HUMAN | Protein phosphatase 1L | 3 | 11.4 | 1.45 | <0.001 | 2.02 | <0.001 | 1.58 | 0.004 |
| PRGR_HUMAN | Progesterone receptor | 7 | 10 | 1.54 | 0.001 | 1.97 | 0.001 | 1.58 | 0.051 |
| PRKDC_HUMAN | DNA-dependent protein kinase catalytic subunit | 16 | 4.4 | 1.28 | 0.060 | 1.56 | 0.001 | 1.24 | 0.156 |
| PRP8_HUMAN | Pre-mRNA-processing-splicing factor 8 | 6 | 3.3 | 1.38 | <0.001 | 1.48 | <0.001 | 1.03 | 0.004 |
| PRR5_HUMAN | Proline-rich protein 5 | 3 | 7 | 1.34 | <0.001 | 1.6 | 0.002 | 1.36 | 0.072 |
| PRS4_HUMAN | 26S protease regulatory subunit 4 | 6 | 3.4 | 1.52 | 0.015 | 1.62 | 0.009 | 1.32 | 0.083 |
| PSB10_HUMAN | Proteasome subunit beta type-10 | 4 | 6.6 | 1.26 | <0.001 | 1.5 | <0.001 | 0.93 | 0.021 |
| PSB3_HUMAN | Proteasome subunit beta type-3 | 13 | 13.2 | 1.44 | <0.001 | 1.66 | <0.001 | 1.14 | 0.003 |
| PSPC1_HUMAN | Paraspeckle component 1 | 4 | 15.1 | 1.39 | <0.001 | 1.48 | 0.003 | 1.02 | 0.585 |
| PTBP2_HUMAN | Polypyrimidine tract-binding protein 2 | 3 | 3.8 | 1.39 | <0.001 | 1.43 | 0.001 | 0.98 | 0.183 |
| PTN1_HUMAN | Tyrosine-protein phosphatase non-receptor type 1 | 1 | 2.5 | 1.05 | 0.795 | 1.06 | 0.489 | 0.78 | 0.040 |
| PTN12_HUMAN | Tyrosine-protein phosphatase non-receptor type 12 | 5 | 9 | 1.45 | <0.001 | 1.76 | <0.001 | 1.32 | 0.018 |
| PTN18_HUMAN | Tyrosine-protein phosphatase non-receptor type 18 | 3 | 8.5 | 1.43 | <0.001 | 1.61 | <0.001 | 1.14 | 0.004 |
| PTN6_HUMAN | Tyrosine-protein phosphatase non-receptor type 6 | 4 | 5.2 | 1.35 | 0.053 | 1.73 | 0.006 | 1.42 | 0.005 |
| PTPC1_HUMAN | Protein tyrosine phosphatase domain-containing protein 1 | 2 | 2.9 | 1.43 | <0.001 | 1.78 | 0.004 | 1.36 | 0.084 |
| PTPRB_HUMAN | Receptor-type tyrosine-protein phosphatase beta | 17 | 9.5 | 1.49 | <0.001 | 1.62 | <0.001 | 1.16 | 0.001 |
| PTPRE_HUMAN | Receptor-type tyrosine-protein phosphatase epsilon | 4 | 9.6 | 1.4 | 0.004 | 1.4 | 0.002 | 0.96 | 0.527 |
| PTPRG_HUMAN | Receptor-type tyrosine-protein phosphatase gamma | 6 | 2.2 | 1.45 | 0.002 | 1.41 | 0.003 | 0.98 | 0.823 |
| PTPRQ_HUMAN | Phosphatidylinositol phosphatase PTPRQ | 5 | 3.4 | 1.39 | <0.001 | 1.62 | <0.001 | 1.11 | 0.071 |
| PTPRU_HUMAN | Receptor-type tyrosine-protein phosphatase U | 4 | 4.7 | 1.42 | <0.001 | 1.65 | <0.001 | 1.14 | <0.001 |
| PYR1_HUMAN | CAD protein | 4 | 3.3 | 1.2 | 0.085 | 1.17 | 0.106 | 0.84 | 0.031 |
| RAB1A_HUMAN | Ras-related protein Rab-1A | 6 | 12.2 | 1.45 | <0.001 | 1.74 | <0.001 | 1.32 | <0.001 |
| RABE2_HUMAN | RabGTPase-binding effector protein 2 | 3 | 3.9 | 1.5 | <0.001 | 1.79 | <0.001 | 1.34 | 0.010 |
| RAVR2_HUMAN | Ribonucleoprotein PTB-binding 2 | 4 | 5.8 | 1.36 | 0.018 | 1.65 | <0.001 | 1.37 | 0.037 |
| RB15B_HUMAN | Putative RNA-binding protein 15B | 9 | 9.7 | 1.45 | <0.001 | 1.56 | 0.002 | 1.15 | 0.158 |
| RBBP6_HUMAN | E3 ubiquitin-protein ligase RBBP6 | 5 | 3.9 | 1.45 | 0.004 | 1.75 | <0.001 | 1.34 | <0.001 |
| RBM15_HUMAN | Putative RNA-binding protein 15 | 17 | 10.3 | 1.42 | <0.001 | 1.68 | 0.001 | 1.24 | 0.024 |
| RBM23_HUMAN | Probable RNA-binding protein 23 | 4 | 8.7 | 1.46 | 0.002 | 1.46 | 0.075 | 1.11 | 0.650 |
| RBMX_HUMAN | RNA-binding motif protein; X chromosome | 6 | 9.7 | 1.4 | 0.002 | 1.46 | 0.018 | 1.1 | 0.213 |
| RCOR3_HUMAN | REST corepressor 3 | 2 | 6.5 | 1.49 | <0.001 | 1.56 | 0.018 | 1.09 | 0.588 |
| RELB_HUMAN | Transcription factor RelB | 6 | 5.4 | 1.52 | 0.002 | 1.73 | 0.011 | 1.3 | 0.173 |
| RENT1_HUMAN | Regulator of nonsense transcripts 1 | 6 | 3.8 | 1.61 | <0.001 | 1.88 | 0.001 | 1.46 | 0.003 |
| RGMC_HUMAN | Hemojuvelin | 3 | 6.8 | 1.55 | <0.001 | 1.72 | <0.001 | 1.25 | <0.001 |
| RGPS1_HUMAN | Ras-specific guanine nucleotide-releasing factor RalGPS1 | 4 | 3.2 | 1.39 | <0.001 | 1.59 | <0.001 | 1.15 | 0.004 |
| RGS20_HUMAN | Regulator of G-protein signaling 20 | 3 | 15.2 | 1.24 | <0.001 | 1.3 | <0.001 | 0.91 | 0.395 |
| RGS3_HUMAN | Regulator of G-protein signaling 3 | 5 | 4.2 | 1.33 | 0.010 | 1.57 | 0.001 | 1.12 | 0.018 |
| RHG09_HUMAN | Rho GTPase-activating protein 9 | 2 | 5.6 | 1.3 | 0.109 | 1.37 | <0.001 | 1.04 | 0.594 |
| RHG21_HUMAN | Rho GTPase-activating protein 21 | 9 | 4.8 | 1.42 | <0.001 | 1.7 | <0.001 | 1.3 | <0.001 |
| RHG24_HUMAN | Rho GTPase-activating protein 24 | 6 | 10.7 | 1.44 | <0.001 | 1.76 | <0.001 | 1.33 | <0.001 |
| RHG28_HUMAN | Rho GTPase-activating protein 28 | 4 | 3.7 | 1.31 | 0.087 | 1.51 | 0.031 | 1.11 | 0.271 |
| RHG31_HUMAN | Rho GTPase-activating protein 31 | 7 | 5.9 | 1.41 | <0.001 | 1.59 | <0.001 | 1.16 | 0.010 |
| RHG33_HUMAN | Rho GTPase-activating protein 33 | 6 | 5.4 | 1.44 | <0.001 | 1.68 | 0.001 | 1.26 | 0.003 |
| RIC8A_HUMAN | Synembryn-A | 2 | 2.1 | 1.66 | 0.153 | 2.02 | 0.067 | 1.67 | 0.166 |
| RIN1_HUMAN | Ras and Rab interactor 1 | 9 | 7 | 1.47 | 0.001 | 1.62 | 0.004 | 1.17 | 0.147 |
| RMXL1_HUMAN | RNA binding motif protein; X-linked-like-1 | 8 | 14.9 | 1.51 | <0.001 | 1.84 | <0.001 | 1.39 | <0.001 |
| RN169_HUMAN | E3 ubiquitin-protein ligase RNF169 | 4 | 10.3 | 1.5 | 0.002 | 1.64 | <0.001 | 1.18 | 0.010 |
| RN19A_HUMAN | E3 ubiquitin-protein ligase RNF19A | 11 | 8.1 | 1.38 | 0.003 | 1.58 | <0.001 | 1.2 | <0.001 |
| RNPS1_HUMAN | RNA-binding protein with serine-rich domain 1 | 3 | 14.1 | 1.42 | <0.001 | 1.44 | 0.010 | 1.25 | 0.025 |
| ROA0_HUMAN | Heterogeneous nuclear ribonucleoprotein A0 | 22 | 19.3 | 1 | 0.991 | 0.86 | <0.001 | 0.63 | 0.007 |
| ROA1_HUMAN | Heterogeneous nuclear ribonucleoprotein A1 | 7 | 14.8 | 1.47 | <0.001 | 1.83 | <0.001 | 1.36 | <0.001 |
| ROA3_HUMAN | Heterogeneous nuclear ribonucleoprotein A3 | 8 | 19.8 | 1.38 | <0.001 | 1.35 | 0.030 | 1 | 0.970 |
| ROBO4_HUMAN | Roundabout homolog 4 | 6 | 1.2 | 1.45 | <0.001 | 1.65 | 0.001 | 1.2 | 0.045 |
| ROCK2_HUMAN | Rho-associated protein kinase 2 | 4 | 2.8 | 1.35 | <0.001 | 1.5 | 0.001 | 1.11 | 0.028 |
| RP1L1_HUMAN | Retinitis pigmentosa 1-like 1 protein | 14 | 7.4 | 1.35 | 0.001 | 1.62 | <0.001 | 1.16 | <0.001 |
| RRP5_HUMAN | Protein RRP5 homolog | 3 | 1.5 | 1.1 | <0.001 | 0.94 | 0.008 | 0.55 | <0.001 |
| RS2_HUMAN | 40S ribosomal protein S2 | 13 | 11.9 | 1.45 | <0.001 | 1.69 | <0.001 | 1.12 | 0.005 |
| RSSA_HUMAN | 40S ribosomal protein SA | 3 | 15.3 | 1.5 | <0.001 | 1.69 | <0.001 | 1.26 | <0.001 |
| RUNX1_HUMAN | Runt-related transcription factor 1 | 8 | 8.4 | 1.59 | <0.001 | 1.73 | <0.001 | 1.3 | 0.001 |
| RYR2_HUMAN | Ryanodine receptor 2 | 16 | 3.6 | 1.45 | <0.001 | 1.72 | <0.001 | 1.27 | <0.001 |
| S27A1_HUMAN | Long-chain fatty acid transport protein 1 | 2 | 5.3 | 1.36 | 0.001 | 1.55 | 0.009 | 1.22 | 0.051 |
| SAC2_HUMAN | Phosphatidylinositide phosphatase SAC2 | 3 | 1 | 1.49 | <0.001 | 1.8 | <0.001 | 1.33 | 0.004 |
| SAFB1_HUMAN | Scaffold attachment factor B1 | 10 | 6.2 | 1.47 | <0.001 | 1.64 | <0.001 | 1.23 | 0.011 |
| SAHH2_HUMAN | Adenosylhomocysteinase 2 | 6 | 8.7 | 1.38 | <0.001 | 1.51 | <0.001 | 1.08 | 0.010 |
| SC61B_HUMAN | Protein transport protein Sec61 subunit beta | 2 | 16.7 | 1.34 | 0.004 | 1.59 | 0.029 | 1.22 | 0.144 |
| SCRT1_HUMAN | Transcriptional repressor scratch 1 | 8 | 27.9 | 1.31 | 0.104 | 1.62 | <0.001 | 1.26 | <0.001 |
| SCRT2_HUMAN | Transcriptional repressor scratch 2 | 5 | 7.5 | 1.43 | 0.001 | 1.62 | 0.001 | 1.24 | 0.048 |
| SET1B_HUMAN | Histone-lysine N-methyltransferase SETD1B | 5 | 2.7 | 1.4 | 0.003 | 1.44 | 0.057 | 1.06 | 0.627 |
| SETMR_HUMAN | Histone-lysine N-methyltransferase SETMAR | 2 | 5.1 | 1.56 | <0.001 | 2.13 | <0.001 | 1.76 | 0.026 |
| SF3B3_HUMAN | Splicing factor 3B subunit 3 | 2 | 3 | 1.5 | <0.001 | 1.78 | <0.001 | 1.27 | 0.001 |
| SF3B4_HUMAN | Splicing factor 3B subunit 4 | 1 | 4.7 | 1.46 | 0.005 | 1.86 | 0.002 | 1.39 | 0.038 |
| SF3B4_HUMAN | Splicing factor; proline- and glutamine-rich | 19 | 14.7 | 1.33 | <0.001 | 1.57 | <0.001 | 1.23 | <0.001 |
| SFR19_HUMAN | Splicing factor; arginine/serine-rich 19 | 14 | 4.6 | 1.42 | 0.005 | 1.65 | <0.001 | 1.17 | 0.002 |
| SH3K1_HUMAN | SH3 domain-containing kinase-binding protein 1 | 5 | 4.7 | 1.44 | 0.005 | 1.6 | <0.001 | 1.13 | 0.003 |
| SHH_HUMAN | Sonic hedgehog protein | 9 | 8.4 | 1.56 | <0.001 | 1.89 | <0.001 | 1.44 | 0.001 |
| SHOT1_HUMAN | Shootin-1 | 1 | 1.4 | 1.43 | <0.001 | 1.38 | <0.001 | 0.99 | 0.686 |
| SHOX2_HUMAN | Short stature homeobox protein 2 | 7 | 14.2 | 1.37 | <0.001 | 1.47 | <0.001 | 1.1 | 0.005 |
| SHRM3_HUMAN | Protein Shroom3 | 10 | 3.5 | 1.43 | <0.001 | 1.54 | 0.001 | 1.1 | 0.025 |
| SI1L2_HUMAN | Signal-induced proliferation-associated 1-like protein 2 | 6 | 3.6 | 1.49 | 0.003 | 1.89 | 0.015 | 1.45 | 0.098 |
| SI1L3_HUMAN | Signal-induced proliferation-associated 1-like protein 3 | 6 | 5.8 | 1.56 | <0.001 | 1.98 | 0.001 | 1.57 | 0.020 |
| SIG10_HUMAN | Sialic acid-binding Ig-like lectin 10 | 8 | 3.4 | 1.39 | 0.001 | 1.4 | 0.013 | 1.01 | 0.954 |
| SIK3_HUMAN | Serine/threonine-protein kinase SIK3 | 7 | 6 | 1.38 | <0.001 | 1.36 | 0.001 | 1.01 | 0.686 |
| SIN3B_HUMAN | Paired amphipathic helix protein Sin3b | 7 | 2.2 | 1.41 | <0.001 | 1.72 | 0.001 | 1.45 | 0.067 |
| SIPA1_HUMAN | Signal-induced proliferation-associated protein 1 | 3 | 4.7 | 1.17 | 0.223 | 0.86 | 0.563 | 0.79 | 0.036 |
| SKOR1_HUMAN | SKI family transcriptional corepressor 1 | 15 | 11.7 | 1.49 | <0.001 | 1.9 | 0.014 | 1.43 | 0.144 |
| SMBT1_HUMAN | Scm-like with four MBT domains protein 1 | 2 | 4 | 1.68 | 0.003 | 1.61 | 0.003 | 1.27 | 0.009 |
| SMG1_HUMAN | Serine/threonine-protein kinase SMG1 | 13 | 3.6 | 1.43 | <0.001 | 1.69 | 0.003 | 1.25 | 0.097 |
| SMRC1_HUMAN | SWI/SNF complex subunit SMARCC1 | 4 | 6.7 | 1.48 | <0.001 | 1.65 | <0.001 | 1.23 | <0.001 |
| SND1_HUMAN | Staphylococcal nuclease domain-containing protein 1 | 6 | 6.8 | 1.4 | 0.002 | 1.45 | <0.001 | 1.02 | 0.559 |
| SNED1_HUMAN | Sushi; nidogen and EGF-like domain-containing protein 1 | 9 | 7.1 | 1.5 | <0.001 | 1.53 | <0.001 | 1.15 | <0.001 |
| SON_HUMAN | Protein SON | 37 | 7.5 | 1.45 | <0.001 | 1.77 | <0.001 | 1.33 | <0.001 |
| SOX3_HUMAN | Transcription factor SOX-3 | 9 | 22.4 | 1.25 | 0.001 | 1.4 | <0.001 | 1.01 | 0.903 |
| SOX7_HUMAN | Transcription factor SOX-7 | 1 | 3.4 | 1.46 | <0.001 | 2.05 | <0.001 | 1.93 | 0.024 |
| SP16H_HUMAN | FACT complex subunit SPT16 | 5 | 6.1 | 1.46 | <0.001 | 1.45 | 0.003 | 1.03 | 0.723 |
| SPEG_HUMAN | Striated muscle preferentially expressed protein kinase | 7 | 1.9 | 1.43 | <0.001 | 1.6 | <0.001 | 1.14 | 0.034 |
| SPG7_HUMAN | Paraplegin | 7 | 5 | 1.41 | <0.001 | 1.45 | <0.001 | 1.02 | 0.070 |
| SPIR1_HUMAN | Protein spire homolog 1 | 4 | 3.3 | 1.29 | 0.003 | 1.55 | 0.001 | 1.12 | 0.114 |
| SPT5H_HUMAN | Transcription elongation factor SPT5 | 10 | 5.8 | 1.31 | 0.001 | 1.64 | <0.001 | 1.25 | 0.026 |
| SPTA1_HUMAN | Spectrin alpha chain; erythrocytic 1 | 8 | 2.7 | 1.46 | <0.001 | 1.76 | 0.003 | 1.29 | 0.106 |
| SPTN2_HUMAN | Spectrin beta chain; non-erythrocytic 2 | 11 | 6.4 | 1.47 | <0.001 | 1.61 | 0.001 | 1.12 | 0.001 |
| SPTN5_HUMAN | Spectrin beta chain; non-erythrocytic 5 | 13 | 4.8 | 1.45 | <0.001 | 1.7 | <0.001 | 1.24 | 0.015 |
| SRBS2_HUMAN | Sorbin and SH3 domain-containing protein 2 | 3 | 4.1 | 1.35 | 0.140 | 2.02 | 0.006 | 1.72 | 0.007 |
| SRCN1_HUMAN | SRC kinase signaling inhibitor 1 | 5 | 7.4 | 1.23 | 0.046 | 2.2 | 0.039 | 2.42 | 0.172 |
| SRF_HUMAN | Serum response factor | 8 | 7.3 | 1.43 | <0.001 | 1.75 | <0.001 | 1.29 | 0.004 |
| SRRM2_HUMAN | Serine/arginine repetitive matrix protein 2 | 24 | 10.9 | 1.55 | <0.001 | 1.76 | <0.001 | 1.26 | 0.006 |
| SSPO_HUMAN | SCO-spondin | 2 | 0.3 | 1.05 | 0.845 | 1.56 | <0.001 | 1.22 | 0.102 |
| STAB1_HUMAN | Stabilin-1 | 8 | 5.6 | 1.45 | 0.001 | 1.72 | 0.001 | 1.29 | 0.019 |
| STB5L_HUMAN | Syntaxin-binding protein 5-like | 4 | 4.8 | 1.47 | 0.002 | 1.64 | 0.003 | 1.24 | 0.051 |
| STRP2_HUMAN | Striatin-interacting protein 2 | 8 | 5.8 | 1.47 | 0.005 | 3 | 0.055 | 2.57 | 0.149 |
| SUZ12_HUMAN | Polycomb protein SUZ12 | 4 | 7.2 | 1.26 | <0.001 | 1.36 | <0.001 | 0.97 | 0.280 |
| SVIL_HUMAN | Supervillin | 8 | 3.5 | 1.41 | <0.001 | 1.54 | 0.001 | 1.11 | 0.251 |
| SYDE2_HUMAN | Rho GTPase-activating protein SYDE2 | 8 | 10.2 | 1.13 | 0.516 | 1.68 | 0.020 | 1.3 | 0.106 |
| SYGP1_HUMAN | Ras/Rap GTPase-activating protein SynGAP | 10 | 8 | 1.53 | <0.001 | 1.68 | 0.001 | 1.23 | 0.034 |
| SYNJ1_HUMAN | Synaptojanin-1 | 8 | 6.4 | 1.39 | <0.001 | 1.62 | <0.001 | 1.3 | 0.057 |
| TACC1_HUMAN | Transforming acidic coiled-coil-containing protein 1 | 4 | 9.7 | 1.44 | 0.010 | 1.87 | 0.004 | 1.42 | 0.047 |
| TACC2_HUMAN | Transforming acidic coiled-coil-containing protein 2 | 12 | 6.4 | 1.46 | <0.001 | 1.67 | <0.001 | 1.25 | <0.001 |
| TACC3_HUMAN | Transforming acidic coiled-coil-containing protein 3 | 2 | 3.5 | 1.66 | 0.001 | 2.13 | 0.017 | 1.73 | 0.059 |
| TAGL2_HUMAN | Transgelin-2 | 10 | 26.6 | 1.5 | 0.001 | 1.74 | <0.001 | 1.26 | 0.019 |
| TAL1_HUMAN | T-cell acute lymphocytic leukemia protein 1 | 3 | 16.3 | 1.62 | <0.001 | 1.94 | <0.001 | 1.48 | 0.001 |
| TARA_HUMAN | TRIO and F-actin-binding protein | 5 | 2.7 | 1.32 | <0.001 | 1.71 | 0.034 | 1.28 | 0.174 |
| TBA1A_HUMAN | Tubulin alpha-1A chain | 6 | 8 | 1.46 | <0.001 | 1.51 | <0.001 | 1.11 | 0.003 |
| TBX2_HUMAN | T-box transcription factor TBX2 | 3 | 4.5 | 1.28 | <0.001 | 0.96 | 0.304 | 0.46 | 0.017 |
| TCAF1_HUMAN | TRPM8 channel-associated factor 1 | 4 | 5.5 | 1.28 | <0.001 | 1.74 | 0.001 | 1.36 | <0.001 |
| TCF20_HUMAN | Transcription factor 20 | 2 | 1.6 | 1.64 | 0.011 | 1.75 | 0.002 | 1.37 | 0.088 |
| TDRD3_HUMAN | Tudor domain-containing protein 3 | 6 | 9.1 | 1.48 | <0.001 | 1.8 | <0.001 | 1.37 | <0.001 |
| TDRD9_HUMAN | Putative ATP-dependent RNA helicase TDRD9 | 7 | 7.7 | 1.2 | 0.029 | 1.65 | 0.051 | 1.27 | 0.329 |
| TEN2_HUMAN | Teneurin-2 | 5 | 1.5 | 1.52 | 0.008 | 1.81 | 0.003 | 1.43 | 0.001 |
| TENX_HUMAN | Tenascin-X | 16 | 4.9 | 1.45 | <0.001 | 1.71 | <0.001 | 1.24 | 0.008 |
| TERA_HUMAN | Transitional endoplasmic reticulum ATPase | 5 | 7.9 | 1.55 | <0.001 | 1.77 | 0.002 | 1.31 | 0.028 |
| TET2_HUMAN | Methylcytosine dioxygenase TET2 | 4 | 2.3 | 1.16 | 0.044 | 1.35 | 0.001 | 1 | 1.001 |
| TGFR2_HUMAN | TGF-beta receptor type-2 | 4 | 8.5 | 1.08 | 0.747 | 1.51 | 0.004 | 1.23 | <0.001 |
| TICRR_HUMAN | Treslin | 8 | 4.5 | 1.44 | <0.001 | 1.68 | 0.001 | 1.24 | 0.003 |
| TIE2_HUMAN | Angiopoietin-1 receptor | 3 | 1.7 | 1.56 | 0.001 | 1.75 | 0.001 | 1.32 | 0.038 |
| TIF1B_HUMAN | Transcription intermediary factor 1-beta | 6 | 7.4 | 1.41 | 0.001 | 1.6 | 0.007 | 1.24 | 0.043 |
| TIM_HUMAN | Protein timeless homolog | 4 | 4.7 | 1.37 | 0.002 | 1.49 | <0.001 | 1.09 | 0.017 |
| TLK1_HUMAN | Serine/threonine-protein kinase tousled-like 1 | 6 | 5.1 | 1.31 | 0.003 | 1.46 | <0.001 | 1.16 | 0.034 |
| TLN1_HUMAN | Talin-1 | 56 | 16.6 | 1.59 | <0.001 | 1.77 | 0.001 | 1.25 | 0.031 |
| TLN2_HUMAN | Talin-2 | 8 | 3.4 | 1.47 | <0.001 | 1.73 | <0.001 | 1.31 | 0.002 |
| TNAP3_HUMAN | Tumor necrosis factor alpha-induced protein 3 | 1 | 2.5 | 1.55 | 0.019 | 1.51 | <0.001 | 1.04 | 0.589 |
| TNK1_HUMAN | Non-receptor tyrosine-protein kinase TNK1 | 6 | 15.6 | 1.29 | 0.069 | 1.53 | 0.201 | 1.07 | 0.843 |
| TNR6B_HUMAN | Trinucleotide repeat-containing gene 6B protein | 3 | 2.3 | 1.46 | 0.001 | 1.82 | <0.001 | 1.49 | <0.001 |
| TNR6C_HUMAN | Trinucleotide repeat-containing gene 6C protein | 9 | 8.4 | 1.4 | <0.001 | 1.89 | <0.001 | 1.49 | <0.001 |
| TNS2_HUMAN | Tensin-2 | 7 | 7.4 | 1.35 | 0.001 | 2.04 | 0.014 | 1.6 | 0.033 |
| TP53B_HUMAN | Tumor suppressor p53-binding protein 1 | 10 | 5.2 | 1.42 | 0.002 | 1.75 | <0.001 | 1.28 | <0.001 |
| TPM1_HUMAN | Tropomyosin alpha-1 chain | 7 | 8.8 | 1.24 | 0.001 | 1.29 | 0.031 | 0.89 | 0.209 |
| TPM2_HUMAN | Tropomyosin beta chain | 3 | 8.8 | 1.4 | <0.001 | 2.55 | 0.043 | 2.13 | 0.135 |
| TPM4_HUMAN | Tropomyosin alpha-4 chain | 8 | 19.4 | 1.51 | 0.001 | 1.78 | <0.001 | 1.35 | 0.003 |
| TR10A_HUMAN | Tumor necrosis factor receptor superfamily member 10A | 2 | 7.3 | 1.41 | 0.001 | 1.7 | <0.001 | 1.2 | 0.023 |
| TRA2A_HUMAN | Transformer-2 protein homolog alpha | 12 | 15.2 | 1.44 | <0.001 | 1.62 | <0.001 | 1.19 | 0.013 |
| TRIO_HUMAN | Triple functional domain protein | 7 | 2.8 | 1.48 | <0.001 | 1.58 | 0.008 | 1.14 | 0.364 |
| TRRAP_HUMAN | Transformation/transcription domain-associated protein | 6 | 1.4 | 1.76 | 0.016 | 2.03 | 0.010 | 2.11 | 0.148 |
| TSP1_HUMAN | Thrombospondin-1 | 8 | 3.8 | 1.72 | 0.006 | 1.99 | 0.002 | 1.48 | 0.058 |
| TTBK2_HUMAN | Tau-tubulin kinase 2 | 6 | 5.9 | 1.35 | 0.003 | 1.54 | <0.001 | 1.05 | 0.176 |
| TWST1_HUMAN | Twist-related protein 1 | 3 | 24.3 | 1.49 | 0.001 | 1.54 | 0.047 | 1.07 | 0.759 |
| TYPH_HUMAN | Thymidine phosphorylase | 3 | 10 | 1.42 | <0.001 | 1.64 | 0.002 | 1.17 | 0.048 |
| U520_HUMAN | U5 small nuclear ribonucleoprotein 200 kDa helicase | 8 | 2.7 | 1.49 | <0.001 | 1.64 | <0.001 | 1.16 | 0.008 |
| UBA1_HUMAN | Ubiquitin-like modifier-activating enzyme 1 | 5 | 5.2 | 1.39 | <0.001 | 1.6 | <0.001 | 1.12 | 0.064 |
| UBP34_HUMAN | Ubiquitin carboxyl-terminal hydrolase 34 | 6 | 1.9 | 1.42 | <0.001 | 1.5 | <0.001 | 1.04 | 0.467 |
| UBR5_HUMAN | E3 ubiquitin-protein ligase UBR5 | 8 | 3.9 | 1.44 | <0.001 | 1.64 | <0.001 | 1.24 | 0.013 |
| ULK4_HUMAN | Serine/threonine-protein kinase ULK4 | 4 | 4.6 | 1.37 | <0.001 | 1.57 | 0.001 | 1.14 | 0.016 |
| UN13A_HUMAN | Protein unc-13 homolog A | 6 | 1.9 | 1.35 | <0.001 | 1.55 | 0.003 | 1.07 | 0.289 |
| UN13C_HUMAN | Protein unc-13 homolog C | 16 | 4.4 | 1.48 | <0.001 | 1.85 | 0.009 | 1.43 | 0.103 |
| UNC5D_HUMAN | Netrin receptor UNC5D | 13 | 5.1 | 1.27 | 0.096 | 1.53 | 0.012 | 1.18 | 0.001 |
| USP9X_HUMAN | Probable ubiquitin carboxyl-terminal hydrolase FAF-X | 6 | 2.9 | 1.56 | 0.001 | 1.79 | 0.001 | 1.29 | 0.047 |
| UVRAG_HUMAN | UV radiation resistance-associated gene protein | 7 | 8.4 | 1.62 | 0.002 | 1.67 | <0.001 | 1.29 | 0.028 |
| VASH1_HUMAN | Vasohibin-1 | 3 | 11.2 | 1.15 | 0.579 | 1.78 | 0.019 | 1.36 | 0.115 |
| VASP_HUMAN | Vasodilator-stimulated phosphoprotein | 6 | 11.6 | 1.54 | 0.002 | 1.7 | <0.001 | 1.24 | 0.005 |
| VINC_HUMAN | Vinculin | 32 | 12.5 | 1.46 | 0.001 | 1.73 | 0.001 | 1.28 | 0.021 |
| VIR_HUMAN | Protein virilizer homolog | 7 | 5.3 | 1.46 | <0.001 | 1.8 | <0.001 | 1.36 | 0.028 |
| VPRBP_HUMAN | Protein VPRBP | 3 | 4.4 | 1.29 | <0.001 | 1.36 | 0.001 | 1.01 | 0.826 |
| WBS22_HUMAN | Probable 18S rRNA (guanine-N(7))-methyltransferase | 4 | 6.4 | 1.41 | 0.005 | 1.55 | 0.082 | 1.07 | 0.815 |
| WDR33_HUMAN | pre-mRNA 3~ end processing protein WDR33 | 9 | 3.3 | 1.48 | <0.001 | 1.74 | 0.001 | 1.3 | 0.021 |
| WDR76_HUMAN | WD repeat-containing protein 76 | 6 | 10.5 | 1.48 | <0.001 | 1.79 | <0.001 | 1.39 | 0.004 |
| WIPF1_HUMAN | WAS/WASL-interacting protein family member 1 | 8 | 25.8 | 1.51 | 0.005 | 1.88 | <0.001 | 1.56 | 0.001 |
| WIPF2_HUMAN | WAS/WASL-interacting protein family member | 5 | 12 | 1.62 | <0.001 | 1.83 | 0.021 | 1.39 | 0.221 |
| WIZ_HUMAN | Protein Wiz | 9 | 3.2 | 1.42 | <0.001 | 1.86 | <0.001 | 1.44 | 0.003 |
| WN10A_HUMAN | Protein Wnt-10a | 1 | 5.5 | 2.75 | <0.001 | 7.46 | <0.001 | 8.15 | <0.001 |
| WNK1_HUMAN | Serine/threonine-protein kinase WNK1 | 9 | 5.3 | 1.69 | 0.006 | 2.05 | 0.016 | 1.6 | 0.124 |
| WNT2B_HUMAN | Protein Wnt-2b | 2 | 8.7 | 1.38 | 0.003 | 0.92 | 0.581 | 0.68 | 0.172 |
| WTIP_HUMAN | Wilms tumor protein 1-interacting protein | 8 | 15.6 | 1.42 | <0.001 | 1.86 | <0.001 | 1.33 | <0.001 |
| WWC2_HUMAN | Protein WWC2 | 7 | 6.9 | 1.48 | <0.001 | 1.61 | <0.001 | 1.15 | <0.001 |
| XIRP1_HUMAN | Xin actin-binding repeat-containing protein 1 | 2 | 2.7 | 1.42 | 0.004 | 1.86 | <0.001 | 1.38 | 0.002 |
| XIRP2_HUMAN | Xin actin-binding repeat-containing protein 2 | 12 | 4.6 | 1.52 | <0.001 | 1.82 | <0.001 | 1.35 | 0.005 |
| XPF_HUMAN | DNA repair endonuclease XPF | 5 | 7.9 | 1.46 | 0.003 | 1.74 | 0.006 | 1.36 | 0.001 |
| YTDC2_HUMAN | Probable ATP-dependent RNA helicase YTHDC2 | 12 | 6 | 1.39 | 0.001 | 1.51 | 0.001 | 1.09 | 0.001 |
| ZBED3_HUMAN | Zinc finger BED domain-containing protein 3 | 5 | 16.2 | 1.53 | <0.001 | 1.92 | <0.001 | 1.42 | <0.001 |
| ZBTB4_HUMAN | Zinc finger and BTB domain-containing protein 4 | 10 | 8.1 | 1.45 | <0.001 | 1.71 | <0.001 | 1.33 | <0.001 |
| ZCHC8_HUMAN | Zinc finger CCHC domain-containing protein 8 | 8 | 11.3 | 1.53 | <0.001 | 1.64 | 0.008 | 1.18 | 0.154 |
| ZFHX3_HUMAN | Zinc finger homeobox protein 3 | 6 | 2.1 | 1.5 | 0.008 | 1.6 | 0.010 | 1.19 | 0.309 |
| ZMIZ2_HUMAN | Zinc finger MIZ domain-containing protein 2 | 1 | 3.4 | 1.12 | 0.260 | 1.75 | 0.005 | 1.82 | 0.005 |
| ZMYM3_HUMAN | Zinc finger MYM-type protein 3 | 3 | 3.7 | 1.52 | 0.008 | 1.74 | 0.001 | 1.46 | 0.040 |
| ZN281_HUMAN | Zinc finger protein 281 | 3 | 4.2 | 1.34 | <0.001 | 1.24 | 0.017 | 1.15 | 0.462 |
| ZN292_HUMAN | Zinc finger protein 292 | 12 | 5.9 | 1.35 | 0.009 | 1.83 | <0.001 | 1.28 | 0.005 |
| ZN296_HUMAN | Zinc finger protein 296 | 4 | 9.5 | 1.49 | <0.001 | 1.79 | 0.001 | 1.42 | 0.010 |
| ZN462_HUMAN | Zinc finger protein 462 | 9 | 3.3 | 1.47 | <0.001 | 1.78 | 0.002 | 1.3 | 0.050 |
| ZN703_HUMAN | Zinc finger protein 703 | 2 | 9.8 | 1.37 | <0.001 | 1.97 | 0.002 | 1.56 | 0.022 |
| ZNRF3_HUMAN | E3 ubiquitin-protein ligase ZNRF3 | 3 | 3.4 | 1.56 | 0.011 | 2.01 | 0.028 | 1.54 | 0.188 |
| ZO2_HUMAN | Tight junction protein ZO-2 | 6 | 3.4 | 1.52 | <0.001 | 1.77 | <0.001 | 1.36 | 0.028 |

**Table S3: Important network properties of gene product interactions in three HNLRA dose groups** relative to NLNRA dose groups

| **Sl. No.** | **Property** | **Value** | | |
| --- | --- | --- | --- | --- |
|  |  | **Group II** | **Group III** | **Group IV** |
|  | Nodes in full interactome | 16100 | 16100 | 16100 |
|  | Edges in full interactome | 141080 | 141080 | 141080 |
|  | Total no. of genes | 1460 | 1471 | 692 |
|  | No. of gene products in subgraph | 1106 | 1126 | 528 |
|  | No. of gene products interactions in subgraph | 1703 | 1780 | 497 |
|  | Density of subgraph | 0.00279 | 0.0028 | 0.0036 |
|  | No. of clusters in subgraph | 486 | 495 | 324 |
|  | No. of gene products in subgraph Giant Cluster | 587 | 604 | 178 |
|  | Giant Cluster Significance | Z-score:5.5013,  P-value:0.0001 | Z-score:5.6686,  P-value:0.0001 | Z-score:3.6015,  P-value:0.0001 |

**Table S4**: **Topological parameters of PPI networks**. Top 10 proteins in the three HLNRA dose groups relative to NLNRA dose group with highest Degree Centrality (DC), Betweeness Centrality (BC), Closeness Centrality (CC), Clustering and their log_2_ Fold Change (FC) values.

| **Gene** | **Uniprot** | **Degree** | **DC** | **BC** | **CC** | **Clustering** | **log_2_FC** |
| --- | --- | --- | --- | --- | --- | --- | --- |
| **Group II** | | | | | | | |
| YWHAZ | P63104 | 70 | 0.063 | 0.085 | 0.218 | 0.032 | 0.546 |
| PRKACA | P17612-2 | 49 | 0.044 | 0.048 | 0.195 | 0.016 | 0.465 |
| ACTB | P60709 | 46 | 0.042 | 0.029 | 0.19 | 0.064 | 0.595 |
| CHEK1 | O14757 | 41 | 0.037 | 0.024 | 0.181 | 0.026 | 0.401 |
| HNRNPU | Q00839 | 39 | 0.035 | 0.008 | 0.184 | 0.31 | 0.526 |
| U2AF1 | Q01081 | 38 | 0.034 | 0.003 | 0.168 | 0.405 | 0.496 |
| CDC5L | Q99459 | 37 | 0.033 | 0.015 | 0.19 | 0.344 | 0.444 |
| MAPK1 | P28482 | 35 | 0.032 | 0.021 | 0.168 | 0.011 | 0.379 |
| HSPA8 | P11142 | 33 | 0.03 | 0.025 | 0.192 | 0.131 | 0.595 |
| PPP1CA | P62136 | 33 | 0.03 | 0.022 | 0.186 | 0.075 | 0.678 |
| **Group III** | | | | | | | |
| YWHAZ | P63104 | 71 | 0.063 | 0.08 | 0.22 | 0.034 | 0.774 |
| ACTB | P60709 | 49 | 0.044 | 0.027 | 0.192 | 0.068 | 0.322 |
| PRKACA | P17612-2 | 48 | 0.043 | 0.04 | 0.195 | 0.015 | 0.287 |
| CHEK1 | O14757 | 42 | 0.037 | 0.024 | 0.185 | 0.024 | 0.566 |
| HSPA8 | P11142 | 39 | 0.035 | 0.03 | 0.198 | 0.122 | 0.872 |
| MAPK1 | P28482 | 39 | 0.035 | 0.02 | 0.173 | 0.009 | 0.687 |
| HNRNPU | Q00839 | 38 | 0.034 | 0.005 | 0.184 | 0.319 | 0.872 |
| CDC5L | Q99459 | 36 | 0.032 | 0.014 | 0.19 | 0.34 | 0.66 |
| U2AF1 | Q01081 | 36 | 0.032 | 0.003 | 0.169 | 0.408 | 0.74 |
| HNRNPM | P52272 | 33 | 0.029 | 0.006 | 0.174 | 0.371 | 0.696 |
| **Group IV** | | | | | | | |
| YWHAZ | P63104 | 32 | 0.061 | 0.041 | 0.142 | 0.051 | 0.401 |
| ACTB | P60709 | 26 | 0.049 | 0.029 | 0.132 | 0.076 | -0.269 |
| HSPA8 | P11142 | 21 | 0.04 | 0.02 | 0.131 | 0.129 | 0.496 |
| U2AF1 | Q01081 | 20 | 0.038 | 0.005 | 0.108 | 0.411 | 0.31 |
| SF3B3 | Q15393 | 17 | 0.032 | 0.005 | 0.114 | 0.456 | 0.345 |
| HNRNPU | Q00839 | 17 | 0.032 | 0.006 | 0.119 | 0.338 | 0.454 |
| SF3B4 | Q15427 | 16 | 0.03 | 0.005 | 0.112 | 0.467 | 0.475 |
| HNRNPA1 | P09651 | 15 | 0.028 | 0.003 | 0.118 | 0.449 | 0.444 |
| CD2BP2 | O95400 | 13 | 0.025 | 0.002 | 0.102 | 0.628 | 0.632 |
| ACTN4 | O43707 | 13 | 0.025 | 0.014 | 0.124 | 0.154 | 0.516 |

**Table S5: Characteristics of samples used for RT-PCR**

| **Area** | **Defined Dose Groups** | **No. of samples** | **Actual dose range**  **in mGy/y**  **(Mean dose ± SD)** | **Age**  **(mean ± SD) in years** |
| --- | --- | --- | --- | --- |
| **NLNRA**  (Control) | **Group I**  ≤1.50 mGy/y | 5 | 1.23 - 1.30  Mean dose: 1.25 ± 0.03 | 32.0 ± 5.39 |
| **HLNRA** | **Group II**  1.51- 5.0 mGy/y | 5 | 3.11 – 3.53  Mean dose: 3.30 ± 0.19 | 38.8 ± 6.30 |
|  | **Group III**  5.01- 14.0 mGy/y | 5 | 7.98 - 13.69  Mean dose: 10.98 ± 2.58 | 44.8 ± 11.08 |
|  | **Group IV**  ≥ 14.01 mGy/y | Could not be collected | | |

**Table S6: List of primers used for RT-PCR**

| **Gene** | **Nucleotide sequence 5’→3’** |
| --- | --- |
| *ATR* | F-ACGACTCGCTGAACTGTACG |
|  | R-TGGTGAACATCACCCTTGG |
| *BLM* | F-AGTGTTGTGGCCGTTGTTTC |
|  | R-TGCTCAGAAGCTCTTGCACT |
| *ERCC4* | F-CCACTGACACTCGGAAAGC |
|  | R-CACGCATATCCACAACTATGC |
| *FANCA* | F-GGGGACGACGATGACAAT |
|  | R-ATGGTGAACCATGTGCAGAA |
| *FANCI* | F-CAGAATCAAGCAGTGAAAGGAA |
|  | R-AGGGGGAACCTTTGAAGATG |
| *FANCM* | F-TCTGCAGTTCTCTTGCCTACTG |
|  | R-AGGCCTCGGGAACTTACAAT |
| *MLH1* | F-AGGAAGAACGTGAGCACGAG |
|  | R-CGTCTAGATGCTCAACGGAAG |
| *ATRX* | F-GAGCCCTGTCAGCAATGAGT |
|  | R-GCTGTCACACTGTTTGTTGCT |
| *CHD8* | F-TGAAGACGTAGCCATCTTGC |
|  | R-ACATGCCGATGTCCTTGG |
| *EMSY* | F-TCAGAGAAACAGACGGCAAG |
|  | R-GCGCCCCTCAAAAGTTATC |
| *CSN5* | F-ATGCTCAGGCTGCTGCATA |
|  | R-GATACCACCCGATTGCATTT |
| *MK01* | F-AGATTCCAGCCAGGATACAGAT |
|  | R-AGACAGGACCAGGGGTCAA |
| *MINK1* | F-CTGGACGACATCGACCTGT |
|  | R-CCACCTCCACAAGCTCAAAG |
| *SMG1* | F-TTCTGGAAGACATGGAAGCA |
|  | R-CACTGATGGAGGAGGGACAT |
| *DAPK1* | F-CTGGCTTCTAAGCCCACAGT |
|  | R-GGCTCCTCACACTCACGTTC |
| *ZNRF3* | F-GCTCGAGCAAGGATCCAG |
|  | R-CAAGGAGACCACGACGAAG |
| *β-ACTIN* | F-ATA CCC CTC GTA GAT GGG CAC |
|  | R-GAG AAA ATC TGG CACCAC ACC |
| *GAPDH* | F-GGCATCCTGGGCTACACT |
|  | R-GAGTGGGTGTCGCTGTTG |
