# Supplementary figures and images for: Chronic exposure of humans to high level natural background radiation leads to robust expression of protective stress response proteins
Source: Sci Rep. 2021 Jan 19;11:1777. doi: 10.1038/s41598-020-80405-y (PMC7815775; doi:10.1038/s41598-020-80405-y)

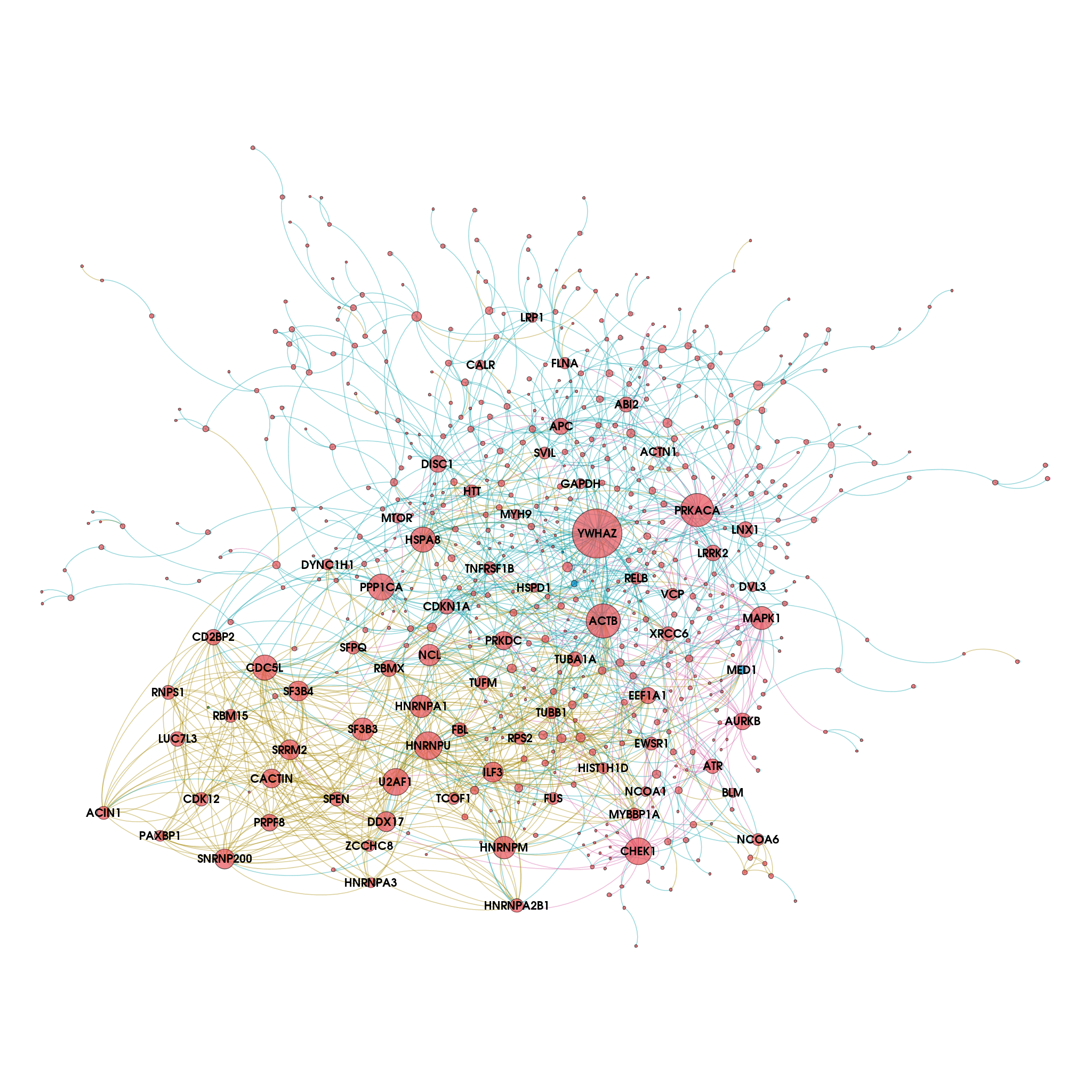

Supplement: Supplementary file 2 — Supplementary Information 2a. [file 41598_2020_80405_MOESM2_ESM.png]

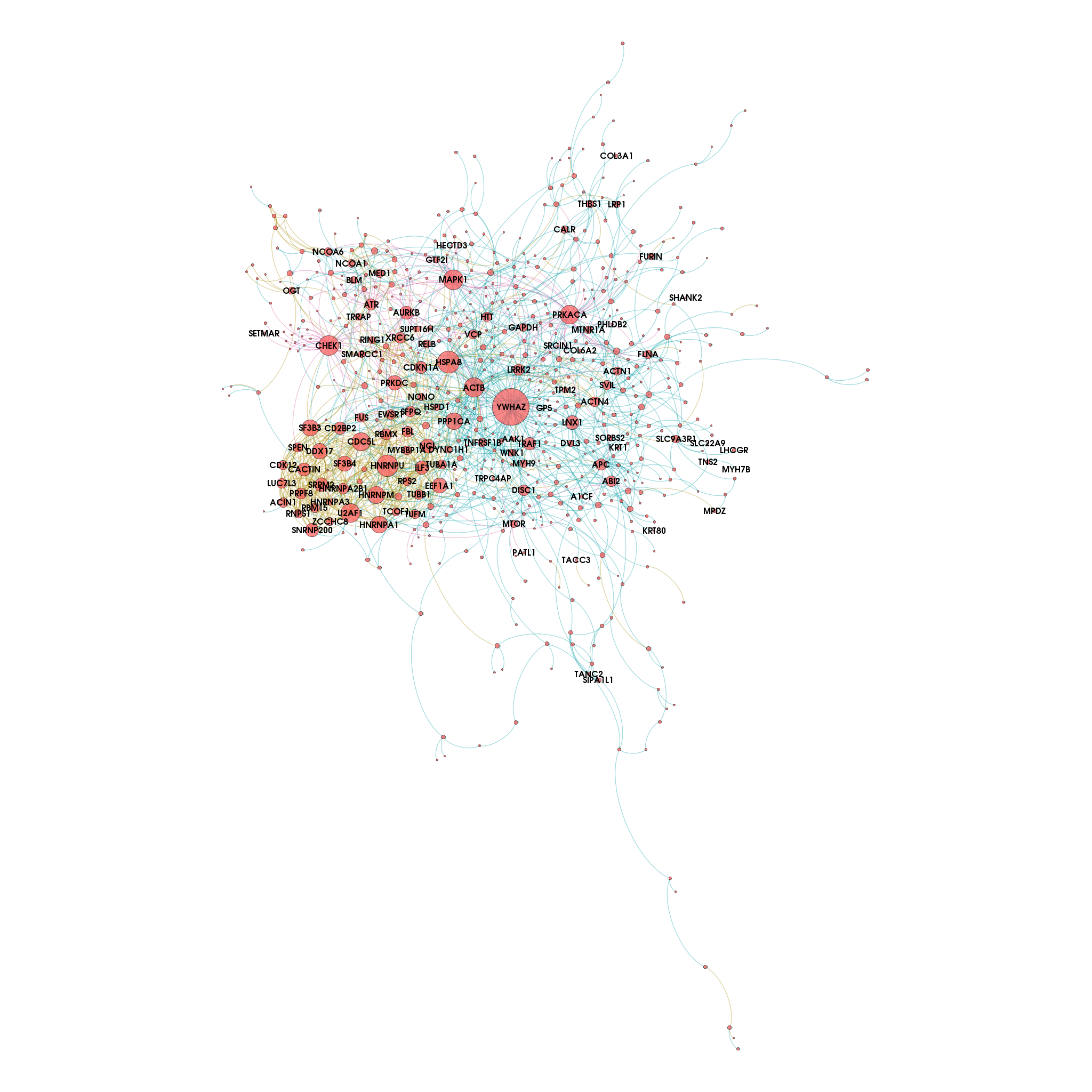

Supplement: Supplementary file 3 — Supplementary Information 2b. [file 41598_2020_80405_MOESM3_ESM.png]

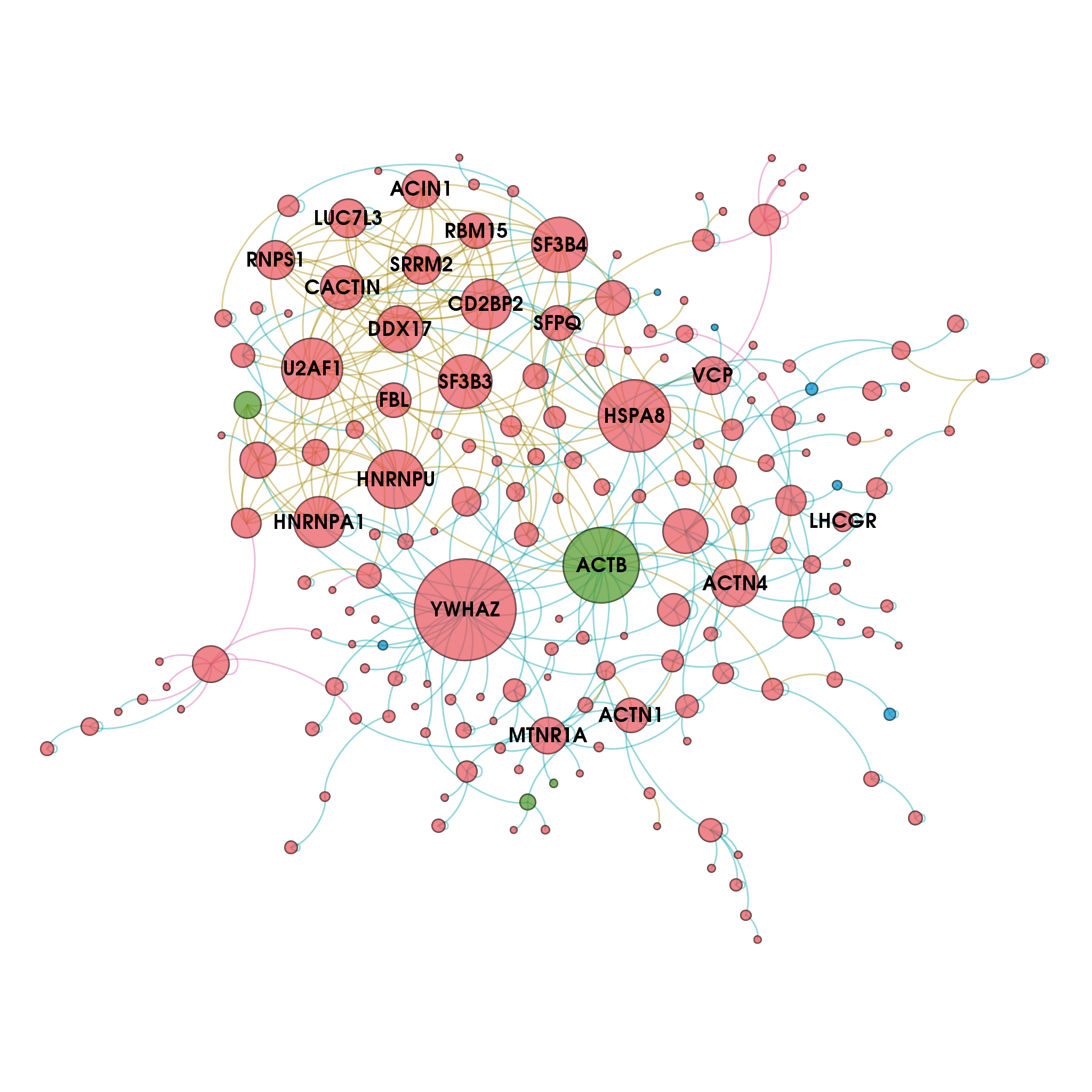

Supplement: Supplementary file 4 — Supplementary Information 2c. [file 41598_2020_80405_MOESM4_ESM.png]
